# Agonist Binding to Chemosensory Receptors: A Systematic Bioinformatics Analysis

**DOI:** 10.3389/fmolb.2017.00063

**Published:** 2017-09-06

**Authors:** Fabrizio Fierro, Eda Suku, Mercedes Alfonso-Prieto, Alejandro Giorgetti, Sven Cichon, Paolo Carloni

**Affiliations:** ^1^Computational Biomedicine, Institute for Advanced Simulation IAS-5 and Institute of Neuroscience and Medicine INM-9, Forschungszentrum Jülich Jülich, Germany; ^2^Department of Biotechnology, University of Verona Verona, Italy; ^3^Cécile and Oskar Vogt Institute for Brain Research, Medical Faculty, Heinrich Heine University Düsseldorf Düsseldorf, Germany; ^4^Institute of Neuroscience and Medicine INM-1, Forschungszentrum Jülich Jülich, Germany; ^5^Institute for Human Genetics, Department of Genomics, Life&Brain Center, University of Bonn Bonn, Germany; ^6^Division of Medical Genetics, Department of Biomedicine, University of Basel Basel, Switzerland; ^7^Department of Physics, Rheinisch-Westfälische Technische Hochschule Aachen Aachen, Germany; ^8^VNU Key Laboratory “Multiscale Simulation of Complex Systems”, VNU University of Science, Vietnam National University Hanoi, Vietnam

**Keywords:** G-protein coupled receptor, chemosensory receptor, bitter taste receptor, odorant receptor, bioinformatics, homology modeling, molecular docking, molecular mechanics/coarse grained simulations

## Abstract

Human G-protein coupled receptors (hGPCRs) constitute a large and highly pharmaceutically relevant membrane receptor superfamily. About half of the hGPCRs' family members are chemosensory receptors, involved in bitter taste and olfaction, along with a variety of other physiological processes. Hence these receptors constitute promising targets for pharmaceutical intervention. Molecular modeling has been so far the most important tool to get insights on agonist binding and receptor activation. Here we investigate both aspects by bioinformatics-based predictions across all bitter taste and odorant receptors for which site-directed mutagenesis data are available. First, we observe that state-of-the-art homology modeling combined with previously used docking procedures turned out to reproduce only a limited fraction of ligand/receptor interactions inferred by experiments. This is most probably caused by the low sequence identity with available structural templates, which limits the accuracy of the protein model and in particular of the side-chains' orientations. Methods which transcend the limited sampling of the conformational space of docking may improve the predictions. As an example corroborating this, we review here multi-scale simulations from our lab and show that, for the three complexes studied so far, they significantly enhance the predictive power of the computational approach. Second, our bioinformatics analysis provides support to previous claims that several residues, including those at positions 1.50, 2.50, and 7.52, are involved in receptor activation.

## Introduction

The G-protein coupled receptor (GPCR) superfamily is the largest group of plasma eukaryotic membrane receptors, with about 850 members in the human genome (Fredriksson et al., 2003; Lagerstrom and Schioth, 2008; Tikhonova and Fourmy, 2010). According to the GRAFS classification (Schioth and Fredriksson, 2005), human G-protein coupled receptors (hGPCRs) are divided in five different families, i.e., Rhodopsin-like (or class A), Glutamate (or class C), Adhesion (or class B2), Frizzled (or class F) and Secretin (or class B1). They all share a seven transmembrane (TM) helix bundle shape (Kobilka, 2007; Venkatakrishnan et al., 2013, 2016; Latorraca et al., 2017). Binding of an extracellular agonist (or a photon in the case of rhodopsin) triggers conformational changes in the receptor. This activates intracellular signaling cascades, leading to downstream events. Because of their crucial role for many cellular signaling pathways, hGPCRs are of immense importance in pharmacology, being the target of ~50% of currently FDA approved drugs (Schlyer and Horuk, 2006; Lundstrom, 2009; Salon et al., 2011; Tautermann, 2014; Miao and McCammon, 2016).

Approximately half of the members of the hGPCR superfamily are chemosensory receptors (hChem-GPCRs hereafter) (Takeda et al., 2002). These include odorant receptors (hORs), bitter taste receptors (hTAS2Rs) and sweet and umami taste receptors (Buck and Axel, 1991; Chandrashekar et al., 2006; Yarmolinsky et al., 2009). Here we focus on hORs and hTAS2Rs, because they represent the first and third largest hGPCR subfamilies (with ~400 and ~25 members, respectively) (Lagerstrom and Schioth, 2008; Foster et al., 2014). Although initially found to be responsible for odorant (Buck and Axel, 1991; Zhao et al., 1998; Firestein, 2001, 2005) and bitter taste perception (Adler et al., 2000; Chandrashekar et al., 2000; Matsunami et al., 2000), it is now recognized that hChem-GPCRs participate in other extra-nasal (Ansoleaga et al., 2013; Foster et al., 2014; Abaffy, 2015; Ferrer et al., 2016), and extra-oral (Behrens and Meyerhof, 2011; Shaik et al., 2016; Lu et al., 2017) physiological processes. In addition, hChem-GPCRs are involved in pathological processes (Behrens and Meyerhof, 2011; Ansoleaga et al., 2013; Foster et al., 2014; Abaffy, 2015; Ferrer et al., 2016; Shaik et al., 2016; Lu et al., 2017). Thus, they are emerging as promising targets for pharmaceutical intervention (Foster et al., 2014; Ferrer et al., 2016; Shaik et al., 2016; Lu et al., 2017), as it happened in the past for other GPCRs (Schlyer and Horuk, 2006; Lundstrom, 2009; Salon et al., 2011; Tautermann, 2014; Miao and McCammon, 2016).

hORs belong to class A GPCRs, sharing with the other class A GPCRs several conserved motifs (de March et al., 2015a) (see Table 1). They are expressed in different tissues, from the cilia of olfactory sensory neurons in the nose, to the testis, the gut, the skin, the tongue, leucocytes, thrombocytes, the skeletal muscle, primordial germ cells and oocytes, the atrioventricular node and the brain (Goto et al., 2001; Spehr et al., 2003; Durzynski et al., 2005; Feldmesser et al., 2006; Braun et al., 2007; Jenkins et al., 2009; Breer et al., 2012; Ansoleaga et al., 2013, 2015; Flegel et al., 2013, 2015; Garcia-Esparcia et al., 2013; Wijten et al., 2013; Busse et al., 2014; Grison et al., 2014; Malki et al., 2015; Ko and Park, 2016). Their functions span from olfaction to sperm chemotaxis, to regulation of renal function, to regeneration and migration in muscle cells, or to neuronal regulation (Spehr et al., 2004; Griffin et al., 2009; Pluznick et al., 2009; Grison et al., 2014; Ferrer et al., 2016). hORs are connected to several diseases, including cervical cancer, prostate cancer, pancreatic ductal adenocarcinoma, Creutzfeldt-Jakob's disease, Alzheimer's disease, progressive supranuclear palsy, schizophrenia, and retinitis pigmentosa (Wang et al., 2006; Neuhaus et al., 2009; Kang and Koo, 2012; Zhou et al., 2012; Ansoleaga et al., 2013; Rodriguez et al., 2014; Ma et al., 2015; Guerrero-Flores et al., 2017).

**Table 1:** Shared conserved motifs between Class A GPCRs (Lagerstrom and Schioth, 2008; Venkatakrishnan et al., 2013; Tehan et al., 2014), hTAS2Rs (Pydi et al., 2014a, 2016; Di Pizio et al., 2016), and hORs (de March et al., 2015a).

**TM helix**	**Class A**	**hTAS2Rs**	**hORs**
TM1	N^1.50^xxV^1.53^	N^1.50^xxI^1.53^	G^1.49^N^1.50^xxI^1.53^
TM2	L^2.46^xxxD^2.50^	L^2.46^xxxR^2.50^	L^2.46^S^2.47^xxD^2.50^
TM3	D[E]^3.49^R^3.50^Y^3.51^	L^3.46^xxF^3.49^Y^3.50^xxK^3.53^	D[E]^3.49^R^3.50^Y^3.51^
TM4	W^4.50^	4.50 not conserved	W^4.50^
TM5	– P^5.50^ –	L^5.39^xxS^5.42^L^5.43^ P^5.50^	– 5.50 is not conserved S^5.57^Y^5.58^
TM6	– F^6.44^xxxW^6.48^x P^6.50^	– F^6.44^xxxY^6.48^ 6.50 is not conserved	KAFSTCxSH^6.40^ – 6.50 is not conserved
TM7	N^7.49^P^7.50^xxY^7.53^	H^7.49^S^7.50^xI[V]^7.52^L^7.53^	N^7.49^P^7.50^xI[L]^7.52^Y^7.53^

*Residue positions are indicated using the Ballesteros-Weinstein numbering (Ballesteros and Weinstein, 1995)*.

hTAS2Rs have been suggested either to form a distinct, novel GPCR class, or to belong to class F (Fredriksson et al., 2003), or class A (Nordstrom et al., 2011; Cvicek et al., 2016). The latter hypothesis has been recently corroborated by phylogenetic analyses (Nordstrom et al., 2011), as well as by the observation that several class A motifs are also conserved in hTAS2Rs (Di Pizio et al., 2016; Table 1). hTAS2Rs are located in the tongue and palate epithelium, but also in the gastrointestinal tract, heart, leukocytes, vascular smooth muscle cells, bone marrow derived mesenchymal cells and sinonasal cells of the airway epithelium, and the brain (Hoon et al., 1999; Meyerhof, 2005; Sternini, 2007; Behrens and Meyerhof, 2009; Singh et al., 2011b; Garcia-Esparcia et al., 2013; Lund et al., 2013; Foster et al., 2014; Lee and Cohen, 2014; Manson et al., 2014; Ansoleaga et al., 2015; Malki et al., 2015; Shaik et al., 2016). hTAS2Rs' extra-oral roles include detection of toxins, bronchodilation, and hormone secretion (Janssen et al., 2011; Lee et al., 2012; Robinett et al., 2014). Moreover, polymorphic variants of hTAS2Rs have been found to be correlated to diseases, such as chronic rhinosinusitis and cystic fibrosis, pancreatic cancer, risk of dental caries and vection-induced motion sickness and nausea (Wendell et al., 2010; Benson et al., 2012; Adappa et al., 2014; Gaida et al., 2016; Shaik et al., 2016; Lu et al., 2017).

Cheminformatics methods have provided encouraging results regarding the *in silico* prediction of sensory attributes of chemicals (bitterness or smell) using either machine-learning algorithms, or ligand-based methods, or (binding site) structure-based methods (Bahia et al., 2017; Keller et al., 2017). However, an important limitation is represented by the paucity of the experimental data and by the reliability of the psychophysical tests that these methods often use (Bahia et al., 2017; Keller et al., 2017). Furthermore, it is still not possible to predict the smell quality of a compound from its chemical structure or whether a given molecule has a perceived odor (Keller et al., 2017).

Understanding agonist binding of hChem-GPCRs at the molecular level can provide complementary insights on chemical sensing (Charlier et al., 2013; Di Pizio and Niv, 2014; Suku et al., 2017), as well as offer exciting and unexplored opportunities for drug design (Foster et al., 2014; Ferrer et al., 2016; Shaik et al., 2016; Lu et al., 2017). In addition, it may provide hints on receptors' agonist binding site architecture (Sandal et al., 2015) and activation mechanisms (Lai et al., 2005, 2014; Biarnes et al., 2010; Dai et al., 2011; Singh et al., 2011a; Pydi et al., 2014a; de March et al., 2015b). Because of the lack of experimental structural information, structural insights rely on computations (reviewed in Charlier et al., 2013; Di Pizio and Niv, 2014; Suku et al., 2017). The predictions may be validated against site-directed mutagenesis and functional (agonist dose–response curves) assays (Table 2). By measuring changes in the half maximal effective concentration (EC_50_) values of the ligand upon specific mutations (see Supplementary Table 1), one can pinpoint residues important for ligand binding and/or activation. Nonetheless, the EC_50_ values are measured using downstream signaling effects (e.g., cAMP, Ca^2+^ ions or IP_3_ concentration increase Restrepo et al., 1990; Bruch, 1996; Berridge et al., 2000; Clapp et al., 2001; Matthews and Reisert, 2003), and thus one cannot disentangled whether the observed changes are associated only with ligand binding and/or with the resulting signal transduction cascade caused by receptor activation (Colquhoun, 1998; Strange, 2008, 2010; Williams and Hill, 2009) (see Supplementary Information Section 2.1 for further details).

**Table 2:** Human chemosensory GPCRs (hChem-GPCRs)/agonist complexes for which experimental data are available.

**hChem-GPCR**	**Agonist (charge)**	**Complex abbreviation**	**Reference**
hTAS2R1	dextromethorphan (+1)	T2R1/dmx	Singh et al., 2011a
hTAS2R4	quinine (+1)	T2R4/quin	Pydi et al., 2014b,c
hTASR10	denatonium (+1)	T2R10/dena	Born et al., 2013
	parthenolide (0)	T2R10/parthe	
	strychnine (+1)	T2R10/strych	
hTAS2R16	arbutin (0)	T2R16/arbu	Sakurai et al., 2010
	phenyl-β-D-glucopyranoside (0)	T2R16/phenyl	
	salicin (0)	T2R16/sali	
hTAS2R30	denatonium (+1)	T2R30/dena	Pronin et al., 2004
hTAS2R31	aristolochic acid (−1)	T2R31/aristo	Pronin et al., 2004; Brockhoff et al., 2010
hTAS2R38	phenylthiocarbamide (0)	T2R38/PTC	Biarnes et al., 2010; Marchiori et al., 2013
	propylthiouracil (0)	T2R38/PROP	
hTAS2R43	n-isopropyl-2-methyl-5- nitrobenzenesulfonamide (0)	T2R43/IMNB	Pronin et al., 2004; Brockhoff et al., 2010
	6-nitrosaccharin (0)	T2R43/6-nitro	
hTAS2R46	strychnine (+1)	T2R46/strych	Brockhoff et al., 2010; Sandal et al., 2015
hOR1A1	(*R*)-(–)-carvone (0)	OR1A1/R-carvone	Geithe et al., 2017
	(*S*)-(+)-carvone (0)	OR1A1/S-carvone	
	citronellol (0)	OR1A1/citro	Schmiedeberg et al., 2007
hOR2AG1	amylbutyrate (0)	OR2AG1/amyl	Gelis et al., 2012
hOR2M3	3-mercapto-2-methyl-pentan-1-ol (0)	OR2M3/3-mercapto	Noe et al., 2016
hOR7D4	androstadienone (0)	OR7D4/androste	Keller et al., 2007; Zhuang et al., 2009
	androstenone (0)	OR7D4/androsta	

Hence, validation of the predictions may be in principle carried out (i) by cross-checking whether the residues whose mutations are associated with EC_50_ changes are forming actual interactions with the ligand in the model and/or have an impact on activation and (ii) by predicting new residues involved in binding or activation that are subsequently verified experimentally. In practice, the former mutations are much easier to design than the latter.

Computational approaches aimed at structural predictions of hChem-GPCR/ligand complexes (reviewed in Charlier et al., 2013; Di Pizio and Niv, 2014; Suku et al., 2017) include homology modeling, based on GPCR X-ray structures as templates, along with molecular docking, often guided by information about the putative binding site, as done for other GPCRs (Michino et al., 2009; Kufareva et al., 2011, 2014). Unfortunately, on one hand the sequence identity of hChem-GPCRs with GPCRs for which experimental structural information is available, is <20% (Charlier et al., 2013; Di Pizio and Niv, 2014; Suku et al., 2017). Hence, the resulting homology models have low statistical confidence. In particular, the orientation of the side chains, essential for protein-ligand interactions, is not accurately predicted (Chothia and Lesk, 1986; Baker and Sali, 2001; Eramian et al., 2008; Piccoli et al., 2013; Busato and Giorgetti, 2016). On the other hand, standard docking algorithms, while very successful to predict ligand poses when high resolution experimental structures are used (Michino et al., 2009; Katritch et al., 2010; Kufareva et al., 2011, 2014; Beuming and Sherman, 2012), may also show limited predictive power in the case of hChem-GPCRs (Stary et al., 2007; Biarnes et al., 2010; Launay et al., 2012; Marchiori et al., 2013; Sandal et al., 2015) (see Supplementary Information Section 1.1). These methods usually cannot take fully into account receptor dynamics and hydration (Katritch et al., 2010; Spyrakis et al., 2011; Spyrakis and Cavasotto, 2015), crucial for ligand binding and receptor activation in GPCRs (Pardo et al., 2007; Angel et al., 2009; Nygaard et al., 2010; Latorraca et al., 2017).

Refinement of the hChem-GPCR/ligand complex models have been carried out by molecular dynamics (MD) simulation (Gelis et al., 2012; Lai and Crasto, 2012; Charlier et al., 2013; Marchiori et al., 2013; Lai et al., 2014; Sandal et al., 2015). This approach may alleviate some of the limitations of the bioinformatics procedure (Charlier et al., 2013; Di Pizio and Niv, 2014; Suku et al., 2017). In particular, it allows a more extensive exploration of the conformational space in the presence of the solvent (Spyrakis et al., 2011; Chen, 2015; Spyrakis and Cavasotto, 2015; Broomhead and Soliman, 2017), though at the expense of a higher computational cost.

Here, we investigate for the first time the reliability of bioinformatics/docking predictions, by systematically predicting the structural determinants of ligand binding in hChem-GPCRs for which experimental mutagenesis data are available (Table 2 and Supplementary Table 1). We focus on mutants located in the top half of the receptor, because this is the location of the canonical orthosteric binding site of class A GPCRs, as known from the available crystal structures of ligand/receptor complexes (Venkatakrishnan et al., 2013). We use a state-of-the art homology modeling protocol together with blind molecular docking-based tools used previously for hChem-GPCRs' structural predictions (reviewed in Di Pizio and Niv, 2014). It turns out that only 36% or less of the predictions are consistent with experiment (under the assumption that all of the mutants considered are involved, at least in part, directly or indirectly, in ligand binding). The predictive power varies from system to system in a non-trivial manner. Hence, while bioinformatics/docking-based models constitute an excellent starting point to study ligand binding, they may require structural refinement to improve their agreement with experiment, as well as to increase their predictive power. We show that this is the case for the three systems investigated so far with our multiscale MD simulation approach (Marchiori et al., 2013; Sandal et al., 2015).

We close this investigation by analyzing the experimentally characterized residues that have been suggested to be involved in activation in hChem-GPCRs (Biarnes et al., 2010; Singh et al., 2011a; Pydi et al., 2012, 2014a). These are residues whose mutation causes changes in receptor's response, from abolishing activation to constitutive activation. Using bioinformatics analyses, we provide interesting information regarding a novel conserved hydrophobic position that may be involved in activation of hTAS2Rs, but also hORs and, in general, class A GPCRs. These analyses, together with the phylogenetic tree in reference (Nordstrom et al., 2011) and the conservation of some TM motifs (Table 1; Di Pizio et al., 2016), support the classification of hTAS2Rs as a branch diverging from class A GPCRs.

## Results and discussion

Here we present first an assessment of the quality of models of hChem-GPCRs based on bioinformatics and molecular docking. Next, we show that bioinformatics approaches also corroborate previous suggestions on the role of specific residues of hTAS2Rs for activation.

### Bioinformatics-based binding predictions

Multiple sequence alignments (MSAs) of hTAS2Rs and hORs (see Section 1.2 of the Supplementary Information) were considered for the creation of the Hidden Markov Model (HMM) profiles of both subfamilies. These profiles were then used for template search among the GPCRs with known structure using the GOMoDo pipeline (Sandal et al., 2013). The templates turned out to share a sequence identity of 11–20% with the targets. The best template corresponds to the human class A GPCR β2 adrenoceptor (PDB code: 4LDE, X-ray resolution: 2.79 Å Ring et al., 2013). Indeed, this template is one of the top ranked templates based on the HHsearch output (Soding et al., 2005), in which all the conserved features of the target-template alignment are captured (see Section 1.3 of the Supplementary Information). In addition, the models generated with this template present consistently the best MODELLER quality scores (Melo et al., 2002; Shen and Sali, 2006) for all hChem-GPCRs. Finally, the template is in a fully activated state (Venkatakrishnan et al., 2016), which is expected to be the agonist-bound conformational state; this is particularly important here because all the ligands in Table 2 are agonists. Hence, this template was used to generate all the hChem-GPCR models, ensuring uniformity of the predictions.

Homology models were built following a protocol previously used in references (Marchiori et al., 2013; Sandal et al., 2013, 2015). Agonists' binding modes were predicted using blind docking approaches and programs previously used for hChem-GPCRs (Stary et al., 2007; Launay et al., 2012; Marchiori et al., 2013; Sandal et al., 2015). These include: HADDOCK (Dominguez et al., 2003), AutoDock Vina (Trott and Olson, 2010), and Glide (Friesner et al., 2004).

**Figure 1:**
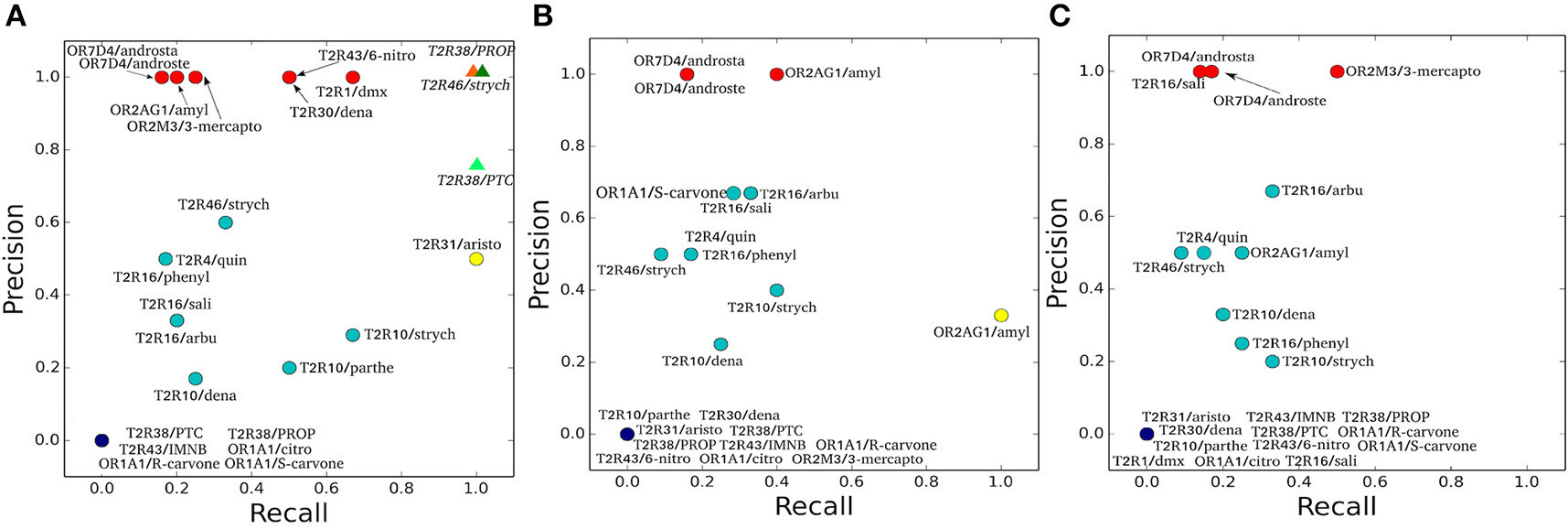
*Precision* and *Recall* plots for the predictions of hChem-GPCR/agonist complexes. **(A-C)** show the HADDOCK (Dominguez et al., 2003), AutoDock Vina (Trott and Olson, 2010), and Glide (Friesner et al., 2004) docking predictions, respectively. The abbreviations used for the hChem-GPCR/agonist complexes are listed in Table 2. Bioinformatics/Docking-based predictions are shown as circles, colored according to the two performance metrics: dark blue (0 precision, 0 recall), red (precision 1, low recall), yellow (low precision, recall 1) and cyan (all the rest, with intermediate precision and recall values). In panel A, MM/CG simulation results (Marchiori et al., 2013; Sandal et al., 2015), started from Haddock docking complexes, are displayed as colored triangles.

To characterize the quality of the binding poses (Supplementary Figures 2–4), precision (PREC) and recall (REC) values are calculated for each of the hChem-GPCR/agonist complex predictions and for each of the docking programs (Figure 1 and Table 3). We find that the bioinformatics results vary from system to system in a non-trivial manner. For instance, the predictions for the same receptor with three different agonists (e.g., hTAS2R10 in complex with denatonium, parthenolide or strychnine) have significantly different values of recall and precision. This is also the case when comparing the docking of the same agonist to two different receptors (such as strychnine bound to hTAS2R10 and hTAS2R46).

**Table 3:** Performance assessment of the computational predictions of hChem-GPCR/agonist complexes, using the docking codes HADDOCK (Dominguez et al., 2003), AutoDock Vina (Trott and Olson, 2010), and Glide (Friesner et al., 2004) and MM/CG simulations (Marchiori et al., 2013; Sandal et al., 2015).

**Test statistics**	**HADDOCK**		**AutoDock Vina**		**Glide**	
**Human bitter taste receptor/agonist complex**	**REC**	**PREC**	**REC**	**PREC**	**REC**	**PREC**
hTAS2R1/dextromethorphan	0.67	1.00	0.33	1.00	0.00	0.00
hTAS2R4/quinine	0.17	0.50	0.17	0.50	0.17	0.50
hTAS2R10/denatonium	0.25	0.17	0.25	0.25	0.20	0.33
hTAS2R10/parthenolide	0.50	0.20	0.00	0.00	0.00	0.00
hTAS2R10/strychnine	0.67	0.29	0.40	0.40	0.33	0.20
hTAS2R16/arbutin	0.20	0.33	0.33	0.67	0.33	0.67
hTAS2R16/phenyl-β-D-glucopyranoside	0.17	0.50	0.17	0.50	0.25	0.25
hTAS2R16/salicin	0.20	0.33	0.33	0.67	0.00	0.00
hTAS2R30/denatonium	0.50	1.00	0.00	0.00	0.00	0.00
hTAS2R31/aristolochic acid	1.00	0.50	0.00	0.00	0.00	0.00
hTAS2R38/phenylthiocarbamide	0.00	0.00	0.00	0.00	0.00	0.00
hTAS2R38/propylthiouracil	0.00	0.00	0.00	0.00	0.00	0.00
hTAS2R43/IMNB	0.00	0.00	0.00	0.00	0.00	0.00
hTAS2R43/6-nitrosaccharin	0.50	1.00	0.00	0.00	0.00	0.00
hTAS2R46/strychnine	0.33	0.60	0.09	0.50	0.09	0.50
**Test statistics**	**HADDOCK**		**Auto Dock Vina**		**Glide**	
**Human odorant receptor/agonist complex**	**REC**	**PREC**	**REC**	**PREC**	**REC**	**PREC**
hOR1A1/(*R*)-(−)-carvone	0.00	0.00	0.00	0.00	0.00	0.00
hOR1A1/(*S*)-(+)-carvone	0.00	0.00	0.29	0.67	0.00	0.00
hOR1A1/citronellol	0.00	0.00	0.00	0.00	0.00	0.00
hOR2AG1/amylbutyrate	0.20	1.00	0.40	1.00	0.25	0.50
hOR2M3/3-mercapto-2-methyl-pentan-1-ol	0.25	1.00	0.00	0.00	0.50	1.00
hOR7D4/androstadienone	0.16	1.00	0.16	1.00	0.17	1.00
hOR7D4/androstenone	0.16	1.00	0.16	1.00	0.17	1.00
**Test statistics**	**MM/CG**					
**Human bitter taste receptor/agonist complex**	**REC**	**PREC**				
hTAS2R38/phenylthiocarbamide	1.00	0.75				
hTAS2R38/propylthiouracil	1.00	1.00				
hTAS2R46/strychnine	1.00	1.00				

*The two test statistical metrics used here are recall (REC) and precision (PREC). Residues below the canonical binding site in class A GPCRs (Venkatakrishnan et al., 2013) have not been taken into account for the test statistics calculation, as they are expected not to be involved in ligand binding*.

The three docking programs used in this study [HADDOCK (Dominguez et al., 2003), AutoDock Vina (Trott and Olson, 2010) and Glide (Friesner et al., 2004)] give similar predictions for some of the complexes analyzed. The predictions for hOR7D4 in complex with androstenone and androstadienone show high precision for all three docking programs, but low recall (Figure 1, red circles). In contrast, the predictions for the hTAS2R38/PROP and PTC complexes, as well as the hTAS2R43/IMNB, hOR1A1/citronellol and hOR1A1/(*R*)-(–)-carvone complexes, show all recall and precision equal to zero (Figure 1, dark blue circles). Finally, the predictions for hTAS2R10 in complex with denatonium and strychnine, as well as those for the hTAS2R16 in complex with arbutin or with phenyl-β-D-glucopyranoside and the hTAS2R46/strychnine complex (Figure 1, cyan circles) feature intermediate values of recall and precision for all three docking programs.

For the other hChem-GPCR/agonist complexes, the results are not uniform among the three docking programs (for a detailed description, see Sections 2 and 3 in the Supplementary Information). In particular, the prediction for the hTAS2R1/dextromethorphan complex has precision 1.0 for HADDOCK, but recall 1.0 for AutoDock Vina and both zero recall and zero precision for Glide. Instead, that for the hOR2M3/3-mercapto-2-methyl-1-penthanol complex shows precision 1.0 for both HADDOCK and Glide, while for AutoDock Vina has both zero recall and precision. Given this high variability among complexes and docking programs, no particular trends can be drawn. Furthermore, the differences in performance may be due not only to the limitations of the docking algorithms, but also of the homology models. Nonetheless, it is noteworthy that even the group with the best docking performance (red circles in Figure 1) is able to recover only a few of the experimentally characterized binding residues.

Overall, the predictive power of the bioinformatic approach is low; only 36% (or less) of the residues were predicted correctly (see Methods), regardless of the docking program used (36, 35, and 33% for HADDOCK, AutoDock Vina and Glide, respectively). Of course, one cannot exclude that some of the residues are exclusively involved in activation and not in the binding. Nonetheless, these are expected to be very few, as all of the mutations considered here are localized closely to the putative binding region, as known from the crystal structures of class A GPCRs (Venkatakrishnan et al., 2013). Hence, we expect that the difficulties in interpreting the experimental data are not going to change the main conclusion of this analysis, namely that the bioinformatics/docking procedure is able to recover only a few of the experimentally characterized binding residues. The low predictive power of the bioinformatics approaches, may be caused, at least in part, by the low resolution of the homology modeling techniques when the sequence identity between the target and the template is low, as it is the case for hChem-GPCRs. In addition, the limited sampling of standard docking techniques might be insufficient to exhaustively explore the conformational space of the ligand bound in the binding site (see also Supplementary Information Section 1.1). Thus, the bioinformatics-based procedure calls for refinement to improve the results. An insight into this issue is offered in the next section.

### Molecular dynamics-based refinement of binding predictions

While the complete molecular simulations of all the complexes in Table 2 is beyond the scope of the present paper, it is interesting to discuss the reliability of simulations on a few specific cases, which have been already studied in our group (see Methods section). We focus on our own studies carried out using the so-called hybrid Molecular Mechanics/Coarse-Grained (MM/CG) molecular dynamics simulation approach. The method, developed in our group (Neri et al., 2005, 2008; Leguebe et al., 2012; Giorgetti and Carloni, 2014; Musiani et al., 2014, 2015), focuses the computational effort in the binding site, where ligand, solvent and protein are treated with an atomistic force field, whereas the rest of the protein is described using a coarse-grained representation and the presence of the membrane is modeled by introducing appropriately designed wall potentials (Leguebe et al., 2012). Hence, the approach includes hydration at the binding site, as well as temperature fluctuations and protein flexibility, increasing the sampling of the conformational space of the ligand binding site.

We calculate the recall and the precision values for the MM/CG complexes previously studied by our group [TAS2R38/PTC, TAS2R38/PROP Marchiori et al., 2013 and TAS2R46/strychnine Sandal et al., 2015] and the results turn out to be highly encouraging: both the recall and the precision values are near or equal to one (Figure 1A and Section 4 of the Supplementary Information). In particular, the number of FN decreases to 0 for all three complexes, improving the recall compared to the bioinformatics predictions (see Table 3). In addition, zero FPs are present for the hTAS2R46/strychnine and hTAS2R38/propylthiouracil complexes and only one for the hTAS2R38/phenylthiocarbamide complex, increasing the precision. Hence, the MM/CG simulations are able to dramatically improve the prediction results by capturing the majority of the residues crucial for ligand-receptor interaction, without introducing any significant bias, at least for the hChem-GPCR/agonist cases studied so far. Indeed, based on the predictive power of the method, several residues playing a role for ligand binding were identified and were subsequently confirmed by performing additional mutagenesis and functional experiments (Marchiori et al., 2013; Sandal et al., 2015). Systematic MM/CG simulations and extensive comparison with experiments are required to establish the predictive power of the method across all hChem-GPCRs.

### Receptor activation predictions

Although it cannot be excluded completely that they could also be involved in ligand binding, several residues have been previously suggested to play a role for activation in hTAS2Rs (Biarnes et al., 2010; Pydi et al., 2014a). These are residues whose mutation causes changes in receptor's response, from abolishing activation to constitutive activation (see Supplementary Table 3). Here, we show that our bioinformatics analysis provides further support to some of these findings. Namely, we can distinguish three different groups of residues (hereafter indicated using the class A GPCR generic numbering Isberg et al., 2015).

The first group, proposed to be involved in hTAS2R1 activation, includes N24 and R55 (positions 1.50 and 2.50) (Singh et al., 2011a). These residues are highly conserved across hTAS2Rs (92 and 96% respectively, see Supplementary Table 3). Moreover, we notice here that these two positions are also conserved in human class A GPCRs (98 and 87%) and have been shown to play a role for activation across class A hGPCRs, based on mutagenesis data (see references Fenalti et al., 2014; Labadi et al., 2015 and Supplementary Table 4) and structural analyses (Venkatakrishnan et al., 2013; Tehan et al., 2014). Therefore, this may further support the claimed role of positions 1.50 and 2.50 for hTAS2Rs activation. Nonetheless, the chemical nature of residue 2.50 changes dramatically, from a positively charged Arg in hTAS2Rs to a negatively charged Asp in class A GPCRs. Hence, we suggest here distinct activation mechanisms on passing from bitter taste receptors to class A hGPCRs, yet converging at the same positions.

Next, we consider position 7.52, which has been suggested to play a role in activation for hTAS2R38 (Biarnes et al., 2010). A branched aliphatic residue (V, L or I) is present at this position in 92% hTAS2Rs (Supplementary Table 3). This position has never been proposed to be involved in an interaction network that changes upon activation in any class A GPCR. Therefore, to blindly investigate if this is the case, we used a pool of structures of human class A GPCRs (see Table 4 and reference Venkatakrishnan et al., 2016) for which both active and inactive structures are available and carried out a graph-based structural analysis with the aim of identifying pairs of highly conserved residues that change intramolecular interactions upon activation (Tehan et al., 2014; Venkatakrishnan et al., 2016). This analysis not only confirms, as expected, all of the previously known residues important for class A GPCR activation, including positions 1.50 and 2.50 (Fenalti et al., 2014; Labadi et al., 2015), but also shows that (i) the hydrophobic nature of residue 7.52 is conserved across human class A GPCRs (Supplementary Table 5), and (ii) this residue does change its interactions upon activation (Supplementary Figure 5). This observation is in agreement with previous experimental data showing that mutations at this position modify the receptor activity in class A GPCRs (see Supplementary Table 4). Therefore, our analysis not only confirms that position 7.52 is important for activation in hTAS2Rs, but also suggests for the first time, from a structural point of view, that this position is actively involved in a network of residues that changes upon activation in class A GPCRs.

**Table 4:** Active/inactive pairs of mammalian class A GPCR crystal structures used for the graph-based structural analysis.

**Class A GPCR**	**Active state**	**Inactive state**	**Species**
β2-adrenergic receptor	2RH1 (2.40)	3SN6 (3.20)	human
M2 muscarinic receptor	3UON (3.00)	4MQS (3.50)	human
adenosine A2A receptor	3EML (2.60)	5G53 (3.40)	human
rhodopsin	1GZM (2.65)	3PQR (2.85)	bovine
μ-opioid receptor	4DKL (2.80)	5C1M (2.10)	murine

*The corresponding PDB codes are listed, together with the crystallographic resolution (between parentheses, in Å)*.

The final group of residues proposed to be involved in activation are I27 in hTAS2R1 (position 1.53) (Singh et al., 2011a), as well as S285 and H214 (positions 7.50 and 5.63) and three residues in the intracellular loop ICL3 (Q216, V234, M237) in hTAS2R4 (Pydi et al., 2012, 2014a). Some of these positions (1.53, 5.63 and 7.50) are highly or fairly well conserved across hTAS2Rs (96, 96, and 68%, respectively). Interestingly, position 7.50 bears either a Ser (68%) or a Pro (28%) in hTAS2Rs, while, in human class A GPCRs, Pro is highly conserved (95%). This position belongs to the conserved TM7 motif NPXXY that is essential for class A GPCRs' activation (Fritze et al., 2003; Audet and Bouvier, 2012; Trzaskowski et al., 2012), but there are no experimental data available for this residue. In the case of ICL3 residues, they do not present high conservation values and a role in activation for these positions in human class A GPCRs has not been suggested so far (and does not emerge from our analysis). This is probably due to their intracellular location in a highly variable region and their likely participation in G-protein binding (Pydi et al., 2014a; Venkatakrishnan et al., 2016) and Gα-subunit selectivity (Flock et al., 2017).

## Conclusions

Structural predictions of human GPCRs are a challenge for computational biologists (Michino et al., 2009; Katritch et al., 2010; Kufareva et al., 2011, 2014; Cavasotto and Palomba, 2015). Integration of experimental and computational information is fundamental to understand ligand binding to these proteins (Thomas et al., 2014; Munk et al., 2016), and in particular to hChem-GPCRs (Charlier et al., 2013; Di Pizio and Niv, 2014; Suku et al., 2017). The reliability of the structural predictions must be validated not only by comparison against previously published experimental data, but also by performing additional site-directed mutagenesis and functional experiments.

Here, we have presented a systematic structural bioinformatics study of all human bitter taste and odorant receptors that feature available experimental data. To the best of our knowledge, this is the first time that such comprehensive study has been undertaken. State-of-the-art bioinformatics approaches, combined with docking algorithms, show clear limitations in the structural predictions of ligand binding determinants. Indeed, several of the residues experimentally shown to be important for ligand binding could not be identified (i.e., low recall), and residues actually not involved in ligand interaction were suggested as so (i.e., low precision) (see Figure 1). These shortcomings are probably due to a variety of factors, including the low sequence identity between the class A GPCR templates and the hChem-GPCR targets, as well as the limited sampling of the docking algorithms. Similarly, previous studies on GPCR/ligand complexes have suggested that the sequence identity lower threshold for accurate prediction of binding modes is between 30% (Beuming and Sherman, 2012) and 40% (Kufareva et al., 2011). As mentioned in the Introduction, a possible way to overcome, at least in part, these limitations is the refinement of the predictions using advanced docking methods or molecular dynamics simulations able to better sample the conformational space (Gelis et al., 2012; Lai and Crasto, 2012; Charlier et al., 2013; Lai et al., 2014). As an example from our own lab, we have shown that the Molecular Mechanics/Coarse Grained (MM/CG) approach developed in our group does improve the quality of the predictions for the three hChem-GPCR/ligand complexes studied so far (Marchiori et al., 2013; Sandal et al., 2015). Similar considerations were suggested for GPCR/ligand complexes in general by Cavasotto and Palomba (2015). Upon reviewing several predictions of the GPCR Dock experiments (Michino et al., 2009; Kufareva et al., 2011, 2014), they concluded that homology modeling combined with docking can be considered just as an initial step in the characterization of ligand-receptor interactions, and that the bioinformatics-based predictions can benefit of refinement with molecular dynamics.

Furthermore, our bioinformatics analysis supports previous claims that residues in positions 1.50, 2.50, and 7.52 might be involved (at least in part) in the activation of hTAS2Rs. Hence, despite the probable differences in the activation mechanisms, some of the activation-related features could be shared between hChem-GPCRs and other class A human GPCRs^[8]^.

## Methods

### Homology modeling

The structures of the human chemosensory receptors (hChem-GPCRs, Table 2 and Supplementary Table 6) were predicted using our GOMoDo webserver (Sandal et al., 2013), following the protocol in references (Marchiori et al., 2013; Sandal et al., 2015).

First, we downloaded all of the available sequences for hTAS2Rs (25) and hORs (464) from the Pfam database (Bateman et al., 2004). The number of hTAS2Rs is established (Meyerhof et al., 2010). In contrast, different numbers have been proposed for hORs (Malnic et al., 2004; Young et al., 2008). This required great care in selecting the hORs used for the corresponding multiple sequence alignment (MSA). Specifically, before performing the alignment, we removed hOR sequences not manually annotated and reviewed, as well as those corresponding to pseudogenes. In addition, once the MSA is generated (see below), we discarded hOR sequences containing large gaps or lacking highly conserved features (Table 1). Indeed, these sequences correspond most likely to not annotated pseudogenes or to open reading frames wrongly predicted to code for hORs.

The 25 hTAS2R sequences and the remaining 411 hOR sequences are aligned using PROMALS (Pei et al., 2007). The resulting two MSAs (see Supplementary Information Section 1.1) were manually curated in order to ensure the alignment of the common conserved features of each chemosensory receptor family. These include: (i) the X.50 position (Isberg et al., 2015); (ii) the conserved structural motifs (Venkatakrishnan et al., 2013; Pydi et al., 2014a, 2016; de March et al., 2015a; Di Pizio et al., 2016) (see Table 1); and, only for hORs, (iii) the two disulfide bridges present in most (~94%) hORs. These involve sulfur atoms of two cysteines of the extracellular loop ECL2 and sulfur atoms of cysteines in ECL2 and TM3 (Cook et al., 2009; Charlier et al., 2013; Kim and Goddard, 2014).

Then, the MSAs were input into the GOMoDo webserver to generate a Hidden Markov Model (HMM) for each subfamily of hChem-GPCRs. The resulting HMMs of the target hChem-GPCRs were aligned against all the HMMs of the GPCR templates available in the GOMoDo webserver, employing HHsearch 2.0.16 (Soding et al., 2005). The use of profile HMMs is known to improve the target-template alignment when dealing with distant homologs (Soding, 2005), as it is the case with the target hChem-GPCRs. 100 models were generated for each target-template pair (see Supplementary Information Section 1.2), using MODELLER 9v10 (Webb and Sali, 2016). The receptor models were then evaluated relying on MODELLER quality scores (low normalized DOPE and high GA341 values) (Melo et al., 2002; Shen and Sali, 2006).

Among all the templates, the human β2 adrenoceptor (PDB code: 4LDE, resolution: 2.79 Å) was identified as the most suitable one (see also Results section). On one hand, the models carried out with β2 adrenoceptor show better MODELLER quality scores (Melo et al., 2002; Shen and Sali, 2006) for all the hChem-GPCRs object of this study. On the other, this template was solved in a fully active state (Venkatakrishnan et al., 2016), which is expected to be the agonist-bound conformational state.

The template-target alignments were then checked and refined by hand, in order to preserve the conserved features of class A GPCRs (see Table 1). Then, 100 new models based on the manually curated alignment between the target hChem-GPCR and the 4LDE template were regenerated, using a standalone version of the MODELLER 9v10 program (Webb and Sali, 2016), and re-evaluated following the procedure described above. For each receptor, the selection of the model was based on (i) the MODELLER quality scores (Melo et al., 2002; Shen and Sali, 2006), and (ii) preservation of the secondary structure of the TM helices. The chosen model was further considered for docking.

Throughout the manuscript, we use the GPCRdb generic number position (Isberg et al., 2015) (except where specified), which generalizes the Ballesteros-Weinstein numbering (Ballesteros and Weinstein, 1995), to have a coherent numeration of the residues between human chemosensory GPCRs and other class A GPCRs. In particular, we used the GPCRdb numbering scheme of the selected template, the β2 adrenoceptor (Supplementary Table 1).

### Molecular docking

The agonists in Table 2 were docked on the final receptor models using (i) HADDOCK (Dominguez et al., 2003) through the GOMoDO webserver (Sandal et al., 2013) (version 2.1), (ii) AutoDock Vina (Trott and Olson, 2010) through Chimera (Pettersen et al., 2004) (version 1.11.2), and (iii) Glide (Grid-based Ligand Docking with Energetics) (Friesner et al., 2004) through the Schrodinger Suite (version 2017-1). All the dockings presented here are blind, i.e., no experimental data was used to guide them. The ligand structures and parameters were obtained from the ZINC database (Irwin and Shoichet, 2005) and the PRODRG webserver (Schuttelkopf and van Aalten, 2004), respectively.

For the prediction-guided HADDOCK docking, putative binding cavity residues (see Supplementary Table 7) were predicted with fpocket (Le Guilloux et al., 2009), and used as active residues to define ambiguous interaction restraints (AIRs). For each ligand, 1000 random structures were generated through initial rigid docking. Then, the structures were ranked and the best 200 complexes underwent refinement with both ligand and receptor treated as flexible (first by simulated annealing, and then by refinement in explicit water). The resulting receptor-agonist complexes were clustered using an RMSD cutoff of 2.0 Å and the complex of the most populated cluster with the lowest energy was chosen for further analysis.

For AutoDock Vina docking, a grid which covers all the fpocket predicted residues was created and the ligands were docked inside the grid. Docking was performed with default parameters (Trott and Olson, 2010) and considering the receptor as rigid. The best complex, i.e., the one with the lowest energy scoring function, was chosen for further analysis.

Finally, for the Glide docking, the grid was built using the same criterium as for AutoDock Vina. 50 binding poses have been produced and ranked according to the Emodel score, a score well-suited for comparing different binding poses of the same ligand (Friesner et al., 2004). The binding pose with the lowest Emodel has been selected.

### Statistical analysis

**Figure 2:**
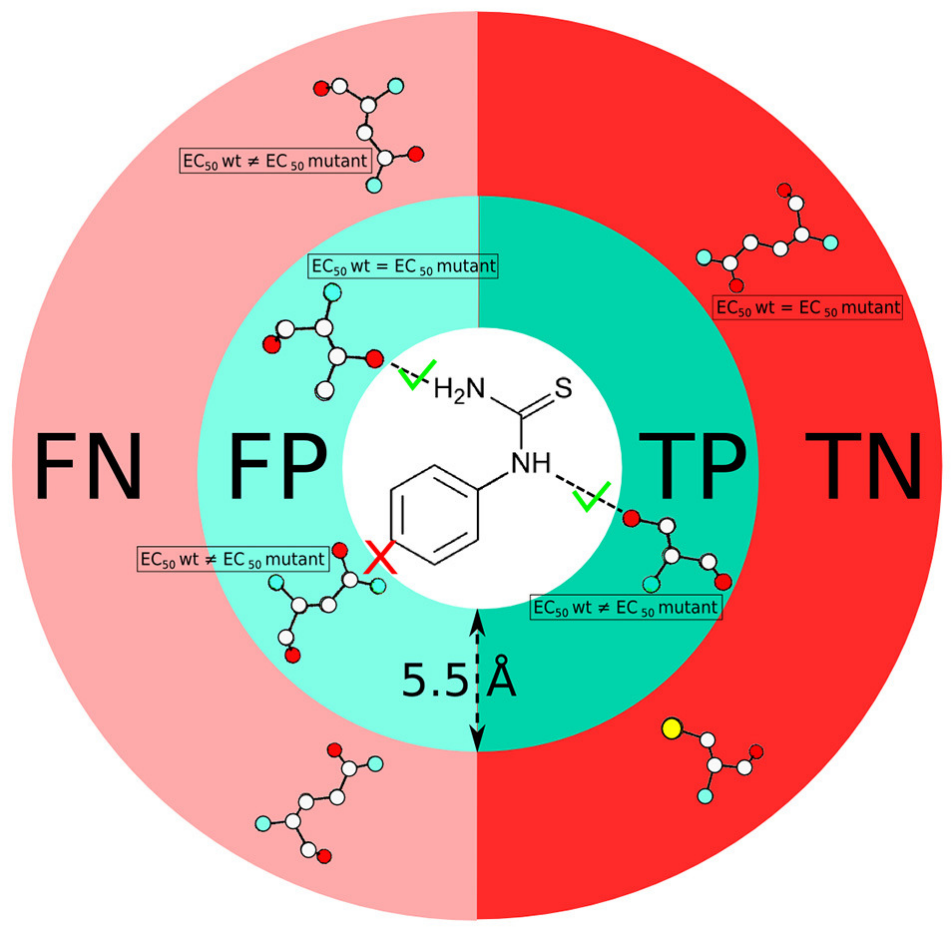
Scheme showing the definition of true positive (TP), false positive (FP), true negative (TN) and false negative (FN) residues used in this study. Comparison of predicted residues with experimental data (EC_50_ values) is performed on the basis of both a distance cut-off (5.5 Å) and a chemical definition (i.e., presence or absence of a canonical protein/ligand interaction).

The residues involved in protein-ligand interactions were identified based on two criteria. As a first step, we use a distance threshold, in which an atomic contact between protein and ligand is considered to be present when their distance is below 5.5 Å (i.e., the sum of the van der Waals carbon radii plus the water molecule diameter (Lee and Richards, 1971; Bohacek and McMartin, 1992; Graziano, 1998). In the second step, we apply a chemical definition, to keep only those contacts that do correspond to classical chemical interactions (i.e., hydrogen bonds, salt bridges, stacking or hydrophobic contacts, etc.) upon visual inspection. Using these two criteria, we analyze the number of true positives (TP), false positives (FP), true negatives (TN) and false negatives (FN) for all ligand-receptor complexes (see Figure 2), by comparison with the experimental data (Supplementary Table 1). Only mutants located in the top half of the receptor are taken into account because this is the location of the canonical orthosteric binding site of class A GPCRs (Venkatakrishnan et al., 2013). Using the aforementioned test outcomes, we calculate the corresponding values of precision,

$$\begin{array}{l} \text{PREC}=\text{TP}/\left(\text{TP}+\text{FP}\right) \end{array}$$

and recall,

$$\begin{array}{l} \text{REC}=\text{TP}/\left(\text{TP}+\text{FN}\right) \end{array}$$

in order to assess the reliability of our docking results. These standard statistical parameters are usually employed for method performance assessment (Raghavan et al., 1989; Manning and Schütze, 1999; Davis and Goadrich, 2006; Saito and Rehmsmeier, 2015). Here, the precision is given by the ratio between the experimentally characterized binding residues that the bioinformatics approach is able to capture (TP) and other residues that docking wrongly considers as important in ligand-receptor interaction (FP). On the other hand, the recall is the ratio between TP and experimentally characterized binding residues that the bioinformatics approach is not able to capture (FN). Both precision and recall are normalized and thus values equal to one suggest optimum performance of the method. In addition, in order to evaluate the predictive power of the bioinformatics approach, we calculate the following percentage:

$$\begin{array}{l} \begin{array}{l} {\left(\text{TP}+\text{TN}\right)}^{*}100/(\text{total number of experimental data}\\ \text{in the top half of the receptor}) \end{array} \end{array}$$

which is not aimed at evaluating the performance of the individual docking programs, but the combination of homology modeling and docking.

### Multiscale molecular dynamics simulations

As written above, the simulations analyzed here correspond to our previous works on hTAS2R38 in complex with two of its agonists (PROP and PTC) (Marchiori et al., 2013) and hTAS2R46 in complex with strychnine (Sandal et al., 2015). These simulations were carried out with a Molecular Mechanics/Coarse-Grained (MM/CG) method developed in our group. In this approach, the MM part (ligand and surrounding protein residues and water molecules) is treated with the GROMOS96 atomistic force field (Scott et al., 1999), whereas the CG part (the rest of the protein, including only the Cα atoms) is described using a Go-like model (Go and Abe, 1981). The two regions are connected at the interface by using a coupling scheme (Neri et al., 2005, 2008). The presence of the membrane is mimicked by introducing five repulsive walls (Leguebe et al., 2012; Giorgetti and Carloni, 2014; Musiani et al., 2014, 2015). For each hTAS2R38 complex (PROP or PTC), two replicas (with different velocities) were run for 0.6 μs each (Marchiori et al., 2013). For the hTAS2R46/strychnine complex, three replicas were run for 1 μs each (Sandal et al., 2015).

### Receptor activation predictions

The sequences of all human class A GPCRs (698) were downloaded from the Pfam database and aligned with PROMALS (Pei et al., 2007). Conserved motifs were identified using in-house scripts written in Python.

The structural analysis was performed on the X-ray structures of human class A GPCRs crystallized both in the active and inactive states, listed in Table 4. We generated a contact map for all independent atomic distances (excluding backbone atoms) and then defined a contact between a pair of residues as formed when the distance between any two atoms of the residue pair is shorter than the sum of their van der Waals radii, as defined in reference (Venkatakrishnan et al., 2016). For completeness and in line with references (Venkatakrishnan et al., 2013; Tehan et al., 2014), this analysis was also performed on the other two mammalian receptors solved in both active and inactive states, i.e., bovine rhodopsin (1GZM/3PQR) and murine μ-opioid receptor (4DKL/5C1M), confirming the results obtained for the human structures (see Results section).

## Author contributions

Wrote the paper: FF, ES, MAP, AG, and PC; analyzed the data: FF, ES, MAP, AG, and PC; designed the experiments: FF, ES, MAP, AG, SC, and PC; conducted the experiments: FF, ES, MAP, and AG; provided data that started the project: SC. All authors read and approved the final manuscript.

### Conflict of interest statement

The authors declare that the research was conducted in the absence of any commercial or financial relationships that could be construed as a potential conflict of interest.
