# Supplementary material for: Agonist Binding to Chemosensory Receptors: A Systematic Bioinformatics Analysis
Source: Front Mol Biosci. 2017 Sep 6;4:63. doi: 10.3389/fmolb.2017.00063 (PMC5592726; doi:10.3389/fmolb.2017.00063)
Supplement: Supplementary file 1 [file DataSheet1.pdf]

Agonist binding to chemosensory receptors: a systematic bioinformatics analysis

Supplementary Information

Part 1

Fabrizio Fierro[1,†], Eda Suku[2,†], Mercedes Alfonso-Prieto[1,3,*], Alejandro Giorgetti[1,2,*], Sven Cichon[4,5,6], Paolo Carloni[1,7,8]

[1] *Computational Biomedicine, Institute for Advanced Simulation IAS-5 and Institute of Neuroscience and Medicine INM-9, Forschungszentrum Jülich, 52425 Jülich, Germany.*

[2] *Department of Biotechnology, University of Verona, Ca' Vignal 1, Strada le Grazie 15, 37134 Verona, Italy.*

[3] *Cécile and Oskar Vogt Institute for Brain Research, Medical Faculty, Heinrich Heine University Düsseldorf, Merowingerplatz 1a, 40225 Düsseldorf, Germany.*

[4] *Institute of Neuroscience and Medicine INM-1, Forschungszentrum Jülich, 52425 Jülich, Germany.*

[5] *Institute for Human Genetics, Department of Genomics, Life&Brain Center, University of Bonn, Sigmund-Freud-Straße 25, 53127 Bonn, Germany.*

[6] *Division of Medical Genetics, Department of Biomedicine, University of Basel, Switzerland.*

[7] *Department of Physics, Rheinisch-Westfälische Technische Hochschule Aachen, 52062 Aachen, Germany.*

[8] *VNU Key Laboratory "Multiscale Simulation of Complex Systems", VNU University of Science, Vietnam National University, Hanoi, Viet Nam.*

[†] *These authors have contributed equally to this work.*

**** Correspondence:***

Mercedes Alfonso-Prieto, m.alfonso-prieto@fz-juelich.de

Alejandro Giorgetti, a.giorgetti@fz-juelich.de

Section 1: Homology Modeling

Section 1.1: Limitations of the bioinformatics models. Homology modeling is able to automatically predict reliable three-dimensional structures when the sequence identity (SI) between target sequence and possible available templates is over 30% (Chothia and Lesk, 1986; Baker and Sali, 2001; Eramian et al., 2008; Piccoli et al., 2013). However, the SI between hORs/hTAS2Rs and the possible GPCRs templates is lower (13-20%), and thus the side chains of the model's residues are likely to be wrong. Since the orientation of the side chains is key for protein-ligand interactions, the docking results are expected to be affected as well. In this regard, previous studies on GPCR/ligand complexes have suggested that the sequence identity lower threshold for accurate prediction of ligand poses is between 30% (Beuming and Sherman, 2012) and 40% (Kufareva et al., 2011). Moreover, docking algorithms do not take fully into account receptor flexibility and hydration (Katritch et al., 2010; Spyrakis et al., 2011; Spyrakis and Cavasotto, 2015), which is crucial for ligand binding and receptor activation in GPCRs (Angel et al., 2009; Nygaard et al., 2010; Latorraca et al., 2017). Furthermore, docking takes into account only a rough estimation of the binding affinity or the binding energy, which might be misleading in the case of hChem-GPCRs. The promiscuity of hORs and hTAS2Rs has been proposed to rely on a trade-off between an increased ligand receptive range and a reduced ligand affinity, compared to other GPCRs (Lancet et al., 1993; Meyerhof et al., 2010). As a consequence, docking scoring functions may not discriminate well between different tastant or odorant binding poses (Charlier et al., 2013). In addition, in the case of hORs, odorant binding is expected to rely mainly on hydrophobic interactions, which are not directional, and thus may be difficult to describe with traditional scoring functions, as discussed in references (Gelis et al., 2012; Charlier et al., 2013). Finally, it has been pointed out that ligand binding in hChem-GPCRs may not be fully well described using the “static” picture of docking poses, due to the flexibility of odorants and bitter tastants; instead, a “dynamical” interaction pattern should be considered by performing molecular dynamics (MD) simulations (Gelis et al., 2012; Sandal et al., 2015).

Section 1.2: Multiple sequence alignments of hChem-GPCRs.

Supplementary Figure 1. Sequence logos of (a) hTAS2Rs and (b) hORs, based on the multiple sequence alignments (MSAs) of each human chemosensory receptor subfamily. The first N-terminal residues (28 for hTAS2Rs and 32 for hORs) have been omitted for the sake of clarity; the same was done for the last C-terminal positions (9 and 101, respectively). These segments are well outside the seven transmembrane bundle of GPCRs. The full-length MSAs can be obtained from the authors upon request.

(a)

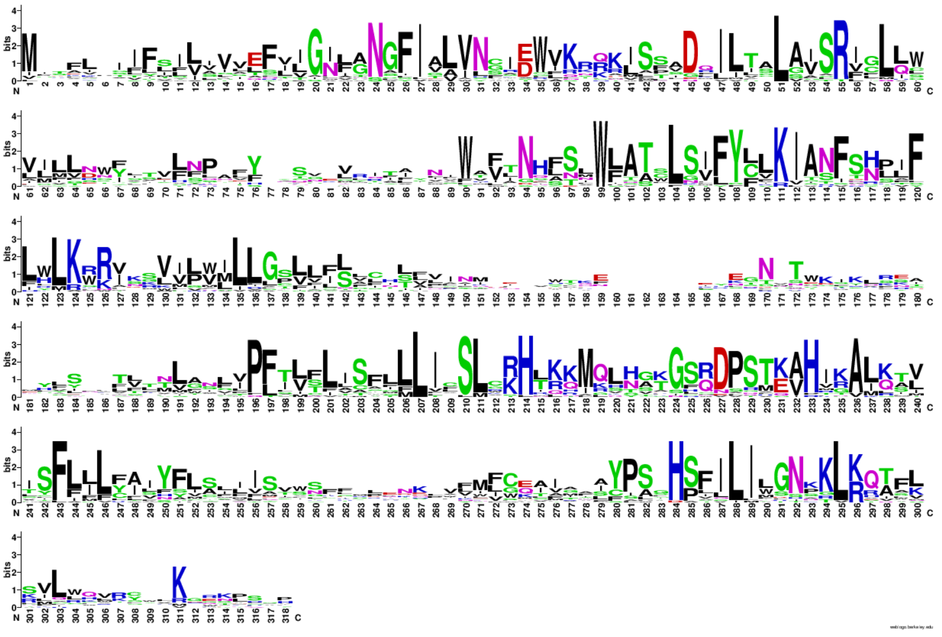

(b)

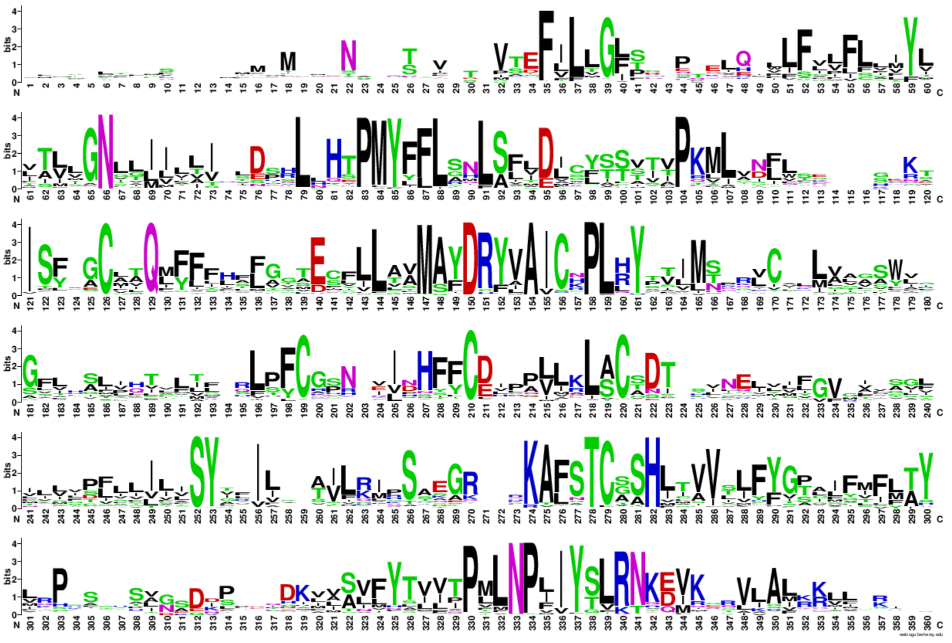

Section 1.3: Target-template pairwise alignments. The HMM-based pairwise alignment between each of the hChem-GPCRs studied in this work and the template (β2-adrenoceptor, PDB code 4LDE) are provided below in PIR format.

```
>P1;tas2r1
sequence:tas2r1: : : : : : : 0.00: 0.00
LESHLIIYFLLAVIQFLLGIFTNGIIVVVNGIDLIKHRKMAPLDLLLSCLAVSRIFLQLFIFYVNVIVI
FF----IEFIMCSANCAILLFINELELWLATWLGVFYCAKVASVRHPLFIWLKMRISKLVPWMILGSLL
YVSMICVFHYAGFMVPYFLRKFFSQNATIQKE-DTLAIQIFSFVAEFSVPLLIFLFAVLLLIFSLGRHT
RQMRNTVAGSRVPGRGAPISALLSILSFLILYFSHCMIKVFLSSLKFH----IRRFIFLFFILVIGIYP
SGHSLILILGNPKLKQNAKKFLLHSKCCQ*
>P1;4lde
structureX:4lde: 1: A: 314: : : :0.00:0.00
-DEVWVVGMGIVMSLIVLAIVFGNVLVITAIAKF---ERLQTVTNYFITSLACADLVMGLAVVPFGAAH
ILTKTWTFGNFWCEFWTSIDVLCVTASIETLCVIAVDRYFAIT---SPF-KYQSLLTKNKARVIILMVW
IVSGLTSFLPIQMHWYRATHQEAINCYAEETCCDFFTNQAYAI-ASSIVSFYVPLVIMVFVYSRVFQEA
KRQLQKID-------KFALKEHKALKTLGIIMGTFTLCWLPFFIVNIVHVIQDNLIRKEVYILLNWIGY
VNSGFNPLIYCR-SPDFRIAFQELLCL-*
```

```
>P1;tas2r4
sequence:sp_Q9NYW5_T2R4_HUMAN_Tas/1-299:   1: : 299: :: : 0.00: 0.00
------MLRLFYFSAIIASVILNFVGIIMNLFITVVNCKTWVKSHRISSSDRILFSLGITRFLMLGLFLV
NTIYFVSSNTE-RSVYLSAFFVLCFMFLDSSSVWFVTLLNILYCVKITNFQHSVFLLLKRNISPKIPRL
LLACVLISAFTTCLYITLSQASPFP-ELVTTRNNTSFNISEGILSLVVSLVLSSSLQFIINVTSASLLI
HSLRRHIQKMQKNATGFWNPQTEAHVGAMKLMVYFLILYIPYSVATLVQYLPFYAGMDMGTKSICLIFA
TLYSPGHSVLIIITHPKLKTTAKKILCFKK*
>P1;4lde_A
structureX:4lde: 1: A: 314: : : :0.00:0.00
TWDAYAADEVWVVGMGIVMSLIVLAIVFGNVLVITAIAK---FERLQTVTNYFITSLACADLVMGLAVV
PFGAAHILTKTWTFGNFWCEFWTSIDVLCVTASIETLCVIAVDRYFAIT---SPFKYQSLLTKNKARVI
ILMVWIVSGLTSFLPIQMHWYRATHQEAINCYAEETCCDFFTNQAYAIASSIVSFYVPLVIMVFVYSRV
FQEAKRQLQK------FALKEHKALKTLGIIMGTFTLCWLPFFIVNIVHVIQDN-LIRKEVYILLNWIG
YVNSGFNPLIYC-RSPDFRIAFQELLCL--*
```

```
>P1;tas2r10
sequence:tas2r10: : : : : : : 0.00: 0.00
MLRVVEGIFIFVVVSESVFGVLGNGFIGLVNCIDCAKN-KLSTIGFILTGLAISRIFLIWIIITDGFIQ
IFSPNYASGNLIEYISYFWVIGNQSSMWFATSLSIFYFLKIANFSNYIFLWLKSRTNMVLPFMIVFLLI
SSLLNFYIAKILNDYK-TKNDTVWDLNMYKSEYFIKQILLNLGVIFFFTLSLITCIFLIISLWRHNRQM
QSNVTGLRDSNTEAHVKAMKVLISFIILFILYFIGMAIEISCFTVRENKLLLMFGMTTTAIYPWGHSFI
LILGNSKLKQASLRVLQQLKC*
>P1;4lde
structureX:4lde: 1: A: 314: : : :0.00:0.00
-DEVWVVGMGIVMSLIVLAIVFGNVLVITAIAK---FERLQTVTNYFITSLACADLVMGLAVVPFGAAH
ILTTWTFGNFWCEFWTSIDVLCVTASIETLCVIAVDRYFAITSPFKYQSLLTKNKARVIILMVWIVSGL
TSFLQMHWYRATHQEAINCYAEETCCDFFTNQAYAIASSIVSFYVPLVIMVFVYSRVFQEAKRQLQ---
K----AKEHKALKTLGIIMGTFTLCWLPFFIVNIVHVIQDN-LIRKEVYILLNWIGYVNSGFNPLIYC-R
SPDFRIAFQELLCL------*
```

>P1;**tas2r16**
sequence:sp_Q9NYV7_T2R16_HUMAN_Tas/1-291:   1: : 291: :: : 0.00: 0.00
MIPIQLTVFFMIIYVLESLTIIVQSSLIVAVLGREWLQVRRLMPVDMILISLGISRFCLQWASMLNNFC
SYFNLN-----YVLCNLTITWEFFNILTFWLNSLLTVFYCIKVSSFTHHIFLWLRWRILRLFPWILLGSL
MITCVTIIPSAIGNYIQIQLLTMEHLPRNSTVTDKLENFHQYQFQAHTVALVIPFILFLASTIFLMASL
TKQIQHH---STGHCNPSMKARFTALRSLAVLFIVFTSYFLTILITIIGTLFDKRCWLWVWEAFVYAFI
LMHSTSLMLSSPTLKRILKGKC*
>P1;4lde
structureX:4lde: 1: A: 314: : : :0.00:0.00
--DEVWVVGMGIVMSLIVLAIVFGNVLVITAIAK---FERLQTVTNYFITSLACADLVMGLAVVPFGAAH
ILTKTWTFGNFWCEFWTSIDVLCVTASIETLCVIAVDRYFAITSPFKYQSLLTKNKARVIILMVWIVSG
LTSFLPIQMHWYRATHQEA-----INCYAEETCCDFFTNQAYAIASSIVSFYVPLVIMVFVYSRVFQEAK
RQLQK-------FALKEHKALKTLGIIMGTFTLCWLPFFIVNIVHVIQDNLIRKEVYILLNWIGYVNSGF
NPLIYC-RSPDFRIAFQELL*

>P1;**tas2r30**
sequence:sp_P59541_T2R30_HUMAN_Tas/1-319:   1: : 319: :: : 0.00: 0.00
LPIIFSILIVVIFVIGNFANGFIALVNSIEWVKRQKISFVDQILTALAVSRVGLLWVLLLHWYATQLNP
AF-YSVEVRITAYNVWAVTNHFSSWLATSLSMFYLLRIANFSNLIFLRIKRRVKSVVLVILLGPLLFLV
CHLFVINMDETVWTKE--YEGNVTWKIKLRSAMYHSNMTLTMLANFVPLTLTLISFLLLICSLCKHLKK
MQLHGKGSQDPSTKVHIKALQTVTSFLLLCAIYFLSMIISVCNFGRLEKQPVFMFCQAIIFSYPSTHPF
ILILGNKKLKQIFLSVLRH*
>P1;4lde
structureX:4lde: 1: A: 314: : : :0.00:0.00
WVVGMGIVMSLIVLAIVFGNVLVITAIAK---FERLQTVTNYFITSLACADLVMGLAVVPFGAAHILTK
TWTFGNFWCEFWTSIDVLCVTASIETLCVIAVDRYFAIT---SPFKYQSLLTKNKARVIILMVWIVSGL
TSFLPIQMHWYRATHQEAINCYAEETCCDFFTNQAYAIASSIVSFYVPLVIMVFVYSRVFQEAKRQLQK
-------FALKEHKALKTLGIIMGTFTLCWLPFFIVNIVHVIQDN-LIRKEVYILLNWIGYVNSGFNPLI
YC-RSPDFRIAFQELLCL*

>P1**;tas2r31**
sequence:tas2r31: : : : : : : 0.00: 0.00
TTFIPIIFSSVVVVLFVIGNFANGFIALVNSIERVKRQKISFADQILTALAVSRVGLLWVLLLNWYSTV
FN--PAFYSVEVRTTAYNVWAVTGHFSNWLATSLSIFYLLKIANFSNLIFLHLKRRVKSVILVMLLGPL
LFLACQ---LFVINMKEIVRTKEYEGNLTWKIKLRSAVY--LSDATVTTLGNLVPFTLTLLCFLLLICSL
CKHLKKMQLHGKGSQDPSTKVHIKALQTVIFFLL-LCAVYFLSIMISVWSFGSLE--NKPVFMFCKAIR
FSYPSIHPFILIWGNKKLKQTFLSVLRQVRYWVK*
>P1;4lde
structureX:4lde: 1: A: 314: : : :0.00:0.00
-DEVWVVGMGIVMSLIVLAIVFGNVLVITAIAK---FERLQTVTNYFITSLACADLVMGLAVVPFGAAH
ILTKTWTFGNFWCEFWTS-IDVLCVTASIETLCVIAVDRYFAITSPF-KYQSLLTKNKARV--IILMV--
-WIVSGLTSFLPIQMHWYRATHQEAINCYAEETCCDFFTNQAYAIASSIVSFYVPLVIMVFVYSRVFQE
AKRQLQKIDK--------FALKEHKALKTLGIIMGTFTLCWLPFFIVNIVHVIQDNLIRKEVYILLNWIG
YVNSGFNPLIYCR-SPDFRIAFQELLCLRRSSL*

>P1;**tas2r38**
sequence:sp_P59533_T2R38_HUMAN_Tas/1-333:   1: : 333: :: : 0.00: 0.00
MLTLTRIRTVSYEVRSTFLFISVLEFAVGFLTNAFVFLVNFWDVVKRQALSNSDCVLLCLSISRLFLHG
LLFLSAIQLTHFQKLSEPLNHSYQAIIMLWMIANQANLWLAACLSLLYCSKLIRFSHTFLICLASWVSR
KISQMLLGIILCSCICTVLCVWCFFSRPHFTVTTVLFMNNNTRLNWQIKDLNLFYSFLFCYLWSVPPFL
LFLVSSGMLTVSLGRHMRTMKVYTRNSRDPSLEAHIKALKSLVSFFCFFVISSCAAFISVPLLILWRDK
IGVMVCVGIMAACPSGHAAILISGNAKLRRAVMTILLWAQSSLK*

>P1;4lde
structureX:4lde: 1: A: 314: : : :0.00:0.00
---TWDAYAADEVWVVGMGIVMSLIVLAIVFGNVLVITAIAK---FERLQTVTNYFITSLACADLVMGLA
VVPFGAAHILTKTWT-FGNFWCEFWTSIDVLCVTASIETLCVIAVDRYFAIT---SPFKYQSLLTKNKA
RVIILMVWIVSGLTSFLPIQMHWYRATHQ-----EAINCYAEETCCDFFTNQAYAIASSIVSFYVPLVIM
VFVYSRVFQEAKRQLQKFA-------LKEHKALKTLGIIMGTFTLCWLPFFIVNIVHVIQDN-LIRKEVY
ILLNWIGYVNSGFNPLIYC-RSPDFRIAFQELLCL------*

>P1;**tas2r43**
sequence::   1: : 302: :: : 0.00: 0.00
MITFLPIIFSSLVVVTFVIGNFANGFIALVNSIEWFKRQKISFADQILTALAVSRVGLLWVLLLNWYST
VLNPAF-NSVEVRTTAYNIWAVINHFSNWLATTLSIFYLLKIANFSNFIFLHLKRRVKSVILVMLLGPL
LFLACHLFVINMNEIVRTKE--FEGNMTWKIKLKSAMYFSNMTVTMVANLVPFTLTLLSFMLLICSLCK
HLKKMQLHGKGSQDPSTKVHIKALQTVISFLLLCAIYFLSIMISVWSFGSLENKPVFMFCKAIRFSYPS
IHPFILIWGNKKLKQTFLSVFWQ*
>P1;4lde
structureX:4lde: 1: A: 314: : : :0.00:0.00
ADEVWVVGMGIVMSLIVLAIVFGNVLVITAIAK---FERLQTVTNYFITSLACADLVMGLAVVPFGAAH
ILTKTWTFGNFWCEFWTSIDVLCVTASIETLCVIAVDRYFAIT---SPFKYQSLLTKNKARVIILMVWI
VSGLTSFLPIQMHWYRATHQEAINCYAEETCCDFFTNQAYAIASSIVSFYVPLVIMVFVYSRVFQEAKR
QLQK-------FALKEHKALKTLGIIMGTFTLCWLPFFIVNIVHVIQDN-LIRKEVYILLNWIGYVNSGF
NPLIYC-RSPDFRIAFQELLCL*

>P1;**tas2r46**
sequence:tas2r46: : : : : : : 0.00: 0.00
MITFLPIIFSILIVVTFVIGNFANGFIALVNSIEWFKRQKISFADQILTALAVSRVGLLWVLVLNWYAT
ELNPAF-NSIEVRITAYNVWAVINHFSNWLATSLSIFYLLKIANFSNLIFLHLKRRVKSVVLVILLGPL
LFLVCHLFVINMNQIIWTKE--YEGNMTWKIKLRSAMYLSNTTVTILANLVPFTLTLISFLLLICSLCK
HLKKMQLHGKGSQDPSMKVHIKALQTVTSFLLLCAIYFLSIIMSVWSFESLENKPVFMFCEAIAFSYPS
THPFILIWGNKKLKQTFLSVLWHVRYWVK*
>P1;4lde
structureX:4lde: 1: A: 314: : : :0.00:0.00
ADEVWVVGMGIVMSLIVLAIVFGNVLVITAIAK---FERLQTVTNYFITSLACADLVMGLAVVPFGAAH
ILTKTWTFGNFWCEFWTSIDVLCVTASIETLCVIAVDRYFAIT---SPFKYQSLLTKNKARVIILMVWI
VSGLTSFLPIQMHWYRATHQEAINCYAEETCCDFFTNQAYAIASSIVSFYVPLVIMVFVYSRVFQEAKR
QLQKIDK----FALKEHKALKTLGIIMGTFTLCWLPFFIVNIVHVIQDN-LIRKEVYILLNWIGYVNSGF
NPLIYC-RSPDFRIAFQELLCLRRSSLK*

>P1;**or1a1**
sequence:Q9P1Q5: : : : : : : 0.00: 0.00
QQEQEDFFYILFLFIYPITLIGNLLIVLAICSDVRLHNPMYFLLANLSLVDIFFSSVTIPKMLANHLLG
SKSISFGGCLTQMYFMIALGNTDSYILAAMAYDRAVAISRPLHYTTIMSPRSCIWLIAGSWVIGNANAL
PHTLLTASLSFCGNQEVANFYCDITP-LLKLSCSDIHFHVKMMYLGVGIFSVPLLCIIVSYIRVFSTVF
QV-----PSTKGVLKAFSTCGSHLTVVSLYYGTVMGTYFRPLTNY---SLKDAVITVMYTAVTPMLNPFI
YSLRNRDMKAALRKLFNKRISS*
>P1;4lde
structureX:4lde: 1: A: 314: : : :0.00:0.00
DEVWVVGMGIVMSLIVLAIVFGNVLVITAIAKFERLQTVTNYFITSLACADLVMGLAVVPFGAAHILTK
TWTFGNFWCEFWTSIDVLCVTASIETLCVIAVDRYFAITSPFKYQSLLTKNKARVIILMVWIVSGLTSF
LPIQMHWYRAT----HQEAINC----YAEETCCDFFTNQAYAIASSIVSFYVPLVIMVFVYSRVFQEAKR
QLQ---KFALKEHKALKTLGIIMGTFTLCWLPFFIVNIVHVIQDNLIRKEVYILLNWIGYVNSGFNPLI
YCR-SPDFRIAFQELLCL---*

>P1;**or2ag1**
sequence:OR2AG1: : : : : : : 0.00: 0.00
DSGSPELLCATITILYLLALISNGLLLLAITME---ARLHMPMYLLLGQLSLMDLLFTSVVTPKALADF
LRRENTISFGGCALQMFLALTMGGAEDLLLAFMAYDRYVAICHPLTYMTLMSSRACWLMVATSWILASL
SALIYTVYTMHYPFCRAQEIRHLLCEIPHLLKVACADTSRYELMVYVMGVTFLIPSLAAILASYTQILL
TVLHM-PSNEGRKKALVTCSSHLTVVGMFYGAATFMYVLPSSFHS----TRQDNIISVFYTIVTPALNPL
IYSLRNKEVMRALRRVLGKYMLPAH---*
>P1;4lde
structureX:4lde: 1: A: 314: : : :0.00:0.00
DEVWVVGMGIVMSLIVLAIVFGNVLVITAIAK---FERLQTVTNYFITSLACADLVMGLAVVPFGAAHI
LTKTWTFGNFWCEFWTSIDVLCVTASIETLCVIAVDRYFAITSPFKYQSLLTKNKARVIILMVWIVSGL
TSFLPIQMHWYRAT-H-QEAIN--CYAEET----CCDFFTNQAYAIASSIVSFYVPLVIMVFVYSRVFQE
AKRQLQKFALKEHKALKTLGIIMGTFTLCWLPFFIVNIVHVIQDNLIR-KEVYILLNWIGYVNSGFNPL
IYCR-SPDFRIAFQELLCL---------*

>P1;**or2m3**
sequence:Q8NG83.1_31-342_Olfactory/1-312:   11: : 307: :: : 0.00: 0.00
DFILLGIFNHSPTHTFLFFLVLAIFSVAFMGNSVMVLLIYLDTQLHTPMYLLLSQLSLMDLMLICTTVP
KMAFNYLSGSKSISMAGCATQIFFYTSLLGSECFLLAVMAYDRYTAICHPLRYTNLMSPKICGLMTAFS
WILGSTDGIIDVVATFSFSY------CGSREIAHFFCDFPSLLILSCSDTSIFEKILFICCIVMIVFPVA
IIIASYARVILAVIHM---GSGEGRRKAFTTCSSHLLVVGMYYGAALFMYIRPTSDRS--PTQDKMVSVF
YTILTPMLNPLIYSLRNKEVTRAFMKILGK*
>P1;4lde
structureX:4lde: 1: A: 314: : : :0.00:0.00
TGTWDAYAADEVWVVGMGIVMSLIVLAIVFGNVLVITAIAKFERLQTVTNYFITSLACADLVMGLAVVP
FGAAHILTKTWTFGNFWCEFWTSIDVLCVTASIETLCVIAVDRYFAITSPFKYQSLLTKNKARVIILMV
WIVSGLTSFLPIQMHWYRATHQEAINCYAEET----C---------C-DFFTNQAYAIASSIVSFYVPLVI
MVFVYSRVFQEAKRQLQK-FALKEHKALKTLGIIMGTFTLCWLPFFIVNIVHVIQDNLIRKEVYILLNW
IGYVNSGFNPLIYCR-SPDFRIAFQELLCL*

>P1;**or7d4**
sequence:Q8NG98.1_31-342_Olfactory/1-312:   1: : 286: :: : 0.00: 0.00
ELQPVLFGLFLSMYLVTVLGNLLIIILAVSSDSHLHTPMYFFLSNLSFVDICFISTTVPKMLVSIQARSK
DISYMGCLTQVYFLMMFAGMDTFLLAVMAYDRFVAICHPLHYTVIMNPCLCGLLVLASWFIIFWFSLVH
ILLMKRLTFSTGTEIPHFFCEPAQVLKVACSNTLLNNIVLYVATALLGVFPVAGILFSYSQIVSSLMGM
---SSTKGKYKAFSTCGSHLCVVSLFYGTGLGVYLSSAVTHS--SQSSSTASVMYAMVTPMLNPFIYSLR
NKDVKGALERLLSR*
>P1;4lde
structureX:4lde: 1: A: 314: : : :0.00:0.00
VWVVGMGIVMSLIVLAIVFGNVLVITAIAKFERLQTVTNYFITSLACADLVMGLAVVPFGAAHILTKTW
TFGNFWCEFWTSIDVLCVTASIETLCVIAVDRYFAITSPFKYQSLLTKNKARVIILMVWIVSGLTSFLP
IQMHWYR----ATHQEAINCYAEETCC----DFFTNQAYAIASSIVSFYVPLVIMVFVYSRVFQEAKRQL
QK-FALKEHKALKTLGIIMGTFTLCWLPFFIVNIVHVIQDNLIRKEVYILLNWIGYVNSGFNPLIYCR-
SPDFRIAFQELLCL*

Section 2: Quality of the docking results.

Section 2.1: EC_{50} values interpretation

In principle, mutations affecting agonist binding should affect EC_{50} concentration values, while those affecting activation could affect the overall amplitude of the dose-response curve (Colquhoun, 1998; Strange, 2008). However, any mutation may simultaneously shift the relative population of the spectrum of receptor conformations relative to the wild-type receptor (Changeux and Edelstein, 2011). This leads to conformation-dependent effects, rather than agonist-based effects (Kenakin, 2002). In addition, effects indirectly related to the ligand affinity of the receptor are also possible, such as shaping of the binding cavity (Marchiori et al., 2013; Sandal et al., 2015) and second-shell effects (i.e. residues important to maintain the actual binding residue(s) in the right conformation to interact with the ligand) (Singh et al., 2011, Geithe et al., 2017). As a result, EC_{50} measurements cannot be simply interpreted in terms of binding affinity or activation (Colquhoun, 1998; Strange, 2008; Williams and Hill, 2009; Strange, 2010).

Section 2.2: **Statistical analysis of the docking results**.

Supplementary Table 1. Analysis of the docking data of the hChem-GPCR/agonist complexes predicted by Haddock (Dominguez et al., 2003), Autodock Vina (Trott and Olson, 2010) and Glide (Friesner et al., 2004). In the first column all the hChemGPCR/agonist pairs for which experimental data are available are indicated; the charge of the ligand is shown in parentheses. The second column shows the residues for which mutagenesis data are available. Residues belonging to the bottom half of the receptor, to the N- or C-termini, and to the TM8 have been omitted, as they are well outside the canonical orthosteric binding site of class A GPCRs (Venkatakrishnan et al., 2013) and thus they are expected not to be involved in ligand binding. In the third column, residues are numbered accordingly to the GPCRdb numbering scheme (Isberg et al., 2015) of our template, the β2 adrenoceptor (PDB code: 4LDE). In the fourth column, interpretation of EC_{50} values for the corresponding residues is reported using the following nomenclature: c = change in EC_{50}; nc = no significant change in EC_{50}. In the following columns, for each docking program it is indicated whether the residue is within the 5.5 Å cut-off distance from the ligand (Y=yes; N=no) and the prediction outcome for this residue (TP=true positive, TN=true negative, FP=false positive, FN=false negative, see Figure 3 in the main text), depending on the presence or absence of an actual chemical interaction. For each hChemGPCR/agonist complex, the resulting recall and precision values are shown in the last row (in red for HADDOCK, green for AutoDock Vina and blue for Glide).

	Experimental data			HADDOCK		AutoDock Vina		Glide	
**hChem-GPCR/ agonist complex (ligand charge)**	**Res.**	**Pos.**	**EC_{50}**	**Dist.**	**Pred.**	**Dist.**	**Pred.**	**Dist.**	**Pred.**
**hTAS2R1/ dextromethorphan (+1)**	N66	2.62	c	Y	TP	Y	TP	Y	TP
	E74	ECL1	c	Y	TP	Y	FP	N	FN
	N89	3.36	c	N	FN	Y	FP	N	FN
				**Recall: 0.67 Precision: 1.00**		**Recall: 1.00 Precision: 0.33**		**Recall: 0.00 Precision: 0.00**	
**hTAS2R4/ quinine (+1)**	A90	3.26	c	N	FN	N	FN	N	FN
	F91	3.27	nc	N	TN	N	TN	N	TN
	F92	3.28	nc	Y	FP	Y	FP	Y	FP
	Y155	4.61	c	N	FN	N	FP	N	FN
	N173	ECL2	c	N	FN	N	FN	N	FN
	T174	ECL2	c	N	FN	N	FN	N	FN
	Y258	6.59	c	N	FN	N	FN	N	FN
	K270	7.35	c	Y	TP	Y	TP	Y	TP
				**Recall: 0.17 Precision: 0.50**		**Recall: 0.17 Precision: 0.50**		**Recall: 0.17 Precision: 0.50**	
**hTAS2R10/ denatonium (+1)**	S85	3.29	nc	N	TN	Y	FP	Y	FP
	W88	3.32	nc	Y	FP	Y	FP	Y	FP
	V89	3.33	c	Y	TP	Y	TP	N	FN
	N92	3.36	nc	Y	FP	N	TN	N	TN
	Q93	3.37	nc	Y	FP	N	TN	N	TN
	Q175	5.43	c	N	FN	N	FN	N	FN
	L178	5.46	c	N	FN	N	FN	N	FN
	Y239	6.56	nc	Y	FP	N	TN	N	TN
	M263	7.44	nc	Y	FP	Y	FP	Y	TP
	T266	7.47	c	N	FN	N	FN	N	FN
				**Recall: 0.25 Precision: 0.17**		**Recall: 0.25 Precision: 0.25**		**Recall: 0.20 Precision: 0.33**	

**hTAS2R10/ parthenolide (0)**	S85	3.29	c	N	FN	Y	FP	Y	FP
	W88	3.32	nc	Y	FP	Y	FP	Y	FP
	V89	3.33	nc	Y	FP	N	TN	N	TN
	N92	3.36	nc	Y	FP	N	TN	N	TN
	Q93	3.37	nc	N	TN	N	TN	N	TN
	Q175	5.43	nc	N	TN	N	TN	N	TN
	L178	5.46	c	Y	TP	N	FN	N	FN
	Y239	6.56	nc	Y	FP	N	TN	Y	FP
	M263	7.44	nc	N	TN	Y	FP	Y	FP
	T266	7.47	nc	N	TN	N	TN	N	TN
				**Recall: 0.50 Precision: 0.20**		**Recall: 0.00 Precision: 0.00**		**Recall: 0.00 Precision: 0.00**	
**hTAS2R10/ strychnine (+1)**	S85	3.29	c	Y	FP	Y	TP	Y	FP
	W88	3.32	nc	Y	FP	Y	FP	Y	FP
	V89	3.33	c	Y	TP	Y	TP	Y	TP
	N92	3.36	nc	Y	FP	Y	FP	N	TN
	Q93	3.37	nc	Y	FP	N	TN	N	TN
	Q175	5.43	c	N	FN	N	FN	N	FN
	L178	5.46	c	Y	TP	N	FN	N	FN
	Y239	6.56	nc	N	TN	Y	FP	Y	FP
	M263	7.44	nc	Y	FP	Y	FP	Y	FP
	T266	7.47	nc	N	TN	N	TN	N	TN
				**Recall: 0.67 Precision: 0.29**		**Recall: 0.40 Precision: 0.40**		**Recall: 0.33 Precision: 0.20**	
**hTAS2R16/ arbutin (0)**	E86	3.33	c	Y	TP	Y	TP	Y	TP
	N89	3.36	c	N	FN	N	FN	N	FN
	F93	3.40	c	N	FN	N	FN	N	FN
	Q177	5.39	nc	Y	FP	Y	FP	Y	FP
	H181	5.43	c	N	FN	N	FN	N	FN
	F240	6.52	c	N	FN	N	FN	N	FN
	I243	6.55	c	Y	FP	Y	TP	Y	TP
				**Recall: 0.20 Precision: 0.33**		**Recall: 0.33 Precision: 0.67**		**Recall: 0.33 Precision: 0.67**	
**hTAS2R16/ phenyl-β-D-gluco-pyranoside (0)**	E86	3.33	c	Y	TP	N	FN	Y	FP
	N89	3.36	c	N	FN	N	FN	N	FN
	F93	3.40	c	N	FN	N	FN	N	FN

	Q177	5.39	nc	Y	FP	Y	FP	Y	FP
	H181	5.43	c	N	FN	N	FN	N	FN
	F240	6.52	c	N	FN	N	FN	N	FP
	I243	6.55	c	N	FN	Y	TP	Y	TP
				**Recall: 0.17 Precision: 0.25**		**Recall: 0.17 Precision: 0.5**		**Recall: 0.25 Precision: 0.25**	
**hTAS2R16/ salicin (0)**	E86	3.33	c	Y	TP	Y	TP	N	FN
	N89	3.36	c	N	FN	N	FN	N	FN
	F93	3.40	c	N	FN	N	FN	N	FN
	Q177	5.39	nc	Y	FP	Y	FP	Y	FP
	H181	5.43	c	N	FN	N	FN	N	FN
	F240	6.52	c	N	FN	N	FN	N	FN
	I243	6.55	c	Y	FP	Y	TP	Y	FP
				**Recall: 0.20 Precision: 0.33**		**Recall: 0.33 Precision: 0.67**		**Recall: 0.00 Precision: 0.00**	
**hTAS2R30/ denatonium (+1)**	W88	3.32	c	Y	TP	N	FN	N	FN
	N92	3.36	c	N	FN	N	FN	N	FN
				**Recall: 0.50 Precision: 1.00**		**Recall: 0.00 Precision: 0.00**		**Recall: 0.00 Precision: 0.00**	
**hTAS2R31/ aristolochic acid (-1)**	K265	7.38	c	Y	TP	N	FN	N	FN
	R268	7.41	c	Y	TP	N	FN	N	FN
				**Recall: 1.00 Precision: 0.50**		**Recall: 0.00 Precision: 0.00**		**Recall: 0.00 Precision: 0.00**	
**hTAS2R38/ phenyltiocarbamide (0)**	W99	3.32	nc	Y	FP	N	TN	Y	FP
	N103	3.36	c	N	FN	N	FN	N	FN
	N179	ECL2	nc	N	TN	N	TN	N	TN
	R181	ECL2	nc	N	TN	N	TN	N	TN
	N183	ECL2	nc	N	TN	N	TN	N	TN
	F197	5.42	nc	N	TN	N	TN	N	TN
	W201	5.46	c	N	FN	N	FN	N	FN
	F255	6.47	nc	N	TN	N	TN	N	TN
	F264	6.56	c	N	FN	N	FN	N	FN
				**Recall: 0.00 Precision: 0.00**		**Recall: 0.00 Precision: 0.00**		**Recall: 0.00 Precision: 0.00**	
**hTAS2R38/ propylthiouracil (0)**	W99	3.32	nc	Y	FP	N	TN	Y	FP
	M100	3.33	nc	N	TN	N	TN	N	TN
	N103	3.36	c	N	FN	N	FN	N	FN

	N179	ECL2	nc	N	TN	N	TN	N	TN
	R181	ECL2	nc	N	TN	N	TN	N	TN
	N183	ECL2	nc	N	TN	N	TN	N	TN
	F197	5.42	c	N	FN	N	FN	N	FN
	W201	5.46	c	N	FN	N	FN	N	FN
	F264	6.56	c	N	FN	N	FN	N	FN
				**Recall: 0.00 Precision: 0.00**		**Recall: 0.00 Precision: 0.00**		**Recall: 0.00 Precision: 0.00**	
**hTAS2R43/ IMNB (0)**	N76	ECL1	c	N	FN	N	FN	N	FN
	I87	3.31	nc	N	TN	N	TN	N	TN
	I91	3.35	c	N	FN	N	FN	N	FN
	N92	3.36	c	N	FN	N	FN	N	FN
				**Recall: 0.00 Precision: 0.00**		**Recall: 0.00 Precision: 0.00**		**Recall: 0.00 Precision: 0.00**	
**hTAS2R43/ 6-nitrosaccharin (0)**	N76	ECL1	nc	N	TN	N	TN	N	TN
	I87	3.31	nc	N	TN	N	TN	N	TN
	W88	3.32	c	Y	FP	Y	FP	N	FN
	I91	3.35	nc	N	TN	N	TN	N	TN
	N92	3.36	c	N	FN	N	FN	N	FN
				**Recall: 0.50 Precision: 1.00**		**Recall: 0.00 Precision: 0.00**		**Recall: 0.00 Precision: 0.00**	
**hTAS2R46/ strychnine (+1)**	E70	2.65	c	N	FN	N	FN	N	FN
	L71	ECL1	c	N	FN	N	FN	N	FN
	I82	3.26	c	N	FN	N	FN	N	FN
	N92	3.36	c	Y	TP	N	FN	N	FN
	N150	ECL2	nc	N	TN	N	TN	N	TN
	N161	ECL2	nc	N	TN	Y	FP	Y	FP
	N176	5.39	c	N	FN	N	FN	N	FN
	Y241	6.51	c	Y	TP	N	FN	N	FN
	E253	ECL3	c	N	FN	N	FN	N	FN
	F261	7.35	c	N	FN	Y	TP	Y	TP
	E265	7.39	c	Y	FP	N	FN	N	FN
	A268	7.42	c	Y	TP	N	FN	N	FN
	F269	7.43	c	Y	FP	N	FN	N	FN
				**Recall: 0.33 Precision: 0.60**		**Recall: 0.09 Precision: 0.50**		**Recall: 0.09 Precision: 0.50**	

**hOR1A1/ R-carvone (0)**	A106	3.34	c	N	FN	N	FN	N	FN
	G108	3.36	nc	N	TN	N	TN	N	TN
	N109	3.37	c	N	FN	N	FN	N	FN
	D111	3.39	c	N	FN	N	FN	N	FN
	G152	4.53	nc	N	TN	N	TN	N	TN
	N155	4.56	c	N	FN	N	FN	N	FN
	I205	5.46	nc	N	TN	N	TN	N	TN
	Y250	6.47	c	N	FN	N	FN	N	FN
	Y251	6.48	c	N	FN	N	FN	N	FN
	Y276	7.41	c	N	FN	N	FN	N	FN
	T277	7.42	c	N	FN	N	FN	N	FN
				**Recall: 0.00 Precision: 0.00**		**Recall: 0.00 Precision: 0.00**		**Recall: 0.00 Precision: 0.00**	
**hOR1A1/ S-carvone (0)**	A106	3.34	nc	N	TN	N	TN	N	TN
	G108	3.36	nc	N	TN	N	TN	N	TN
	N109	3.37	c	N	FN	N	FN	N	FN
	D111	3.39	c	N	FN	N	FN	N	FN
	G152	4.53	nc	N	TN	N	TN	N	TN
	N155	4.56	c	N	FN	N	FN	N	FN
	I205	5.46	nc	N	TN	Y	FP	N	TN
	Y250	6.47	c	N	FN	N	FN	N	FN
	Y251	6.48	c	N	FN	Y	TP	N	FN
	Y276	7.41	c	N	FN	N	FN	N	FN
	T277	7.42	c	N	FN	Y	TP	N	FN
				**Recall: 0.00 Precision: 0.00**		**Recall: 0.29 Precision: 0.67**		**Recall: 0.00 Precision: 0.00**	
**hOR1A1/ citronellol (0)**	T99	3.27	nc	N	TN	N	TN	N	TN
	G108	3.36	c	N	FP	N	FP	N	FP
	N109	3.37	c	N	FP	N	FP	N	FP
	T110	3.38	c	N	FN	N	FN	N	FN
	I205	5.46	nc	N	TN	N	TN	N	TN

	V254	6.51	nc	N	TN	Y	FN	N	TN
	T277	7.42	nc	N	TN	Y	FN	N	TN
				**Recall: 0.00 Precision: 0.00**		**Recall: 0.00 Precision: 0.00**		**Recall: 0.00 Precision: 0.00**	
**hOR2AG1/ amylbutyrate (0)**	A104	3.32	c	Y	TP	Y	TP	Y	FP
	V260	6.55	c	N	FN	Y	TP	N	FN
	S263	6.58	c	N	FN	N	FN	N	FN
	V264	6.59	c	N	FN	N	FN	N	FN
	T279	7.42	c	N	FN	N	FN	Y	TP
				**Recall: 0.20 Precision: 1.00**		**Recall: 0.40 Precision: 1.00**		**Recall: 0.25 Precision: 0.50**	
**hOR2M3/ 3-mercapto-2-methyl-pentan-1-ol (0)**	V78	2.58	c	N	FN	N	FN	N	FN
	I198	5.38	c	N	FN	N	FN	Y	TP
	Y259	6.55	c	Y	TP	N	FN	Y	TP
	R266	ECL3	c	N	FN	N	FN	N	FN
				**Recall: 0.25 Precision: 1.00**		**Recall: 0.00 Precision: 0.00**		**Recall: 0.50 Precision: 1.00**	
**hOR7D4/ androstadienone (0)**	S75	2.55	c	N	FN	N	FN	N	FN
	P79	2.59	c	N	FN	N	FN	N	FN
	S84	2.64	c	N	FN	N	FN	N	FN
	R88	ECL1	c	N	FN	N	FN	N	FN
	L162	4.63	c	N	FN	N	FN	N	FN
	A279	7.42	c	Y	TP	Y	TP	Y	TP
				**Recall: 0.17 Precision: 1**		**Recall: 0.17 Precision: 1.00**		**Recall: 0.17 Precision: 1.00**	
**hOR7D4/ androstenone (0)**	S75	2.55	c	N	FN	N	FN	N	FN
	P79	2.59	c	N	FN	N	FN	N	FN
	S84	2.64	c	N	FN	N	FN	N	FN
	R88	ECL1	c	N	FN	N	FN	N	FN
	L162	4.63	c	N	FN	N	FN	N	FN
	A279	7.42	c	Y	TP	Y	TP	Y	TP
				**Recall: 0.17 Precision: 1.00**		**Recall: 0.17 Precision: 0.00**		**Recall: 0.17 Precision: 1.00**	

Section 2.3: **Analysis of the Haddock docking results**. Using HADDOCK (Dominguez et al., 2003), seven predictions show high precision (Figure 1A, red circles), though with either low recall (those for the hOR7D4 in complex with androstenone and androstadienone, hOR2AG1 in complex with amylbutyrate and hOR2M3 in complex with 3-mercapto-2-methylpentan-1-ol) or modest recall (hTAS2R1 in complex with dextromethorphan, hTAS2R30 in complex with denatonium, and hTAS2R43 in complex with 6-nitrosaccharin). One prediction shows top recall (but modest precision); this is the prediction for hTAS2R31 in complex with the aristolochic acid (Figure 1A, yellow circle). Six predictions (those for hTAS2R38 in complex with its agonists propylthiouracil and phenyltiocarbamide, hTAS2R43 with its agonist IMNB, and hOR1A1 in complex with its agonists citronellol, (*R*)- and (*S*)-carvone) turn out to feature both zero precision and zero recall (Figure 1A, dark blue circles). As many as eight predictions feature intermediate values for the two performance metrics (Figure 1A, cyan circles).

Section 2.4: Analysis of Vina docking results. Using Autodock Vina (Trott and Olson, 2010), the docking results can also be grouped in four different clusters (Figure 1B). Three predictions (those for the hOR7D4 in complex with androstenone and androstadienone, along with that for hOR2AG1 with amylbutyrate) show high precision, but low or modest recall (Figure 1B, red circles). One prediction shows top recall but modest precision, i.e. that for hTAS2R1 receptor in complex with dextromethorphan (Figure 1B, yellow circle). Seven predictions (those for hTAS2R10 in complex with parthenolide, hTAS2R31 in complex with aristolochic acid, hTAS2R38 in complex with its two agonists PTC and PROP, hTAS2R46 in complex with strychnine, and hOR1A1 in complex with its agonists citronellol and (*R*)-carvone) turn out to feature both zero precision and zero recall (Figure 1B, dark blue circles). Finally eleven predictions, both for human bitter taste and olfactory receptors, present intermediate values for the two performance metrics (Figure 1B, cyan circles).

Section 2.5: Analysis of Glide docking results. Using Glide (Friesner et al., 2004), two predictions (for hOR7D4 in complex with its agonists androstenone and androstadienone) show high precision, but low recall (Figure 1C, red circles), similar to HADDOCK and AutoDock Vina predictions. Eleven predictions turn out to have zero precision and zero recall scores (Figure 1C, dark blue circles), while nine predictions (those for hTASR10 in complex with either denatonium or strychnine, hTAS2R16 in complex with arbutin or phenyl-β-D-glucopyranoside, and the complexes hTAS2R46/strychnine and hOR2AG1/amylbutyrate) have medium values of both performance parameters (Figure 1C, cyan circles).

Section 3: Structural analysis of the docking results

Section 3.1: Protein/ligand interactions predicted for each of the docking programs. The hChem-GPCR/agonist complexes are indicated in the same order as in Supplementary Table 1. All the images were generated with LigPlot$^+$ (Laskowski and Swindells, 2011), using a distance cut-off of 5.5 Å (i.e. the same threshold as in Supplementary Table 1). Chemical interactions are represented as dashed lines (hydrogen bonds and salt bridges) or spoked arcs (hydrophobic, stacking and electrostatic interactions). Note that the definition of the prediction outcome (TP, FP, TN or FN, see Figure 2 in the main text) includes both the distance threshold and the presence (or absence) of an actual chemical interaction. Therefore, Supplementary Figures 2-4 (see below) include TPs and FPs (highlighted with green and red boxes, respectively) and other predicted interacting residues for which experimental data are not yet available (and thus cannot be categorized using the binary classifier).

Supplementary Figure 2. 2D representation of the agonist binding cavity predicted by Haddock (Dominguez et al., 2003).

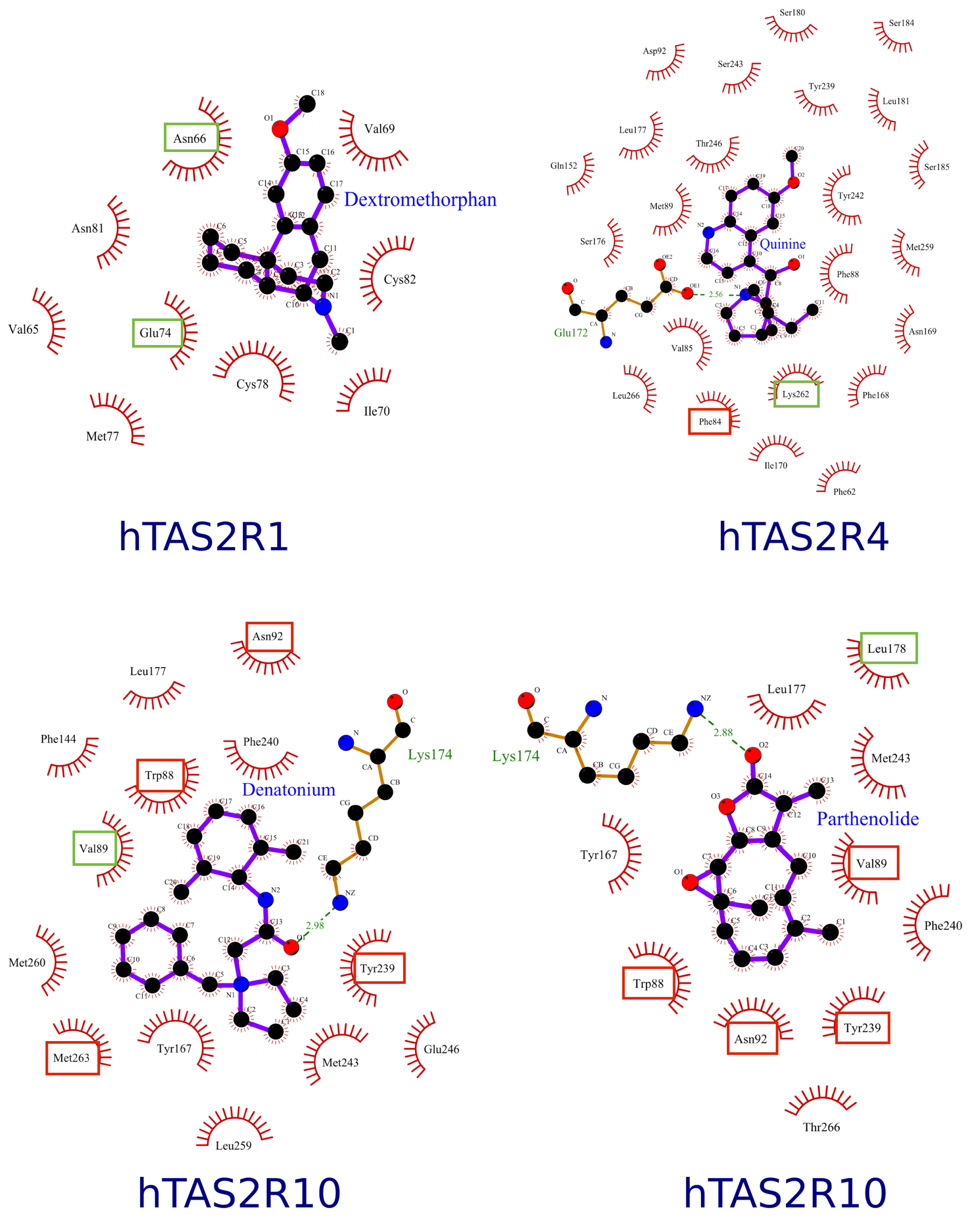

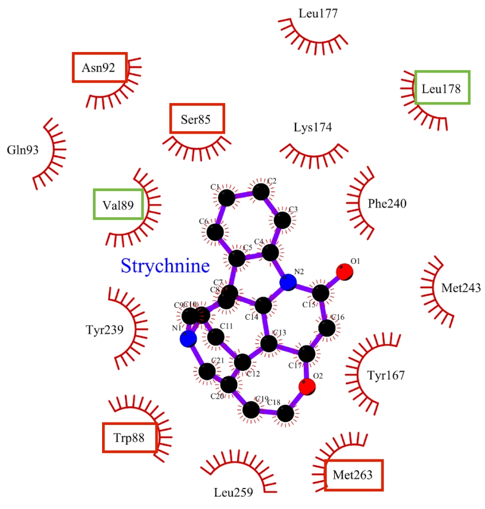

hTAS2R10

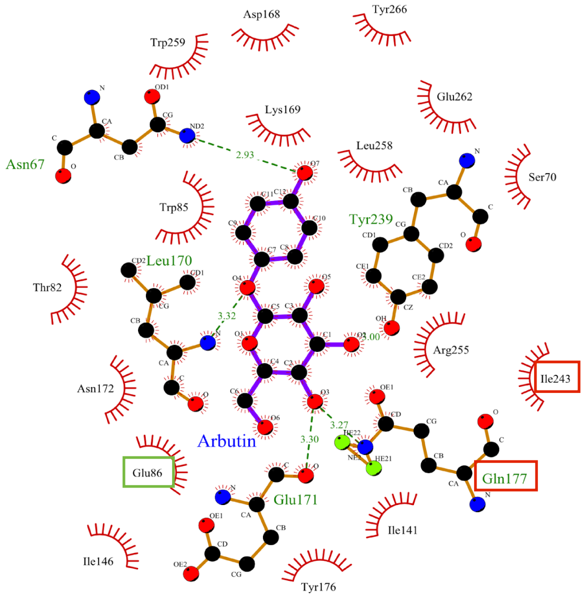

hTAS2R16

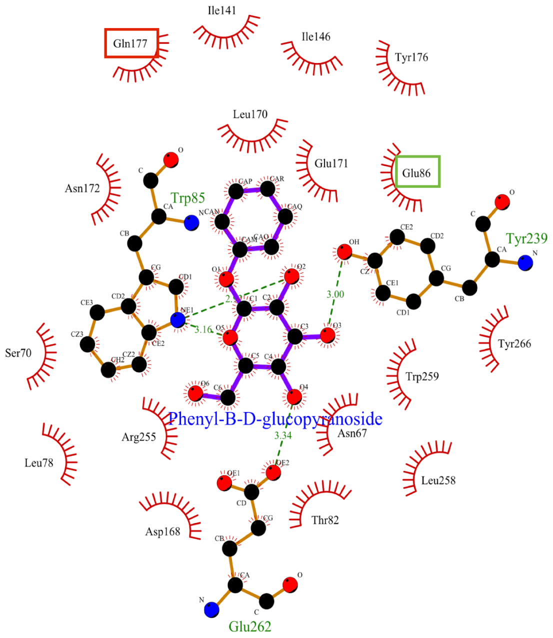

hTAS2R16

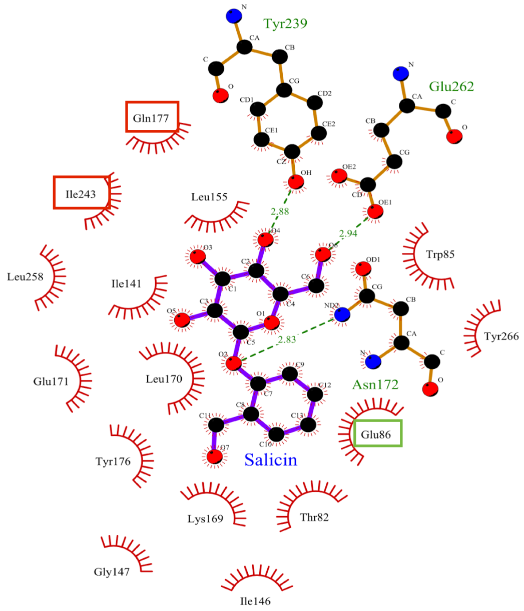

hTAS2R16

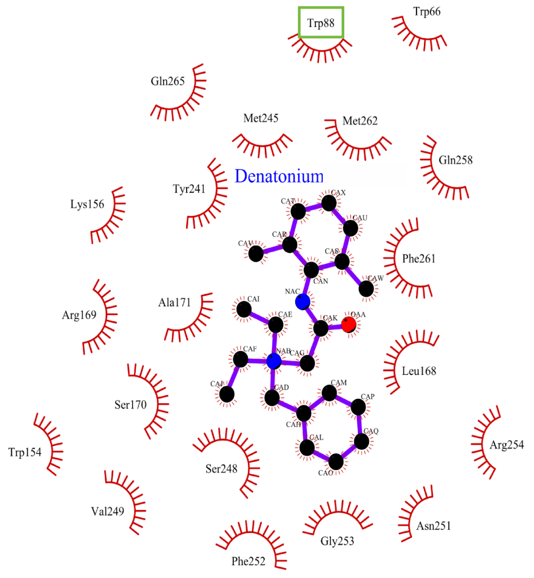

hTAS2R30

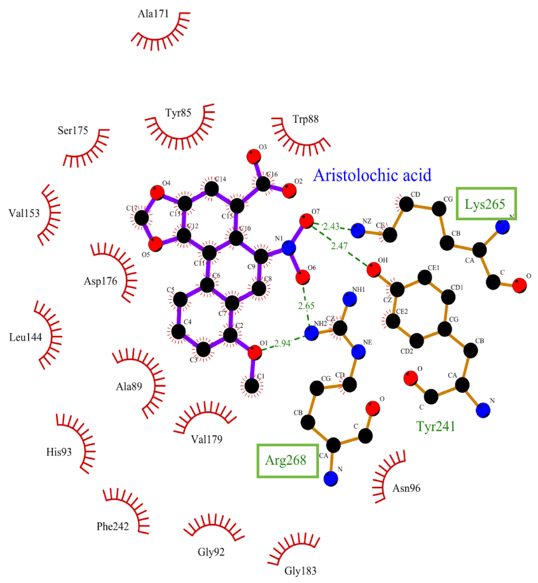

hTAS2R31

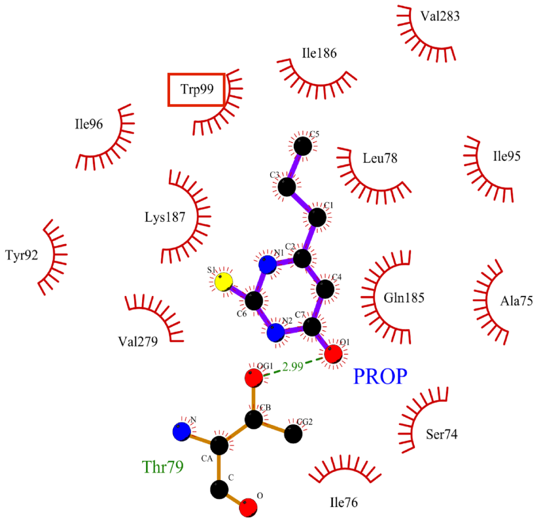

hTAS2R38

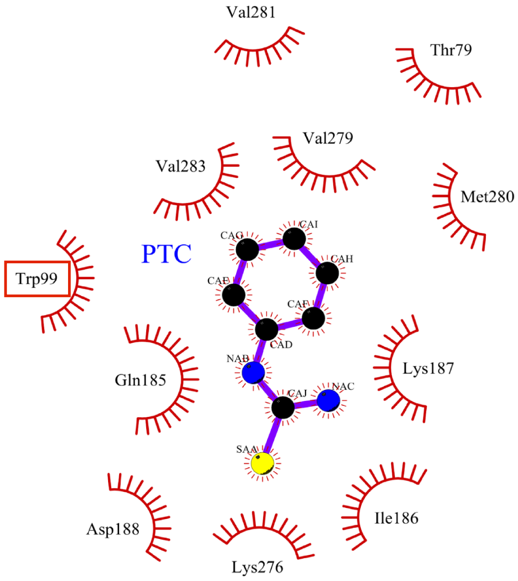

hTAS2R38

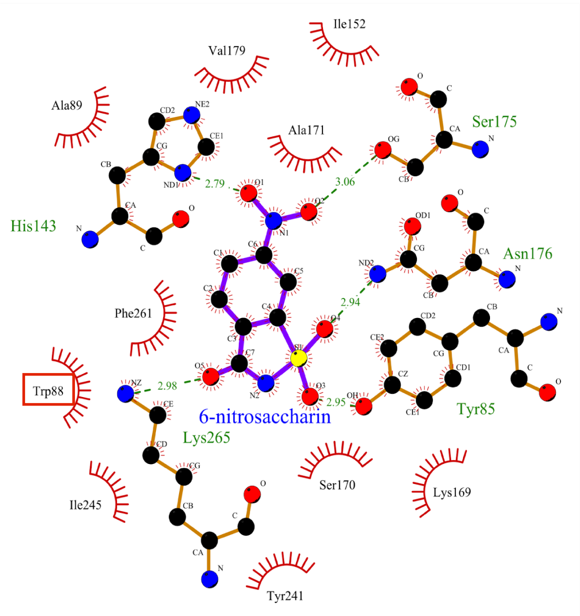

hTAS2R43

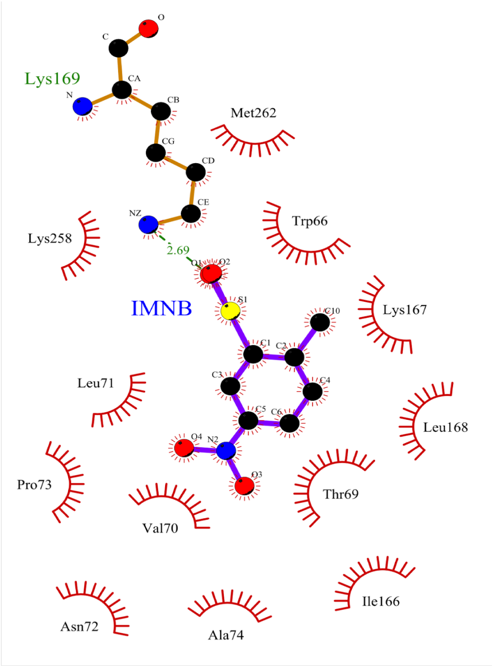

hTAS2R43

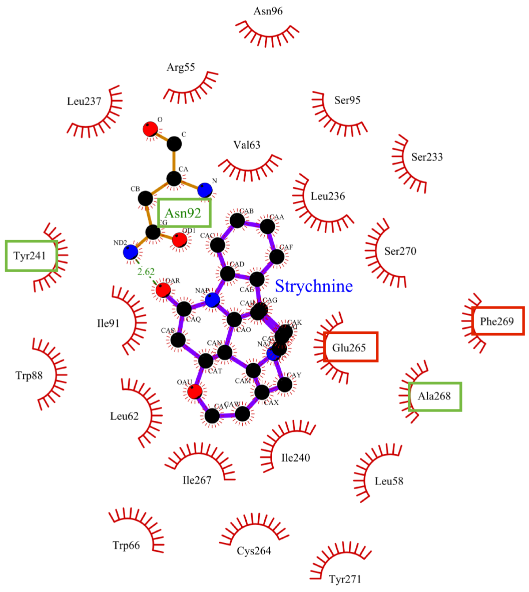

hTAS2R46

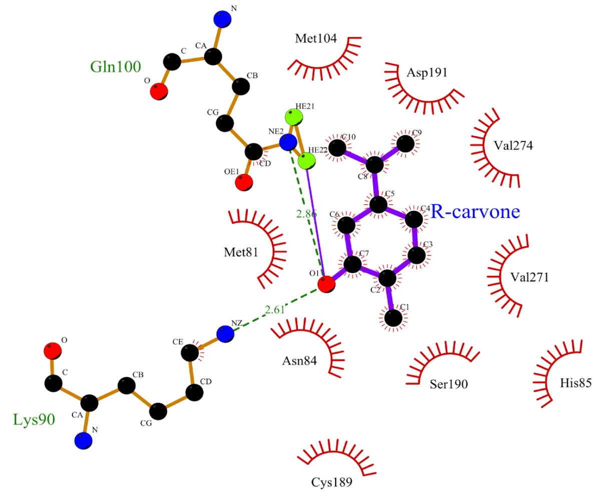

hOR1A1

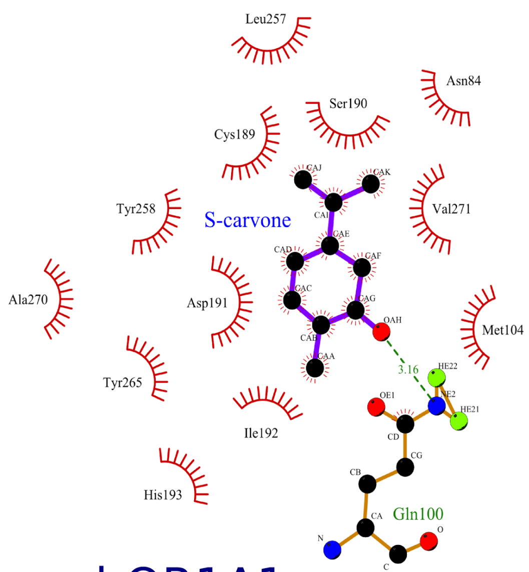

hOR1A1

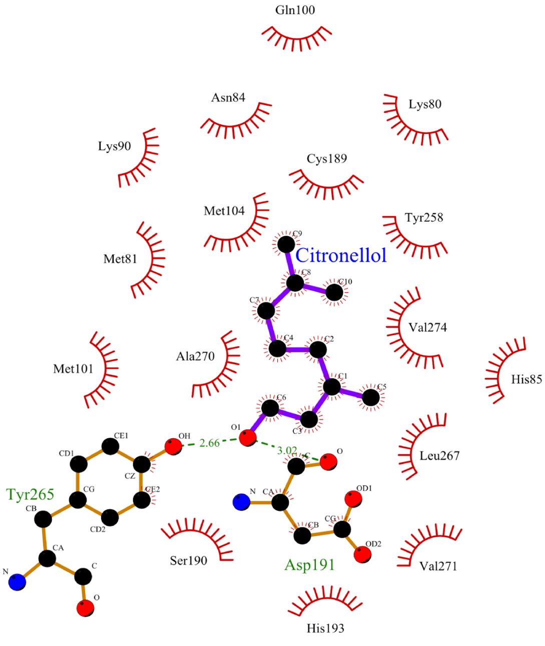

hOR1A1

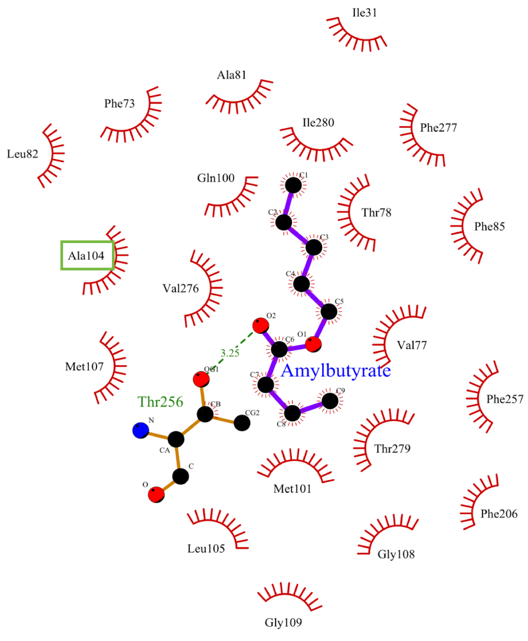

hOR2AG1

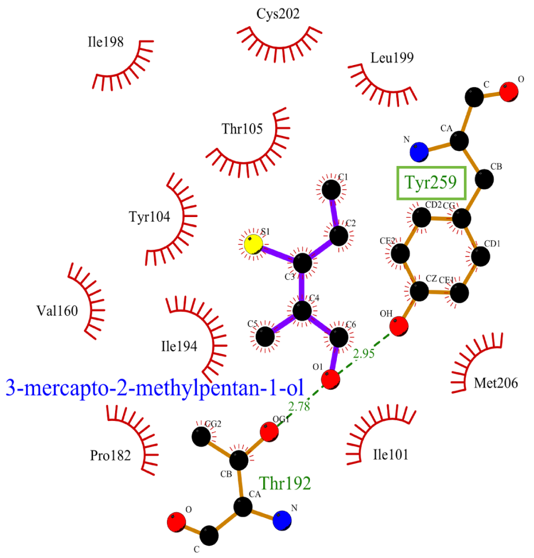

hOR2M3

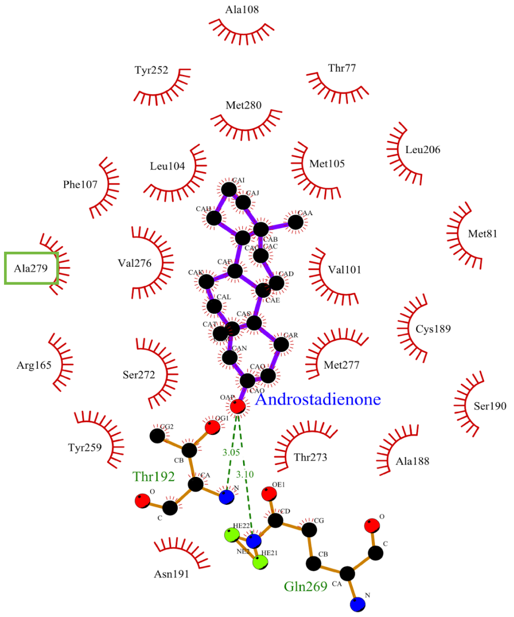

hOR7D4

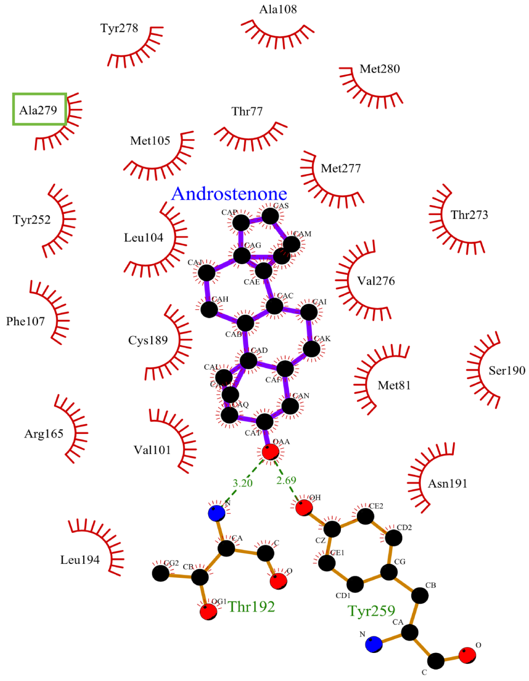

hOR7D4

Supplementary Figure 3. 2D representation of the agonist binding cavity predicted by Autodock Vina (Trott and Olson, 2010).

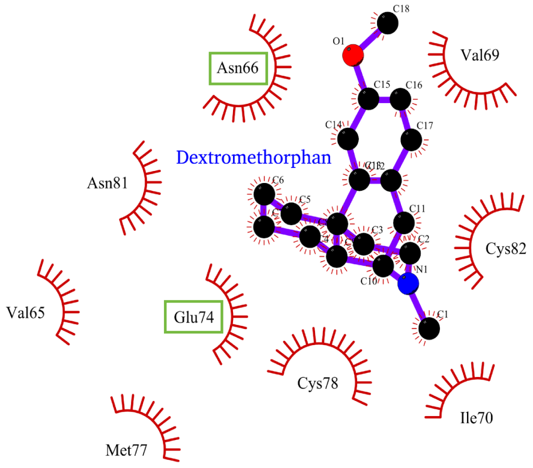

hTAS2R1

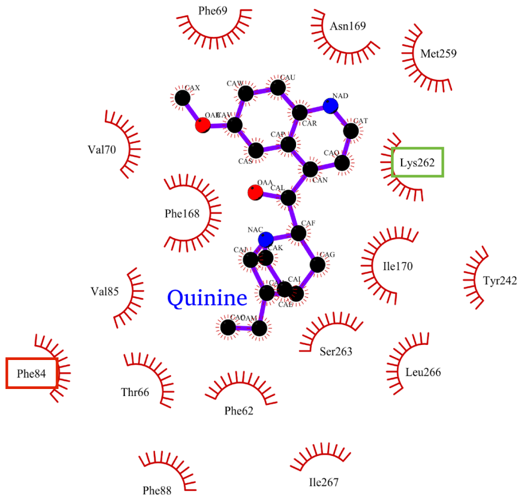

hTAS2R4

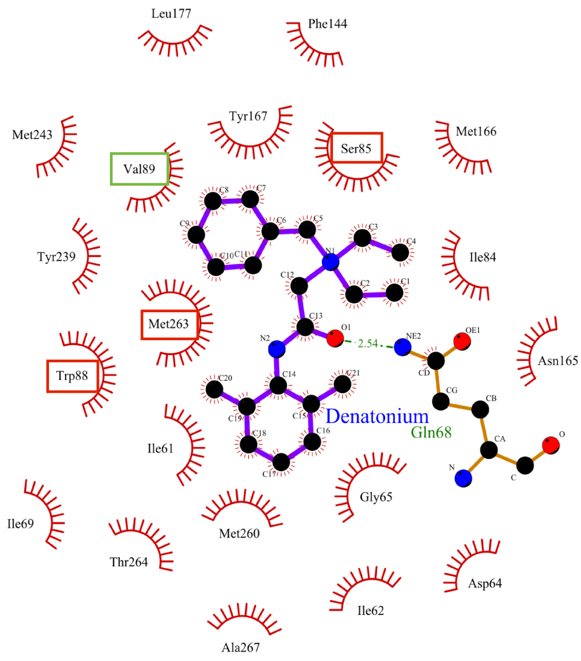

hTAS2R10

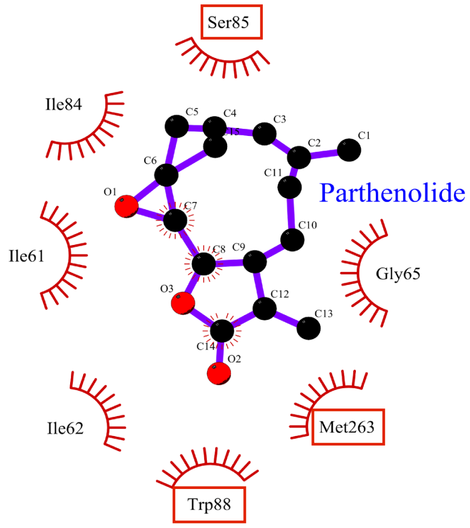

hTAS2R10

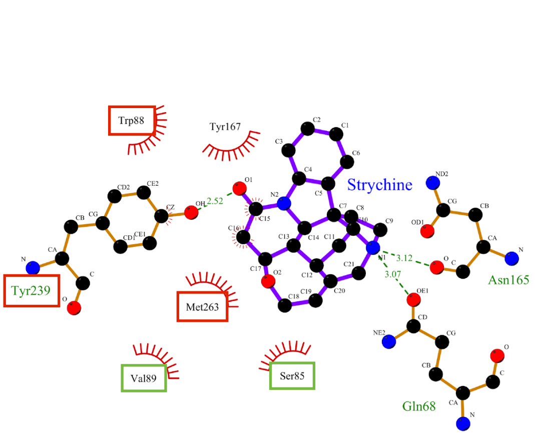

hTAS2R10

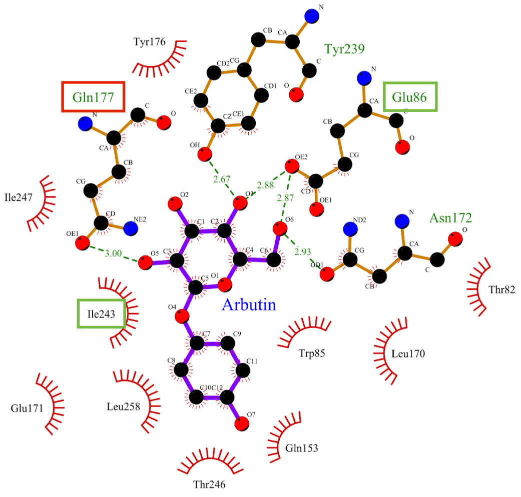

hTAS2R16

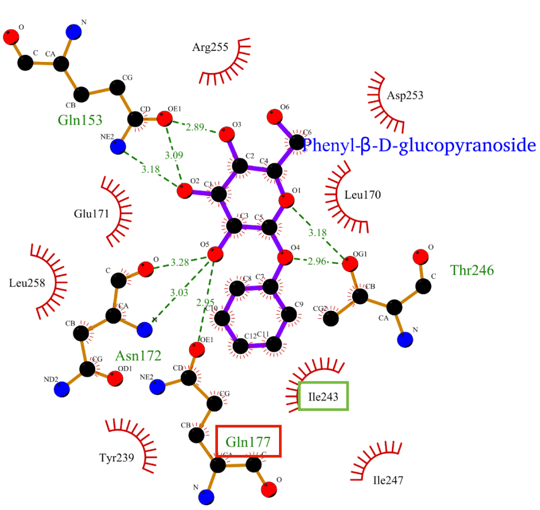

hTAS2R16

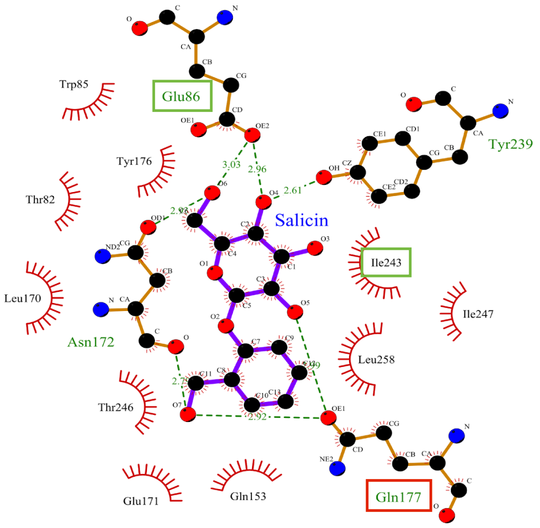

hTAS2R16

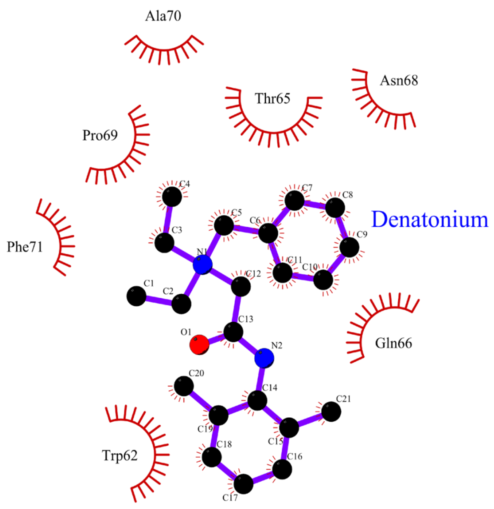

hTAS2R30

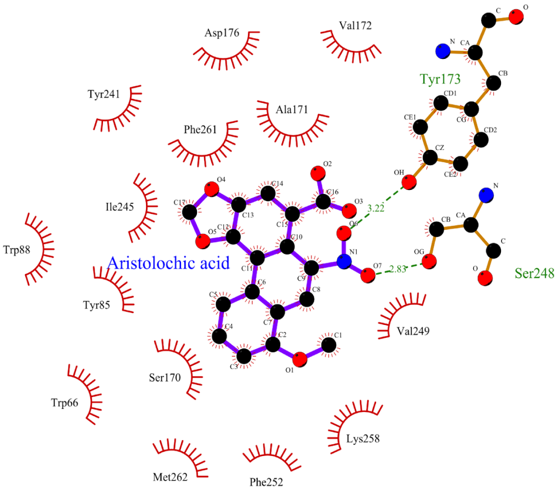

hTAS2R31

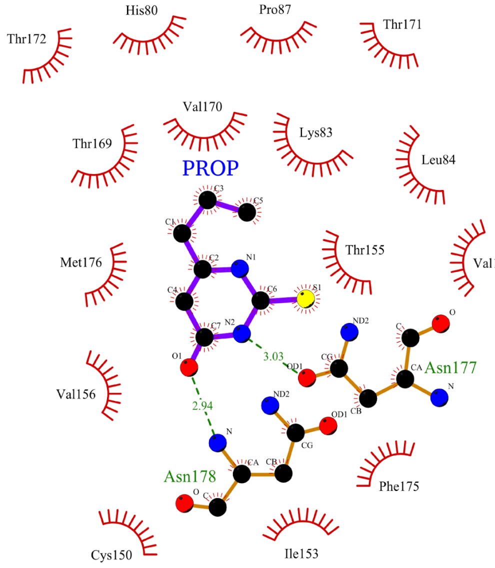

hTAS2R38

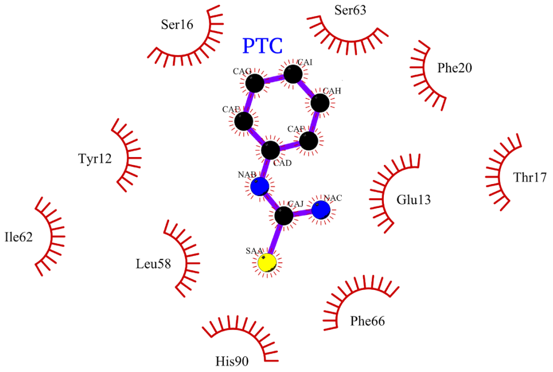

hTAS2R38

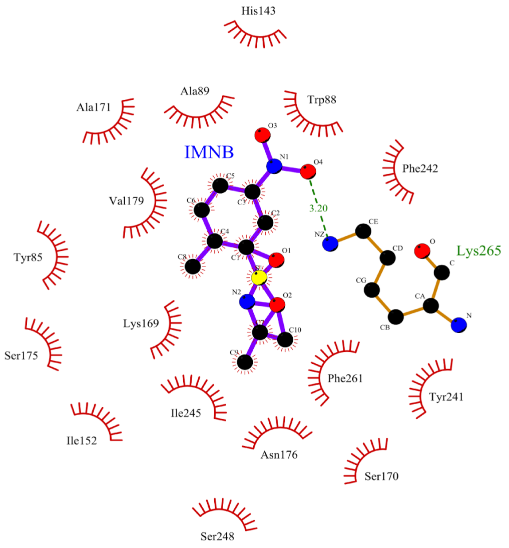

hTAS2R43

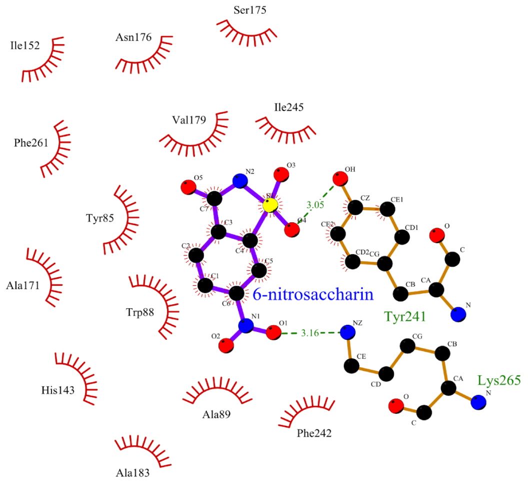

hTAS2R43

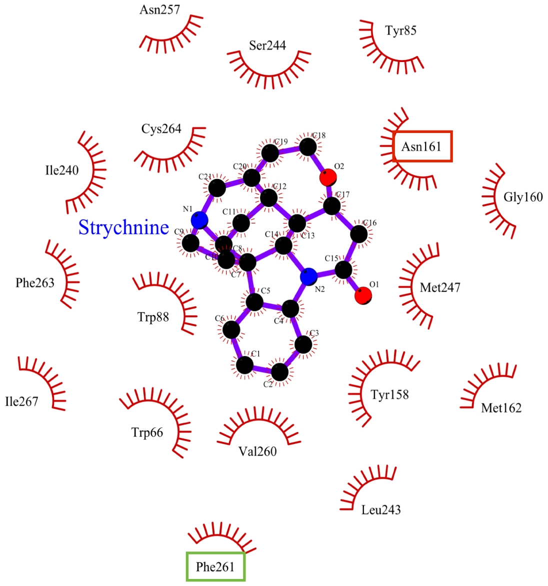

hTAS2R46

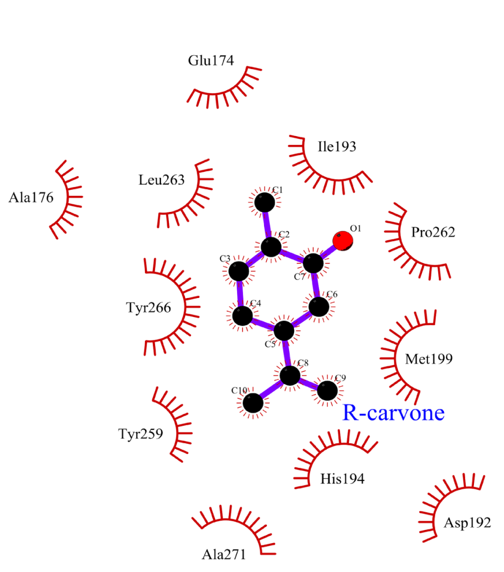

hOR1A1

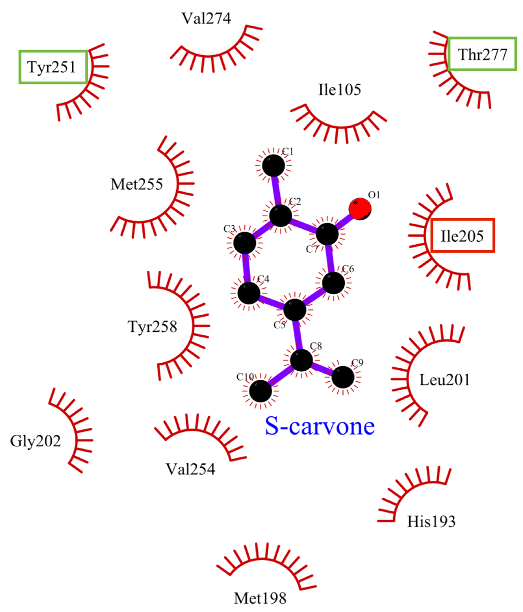

hOR1A1

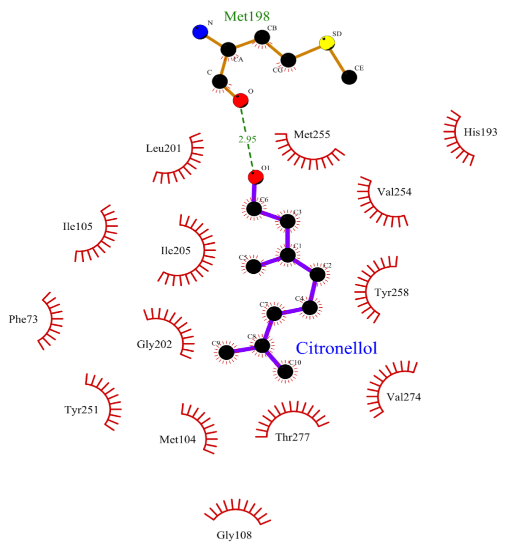

hOR1A1

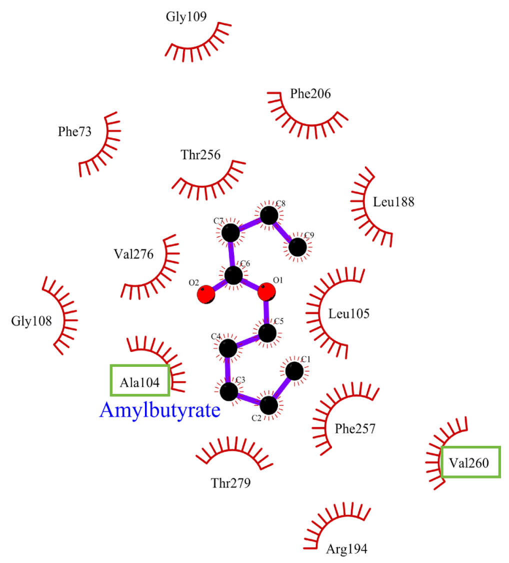

hOR2AG1

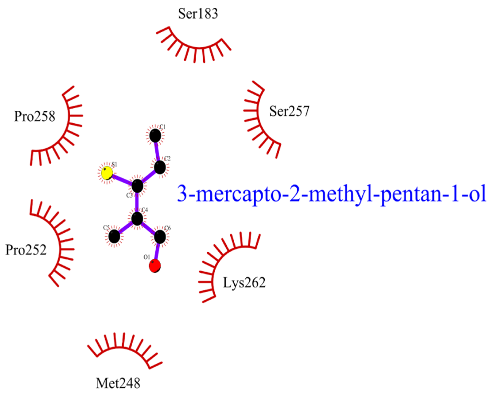

hOR2M3

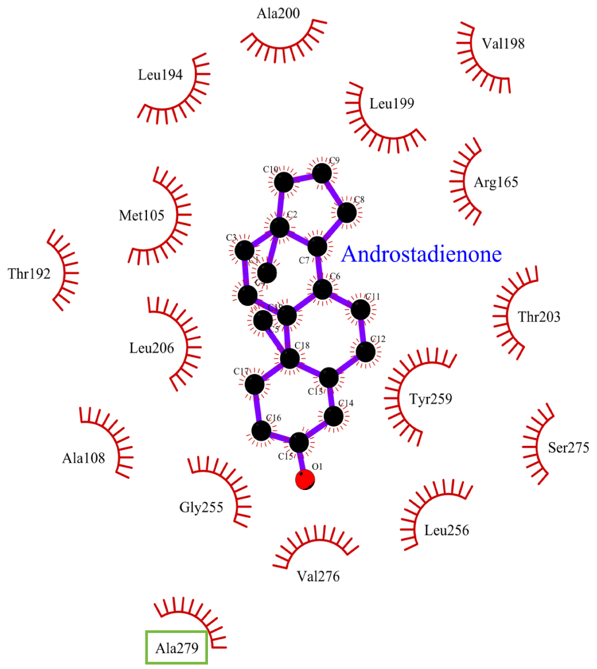

hOR7D4

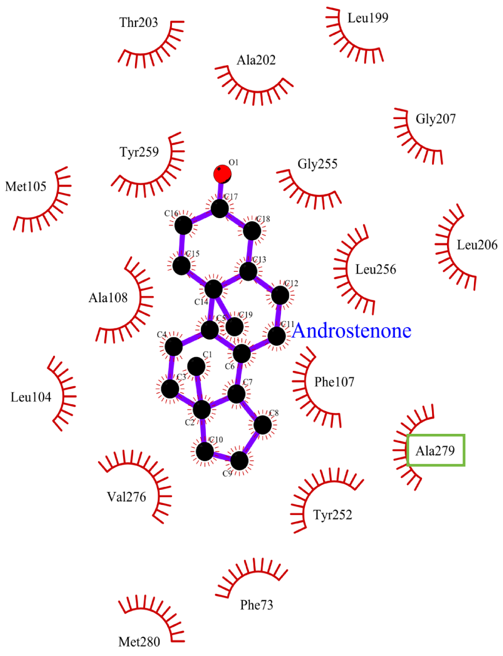

hOR7D4
