# Supplementary material for: Agonist Binding to Chemosensory Receptors: A Systematic Bioinformatics Analysis
Source: Front Mol Biosci. 2017 Sep 6;4:63. doi: 10.3389/fmolb.2017.00063 (PMC5592726; doi:10.3389/fmolb.2017.00063)
Supplement: Supplementary file 2 [file DataSheet2.pdf]

Agonist binding to chemosensory receptors: a systematic bioinformatics analysis

Supplementary Information

Part 2

Fabrizio Fierro[1,†], Eda Suku[2,†], Mercedes Alfonso-Prieto[1,3,*], Alejandro Giorgetti[1,2,*], Sven Cichon[4,5,6], Paolo Carloni[1,7,8]

[1] Computational Biomedicine, Institute for Advanced Simulation IAS-5 and Institute of Neuroscience and Medicine INM-9, Forschungszentrum Jülich, 52425 Jülich, Germany.

[2] Department of Biotechnology, University of Verona, Ca' Vignal 1, Strada le Grazie 15, 37134 Verona, Italy.

[3] Cécile and Oskar Vogt Institute for Brain Research, Medical Faculty, Heinrich Heine University Düsseldorf, Merowingerplatz 1a, 40225 Düsseldorf, Germany.

[4] Institute of Neuroscience and Medicine INM-1, Forschungszentrum Jülich, 52425 Jülich, Germany.

[5] Institute for Human Genetics, Department of Genomics, Life&Brain Center, University of Bonn, Sigmund-Freud-Straße 25, 53127 Bonn, Germany.

[6] Division of Medical Genetics, Department of Biomedicine, University of Basel, Switzerland.

[7] Department of Physics, Rheinisch-Westfälische Technische Hochschule Aachen, 52062 Aachen, Germany.

[8] VNU Key Laboratory "Multiscale Simulation of Complex Systems", VNU University of Science, Vietnam National University, Hanoi, Viet Nam.

[†] These authors have contributed equally to this work.

**** Correspondence:***

Mercedes Alfonso-Prieto, m.alfonso-prieto@fz-juelich.de

Alejandro Giorgetti, a.giorgetti@fz-juelich.de

Supplementary Figure 4. 2D representation of the agonist binding cavity predicted by Glide (Friesner et al., 2004).

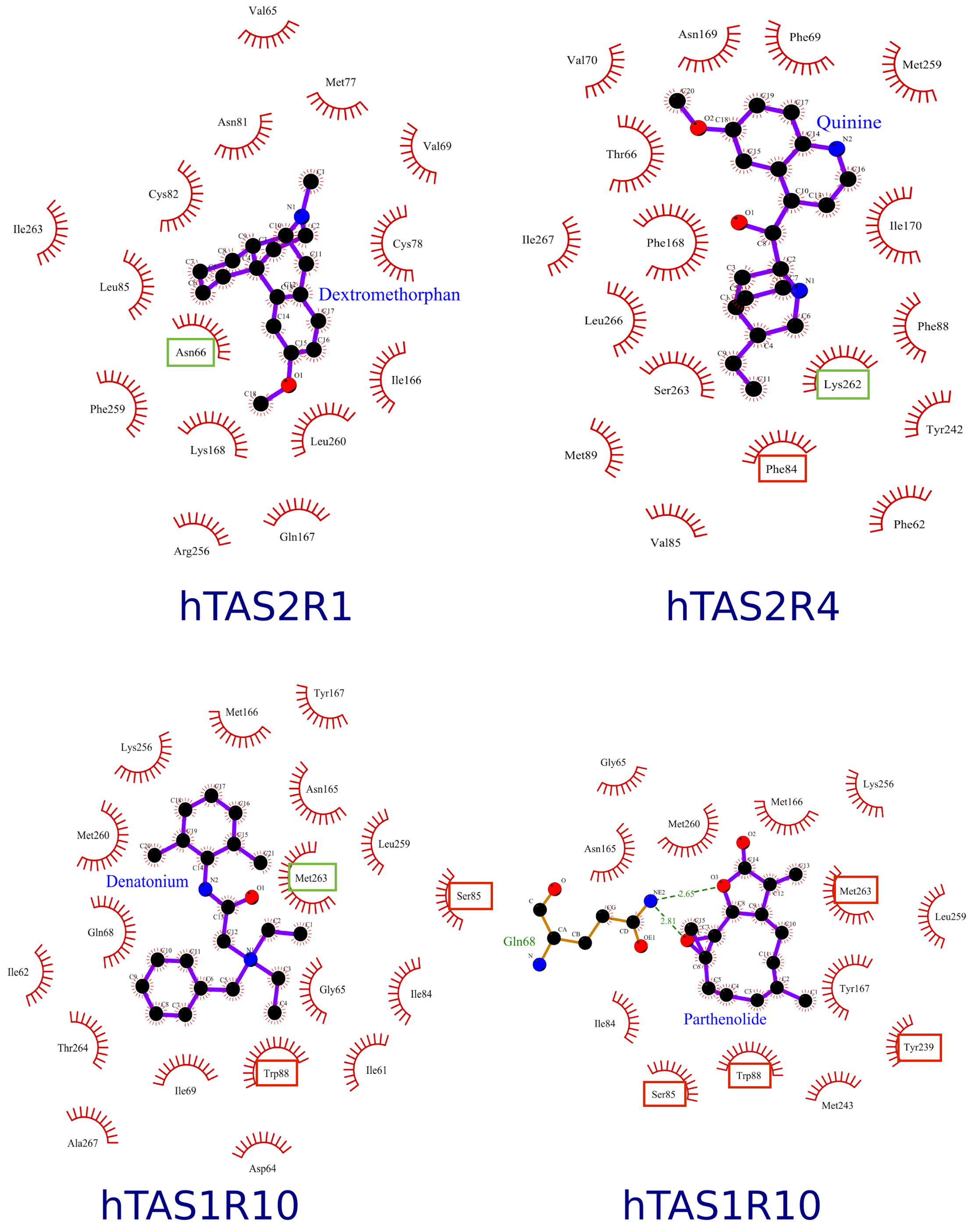

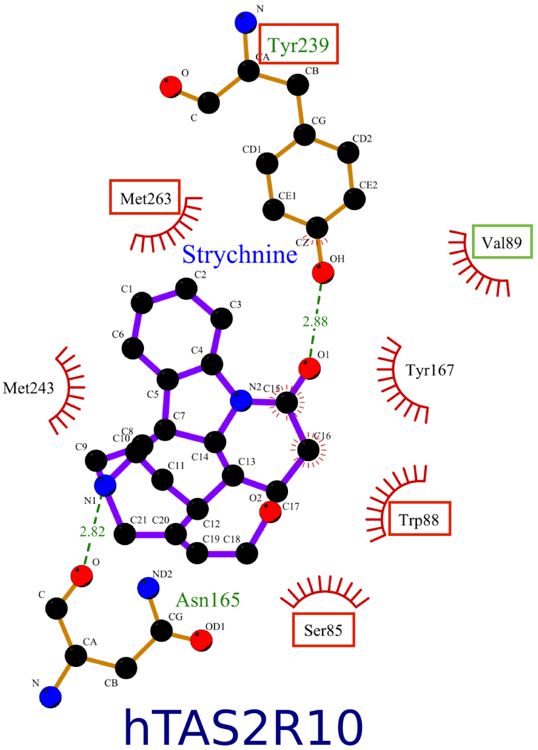

hTAS2R10

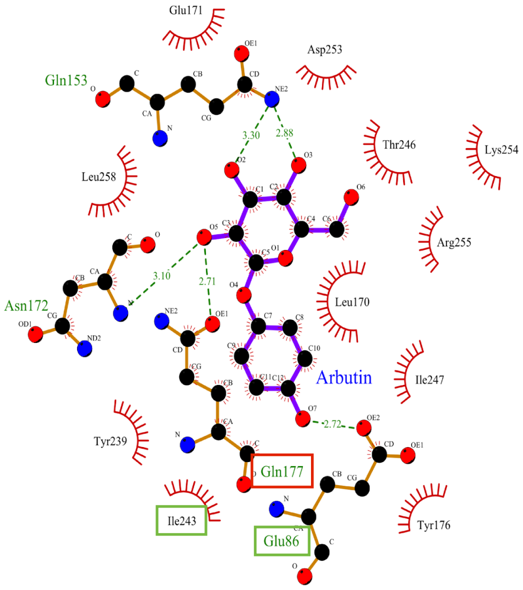

hTAS2R16

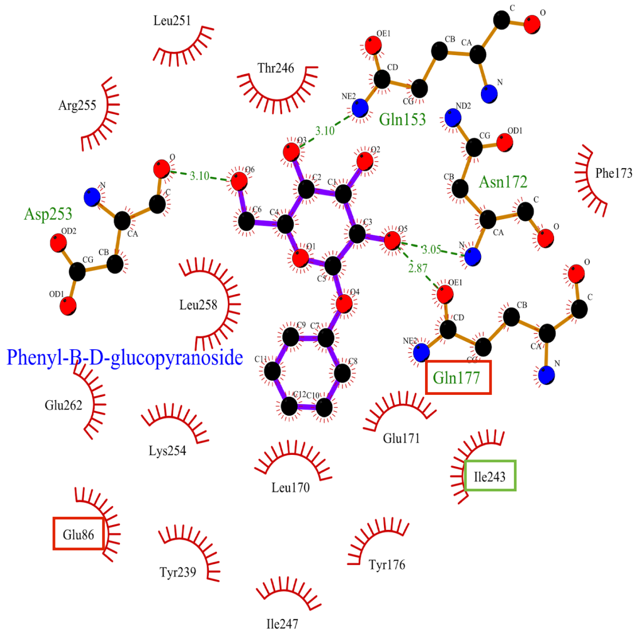

hTAS2R16

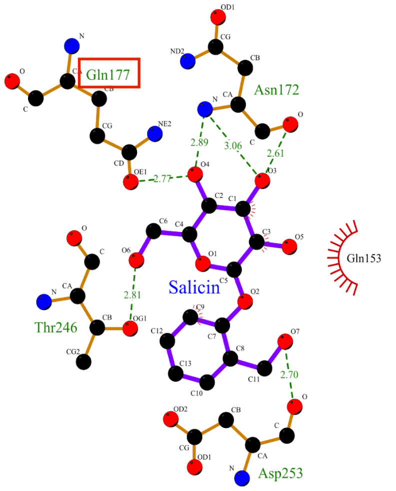

hTAS2R16

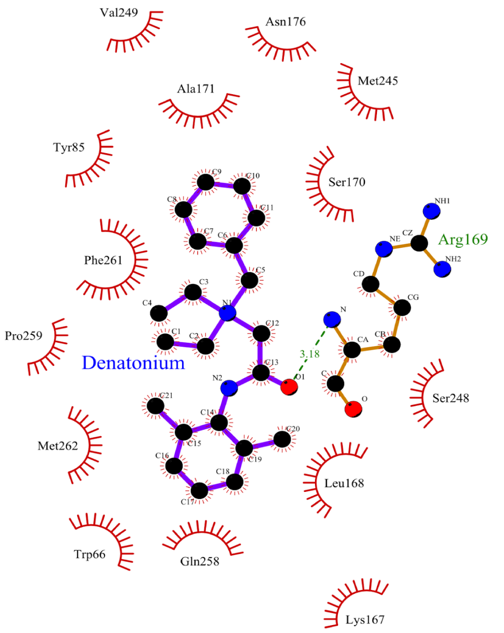

hTAS2R30

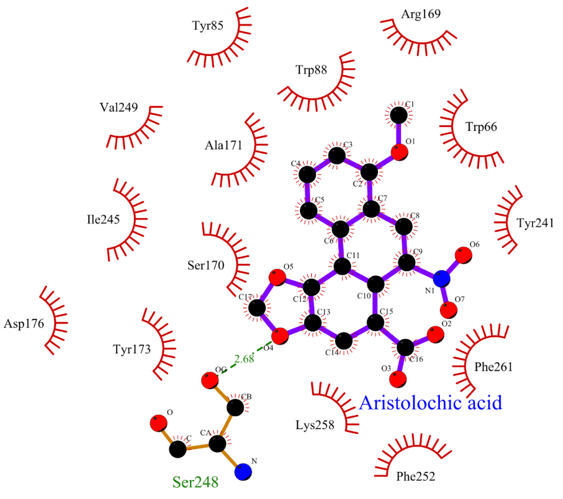

hTAS2R31

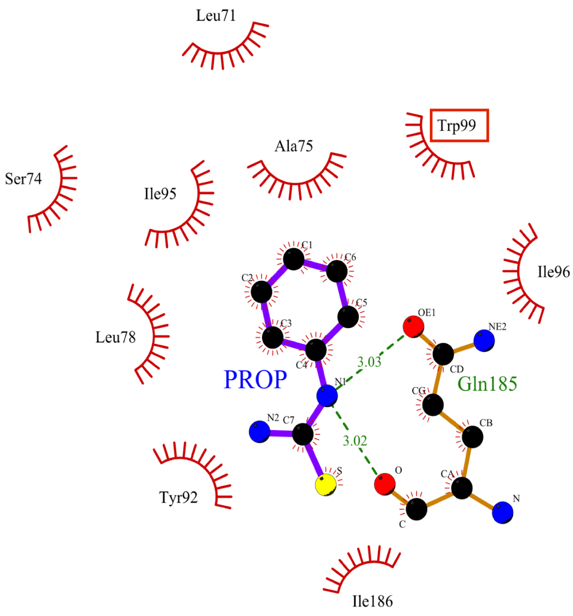

hTAS2R38

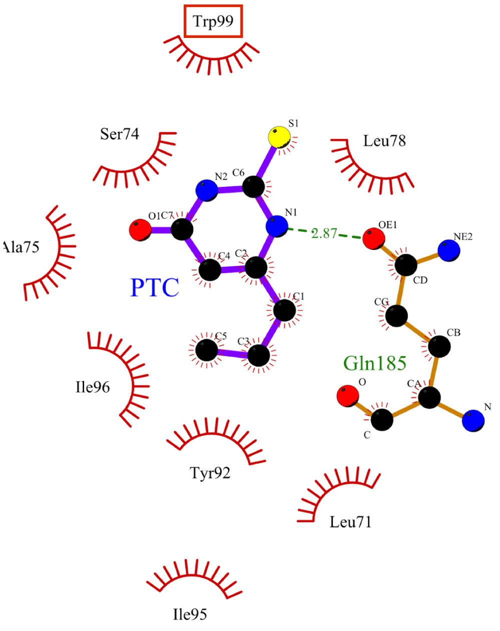

hTAS2R38

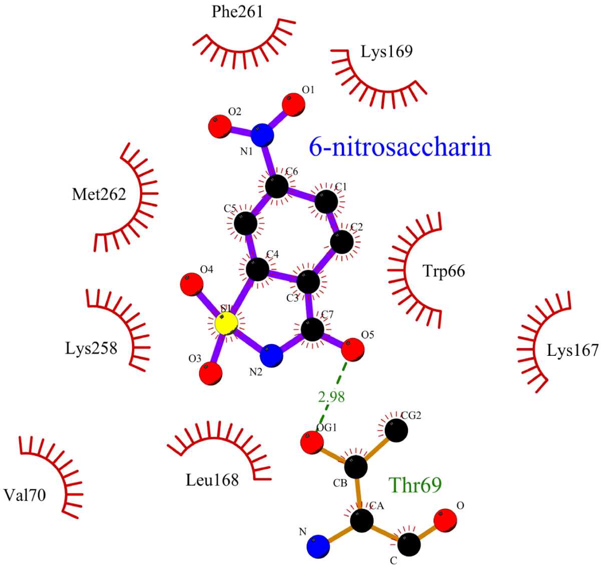

hTAS2R43

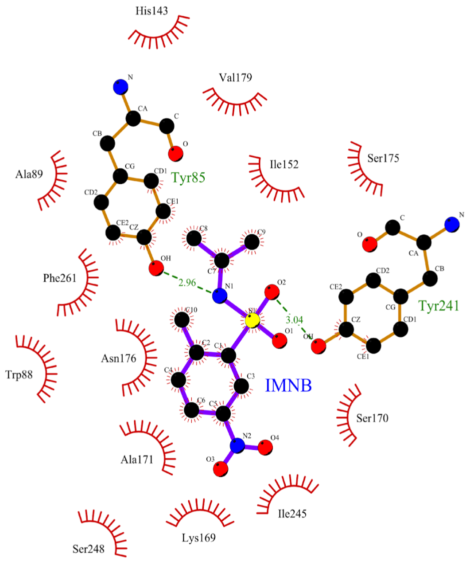

hTAS2R43

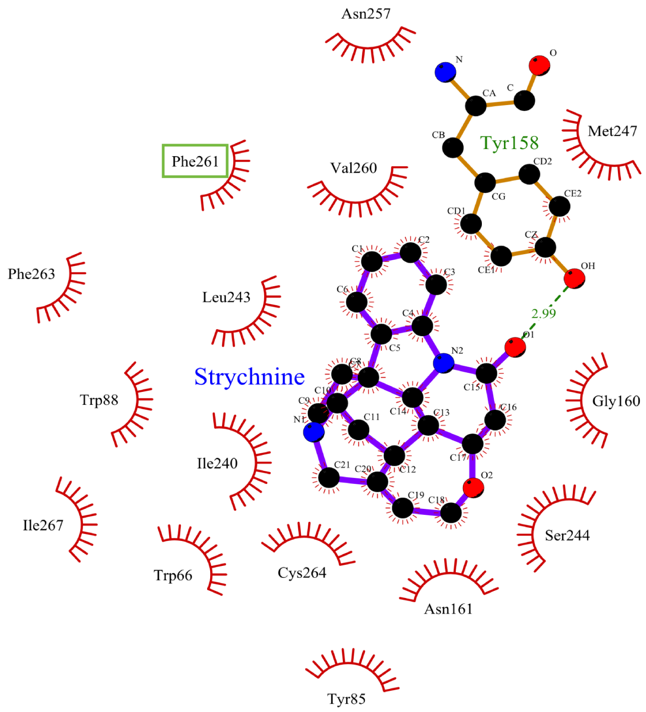

hTAS2R46

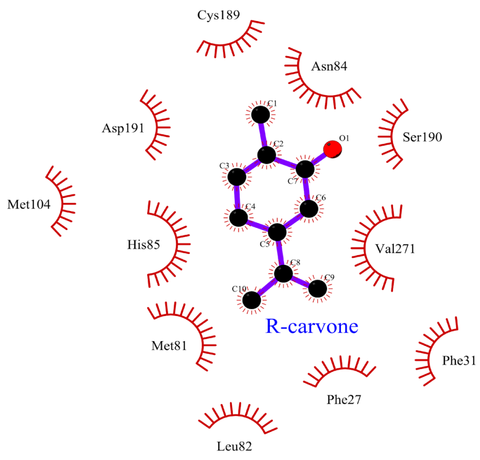

hOR1A1

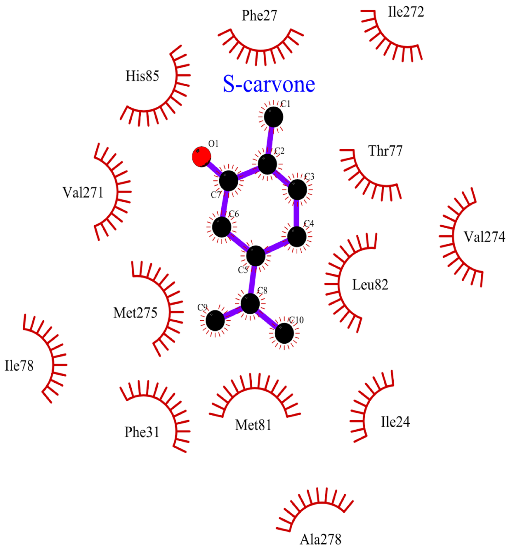

hOR1A1

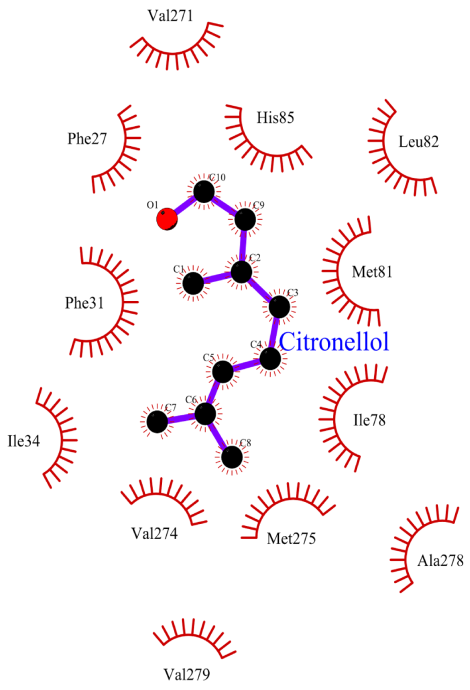

hOR1A1

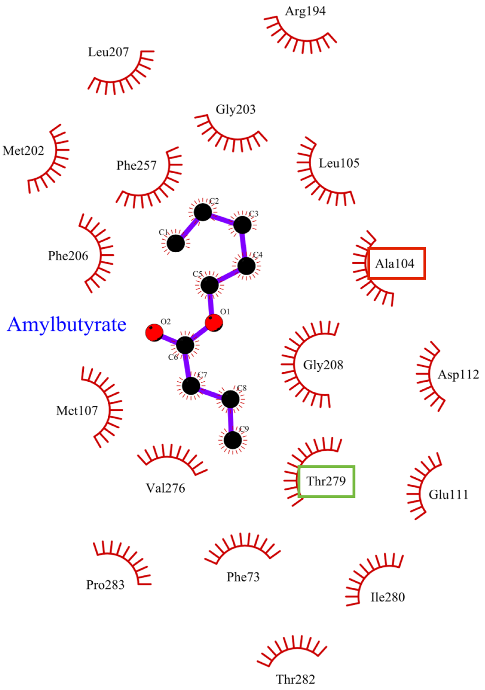

hOR2AG1

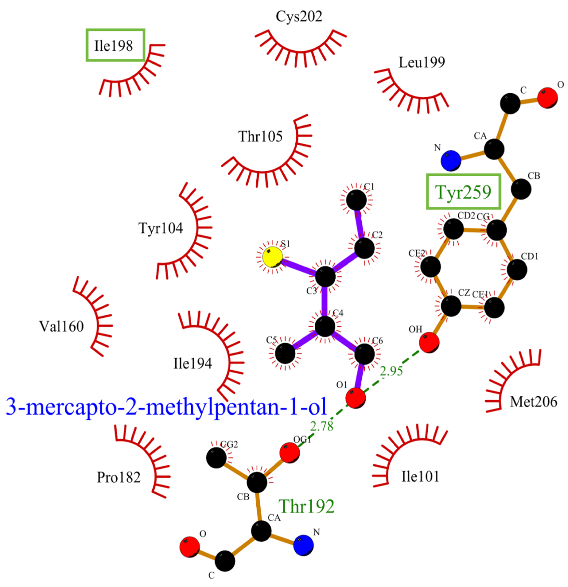

hOR2M3

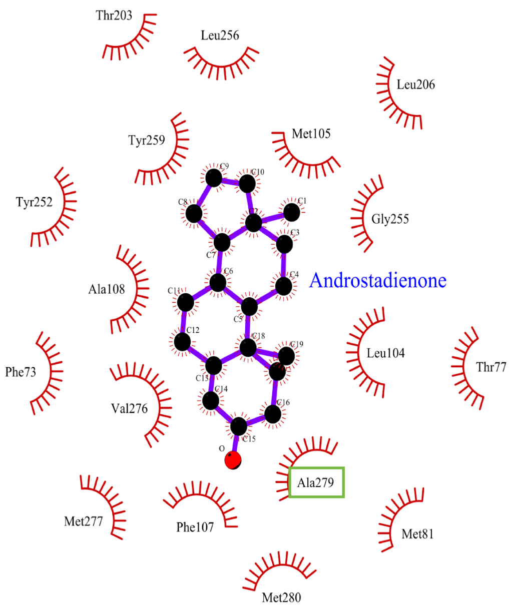

hOR7D4

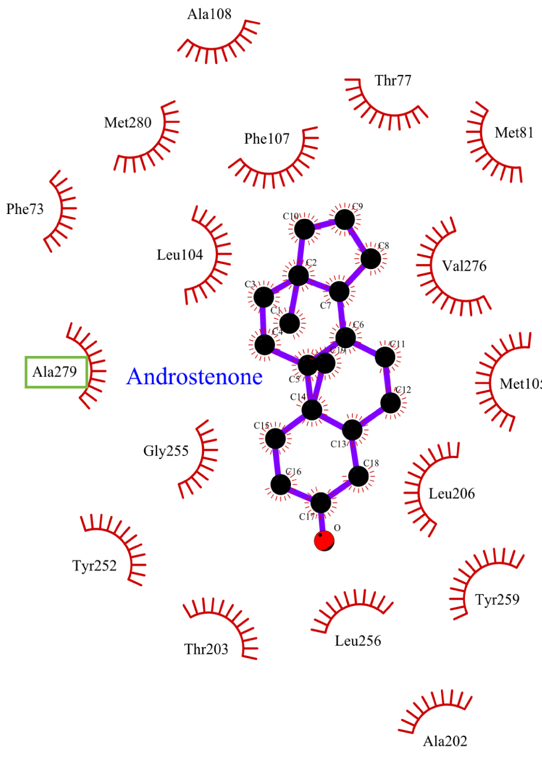

hOR7D4

Section 4: Accuracy of the MM/CG simulation results

Supplementary Table 2. Analysis of the simulation data of the three hChemGPCR/agonist complexes for which both experimental studies and MM/CG molecular dynamics have been performed (Marchiori et al., 2013; Sandal et al., 2015). The first column lists those three hTAS2R/agonist complexes, along with the ligand charge in parentheses. The second column shows the residues for which mutagenesis data are available. Residues belonging to the bottom half of the receptor, to the N- or C-termini, and to the TM8 have been omitted, as they are well outside the canonical orthosteric binding site of class A GPCRs (Venkatakrishnan et al., 2013) and thus they are expected not to be involved in ligand binding. In the third column, residues are numbered accordingly to the GPCRdb numbering scheme (Isberg et al. 2015) of their template, i.e. the human β2 adrenoceptor (PDB code: 4LDE) for the two hTAS2R38 complexes and the human dopamine D3 receptor (PDB code: 3PBL) for hTAS2R46 (Sandal et al., 2015). In the fourth column, interpretation of EC_{50} values for the corresponding residues is reported using the following nomenclature: c = change in EC_{50}; nc = no significant change in EC_{50}. In the following columns is indicated whether the residue interacts with the ligand along the MM/CG simulation (Y=yes, N=no) and the prediction outcome for this residue (TP=true positive, TN=true negative, FP=false positive, FN=false negative, see the main text and Figure 3 for the definition), depending on the presence or absence of an actual chemical interaction.

hChem-GPCR/ agonist complex (ligand charge)	Experimental data			MMCG	
	**Residue**	**Position**	**EC_{50}**	**Interaction**	**Prediction**
**hTAS2R38/ Phenylthiocarbamide (0)**	W99	3.32	nc	N	TN
	N103	3.36	c	Y	TP
	N179	ECL2	nc	N	TN
	R181	ECL2	nc	N	TN
	N183	ECL2	nc	N	TN
	F197	5.42	nc	Y	FP
	W201	5.46	c	Y	TP
	F255	6.47	nc	N	TN
	F264	6.56	c	Y	TP
				**Recall: 1.00**<br>**Precision: 0.75**	

hChem-GPCR/ agonist complex (ligand charge)	Experimental data			MMCG	
	Residue	Position	EC_{50}	Interaction	Prediction
**hTAS2R38/ propylthiouracil (0)**	W99	3.32	nc	N	TN
	M100	3.33	nc	N	TN
	N103	3.36	c	Y	TP
	N179	ECL2	nc	N	TN
	R181	ECL2	nc	N	TN
	N183	ECL2	nc	N	TN
	F197	5.42	c	Y	TP
	W201	5.46	c	Y	TP
	F197	5.42	c	Y	TP
	F264	6.56	c	Y	TP
				**Recall: 1.00 Precision: 1.00**	
**hTAS2R46/ strychnine (+1)**	E70	2.65	c	Y	TP
	L71	ECL1	c	Y	TP
	I82	3.26	c	Y	TP
	N92	3.36	c	Y	TP
	N150	ECL2	nc	N	TN
	N161	ECL2	nc	N	TN
	N176	5.39	c	Y	TP
	Y241	6.51	c	Y	TP
	E253	ECL3	c	Y	TP
	F261	7.35	c	Y	TP
	E265	7.39	c	Y	TP
	A268	7.42	c	Y	TP
	F269	7.43	c	Y	TP
				**Recall: 1.00 Precision: 1.00**	

Section 5: Bioinformatics analyses of receptors' activation

Supplementary Table 3. Available experimental mutagenesis data on residues putatively involved in receptor activation in hTAS2Rs. The generic numbering for class A GPCRs (Isberg et al. 2015) (based on the GPCRdb numbering of the template used in this work, PDB code: 4LDE) and the GPCR residue numbering used in the original publication are listed in columns 1 and 2, respectively. The human bitter taste receptor and the corresponding mutation reported in the literature, together with its activation-related effect, are indicated in columns 3-5, with the corresponding reference in column 6. Finally, column 7 lists the sequence conservation of this position in human bitter taste receptors. The novel position identified in this work (7.52) is highlighted in yellow.

**residue numbering**		**receptor**	**mutant**	**effect**	**reference**	**Conservation in hTAS2Rs**
**GPCRdb class A**	**original paper**					
1.50	1.50	hTAS2R1	N24A<br>N24D	Loss of agonist-induced signaling (>90%)	*(Singh et al., 2011)*	*92%*
1.53	1.53	hTAS2R1	I27A<br>I27V	Receptor hyperactivity	*(Singh et al., 2011)*	*96%*
2.50	2.54	hTAS2R1	R55A	Receptor hyperactivity	*(Singh et al., 2011)*	*96%*
5.63	5.53	hTAS2R4	H214A	Constitutively active mutant	*(Pydi et al., 2014)*	*96%*
ICL3	ICL3	hTAS2R4	Q216A	Constitutively active mutant	*(Pydi et al., 2014)*	*Not conserved*
6.35	ICL3	hTAS2R4	V234A	Constitutively active mutant	*(Pydi et al., 2014)*	*Not conserved*
6.38	ICL3	hTAS2R4	M237A	Constitutively active mutant	*(Pydi et al., 2014)*	*Not conserved*
7.50	7.47	hTAS2R4	S285A	Constitutively active mutant	*(Pydi et al., 2014)*	*S= 68%<br>P= 28%*
**7.52**	**7.52**	hTAS2R38	I296V	Change in activation of the receptor	*(Biarnes et al., 2010)*	*I/L/V = 92%*

Supplementary Table 4. Effect of mutations in residues involved in the activation network of class A GPCRs. Residues experimentally shown as important for activation are indicated with the generic residue numbering for class A GPCRs (Isberg et al. 2015). The novel position 7.52 identified in this work is highlighted in yellow.

residue numbering	receptor	mutants	effect	reference
**1.50**	TSHR	N1.50D	Disrupting important, architecture-stabilizing intramolecular interactions and ultimately leading to the complete intracellular retention of the receptor.	*(Labadi et al., 2015)*
**2.46**	A2aAR	L2.46A	Stabilization; Increase in binding affinity of agonist; 50-fold decrease of binding affinity for antagonist	*(Lebon et al., 2011)*
	Muscarinic M1 and M5	L2.46A	Reduced efficacy	*(Hulme, 2013)*
	TSHR	L2.46A	Increased basal activity	*(Urizar et al., 2005)*
	TSHR	L2.46I	No effect	*(Urizar et al., 2005)*
	TSHR	L2.46W	Decreasing basal activity	*(Urizar et al., 2005)*
	Rhodopsin	L2.46A	Increasing basal activity	*(Madabushi et al., 2004)*
**2.50**	Opioid	D2.50A	Reducing agonist-dependent signaling of some GPCRs, while maintaining ligand binding and often basal signaling	*(Fenalti et al., 2014)*
**3.39**	Opioid	S3.39A	Disrupting of normal ligand dependent signaling	*(Katritch et al., 2014)*
**3.40**	Muscarinic M1 and M5	V3.40A	Increasing agonist affinity	*(Hulme, 2013)*
	MOR136-1	S3.40A	Abolishing agoinist binding	*(Ho et al., 2015)*
	Histamine H1R	I3.40A/G	Lower basal activity and lower agonist response	*(Sansuk et al., 2011)*
	TSHR	V3.40A	Constitutive activity	*(Duprez et al., 1994)*
**3.43**	S1P1	L3.43E/G	Abolishing activation	*(Fujiwara et al., 2007)*
	B2AR	L3.43R/K/A	Increased basal activity	*(Tao et al., 2000)*
	Muscarinic M1	L3.43A	Constitutive activity	*(Lu and Hulme, 1999)*

**3.43 (*cont.*)**	TSHR	L3.43Q/N/R	Constitutive activity	*(Kosugi et al., 2000)* *(Nishihara et al., 2006)* *(Trulzsch et al., 2001)*
	C5A	L3.43A	Constitutive activity	*(Baranski et al., 1999)*
	CB1	L3.43A	Constitutive activity; elevated basal cAMP accumulation; enhanced affinity for agonists and diminished affinity for inverse agonists.	*(D'Antona et al., 2006)*
**5.54**	FSHR	I5.54L/T/F/N	Constitutive activity	*(Tao, 2008)*
**5.58**	TSHR	Y5.58F	Decreasing basal activity	*(Kleinau et al., 2008)*
	B2AR	Y5.58A	Stabilization of inactive conformation	*(Tate and Schertler, 2009)*
**6.35**	Rhodopsin	R6.35C	Decreasing basal activity	*(Dunham and Farrens, 1999)*
**6.37**	Vasopressin V2	I6.37L	Reducing ability to bind the G-protein	*(Venkatakrishnan et al., 2016)*
**6.39**	5-HT2A	I6.39A	Increasing affinity for agonist (indirect effect)	*(Shapiro et al., 2002)*
	AT1R	A6.39C	Cysteine mutant is sensitive to treatment with MTSEA	*(Martin et al., 2007)*
**6.40**	C5A	V6.40A	Increasing basal activity	*(Baranski et al., 1999)*
	Muscarinic M5	I6.40S	Constitutive activity	*(Spalding et al., 1998)*
	Histamine H1R	I6.40E/G/A /R/K/S	Constitutive activity	*(Sansuk et al., 2011)* *(Bakker et al., 2008)*
	Rhodopsin	M6.40Y	Constitutive activity	*(Deupi et al., 2012)*
	AT1R	I6.40T	Increasing basal activity	*(Parnot et al., 2000)*
	Melanocortin-4	L6.40Q	Increasing basal activity	*(Vaisse et al., 2000)*
	TSHR	L6.40F	Increasing basal activity	*(Tonacchera et al., 1998)*

**6.40 (*cont.*)**	Lutropin-choriogonadotropic	L6.40A	Constitutive activity	*(Fanelli, 2000)*
	S1P1	V6.40A/T	Reducing activation	*(Fujiwara et al., 2007)*
	S1P1	V6.40L	Abolishing activation	*(Fujiwara et al., 2007)*
**6.44**	Muscarinic M5	F6.44S/T/L	Constitutive activity	*(Spalding et al., 1998)*
	Rhodopsin	F6.44T/V	Increasing basal activity	*(Han et al., 1997)*
	Rhodopsin	F6.44A	No significant change in basal activity	*(Han et al., 1997)*
	Rhodopsin	F6.44W	Decreasing basal activity	*(Han et al., 1997)*
	A1B adrenergic	F6.44L	Increasing basal activity	*(Greasley et al., 2002)*
	A1B adrenergic	F6.44Y	No significant change in basal activity	*(Greasley et al., 2002)*
	A1B adrenergic	F6.44A/G	Decreasing basal activity	*(Greasley et al., 2002)*
	Ghrelin	F6.44Y/L	Decreasing basal activity	*(Valentin-Hansen et al., 2012)*
	Ghrelin	F6.44A	Increasing basal activity	*(Valentin-Hansen et al., 2012)*
	GPR119	F6.44A	Decreasing basal activity; loss of agonist-induced response	*(Valentin-Hansen et al., 2012)*
	B2AR	F6.44A	Decreasing basal activity; loss of agonist-induced response	*(Valentin-Hansen et al., 2012)*
	NK1	F6.44A	Decreasing basal activity; loss of agonist-induced response	*(Valentin-Hansen et al., 2012)*
	GPR39	L6.44A/Y	Decreasing basal activity	*(Valentin-Hansen et al., 2012)*
	GPR39	L6.44F	Decreased basal activity; loss of agonist-induced response	*(Valentin-Hansen et al., 2012)*
	TSHR	D6.44A/E/H/Y	Constitutive activity	*(Porcellini et al.,1994; Parma et al., 1997; Russo et al., 1996)*

**6.48**	muscarinic M1 and M5	W6.48A	Reducing agonist response	*(Hulme et al. 2013)*
	Ghrelin receptor	W6.48A/H	Abolishing basal activity; reduced agonist response	*(Holst et al., 2010)*
	GPR119	W6.48A	Abolishing basal activity; reduced agonist response	*(Holst et al., 2010)*
	GPR39	W6.48A	Abolishing basal activity; reduced agonist response	*(Holst et al., 2010)*
	B2AR	W6.48A	Abolishing basal activity; reduced agonist response	*(Holst et al., 2010)*
	S1P1	W6.48A/E	Impaired activation	*(Fujiwara et al., 2007)*
**7.45**	adenosine	N7.45A	Removal of modulator allosteric effects but little effect on agonist binding	*(Gao et al., 2003)*
**7.49**	Opioid	N7.49D	Mutation eliminated detectable binding of either [3H]diprenorphine or [3H]DAMGO	*(Xu et al., 1999)*
**7.52**	TSHR	L7.52V	Increase of basal activity	*(Russo et al., 1999)*
	A2bAR	V7.52A	Reducing agonist-induced activation	*(Liu et al., 2015)*
	Melanocortin-1	I7.52T	Reducing basal activity	*(Lubrano-Berthelier et al., 2006)*
**7.53**	Vasopressin V2	Y7.53F/A	Reduced ability to bind to G-protein	*(Venkatakrishnan et al., 2016)*

Supplementary Table 5. Conservation of residues in position 7.52 across human class A GPCRs, hTAS2Rs and hORs.

**Residue conservation percentage**				
**Class A hGPCRs**	I=31.7%	L=30%	V=16%	M,F,Y,C=1% each
**hTAS2Rs**	I=76%	L=12%	V=4%	S,Q=4% each
**hORs**	I=88%	V=9%	L=1%	T,A,S<1% each

Supplementary Figure 5. Interactions of the residue in position 7.52 in the mammalian class A GPCR active/inactive structure pairs solved by X-ray crystallography. Inactive structures are shown in green, whereas active structures are in orange. The corresponding PDB codes are listed in Table 4 in the main text.

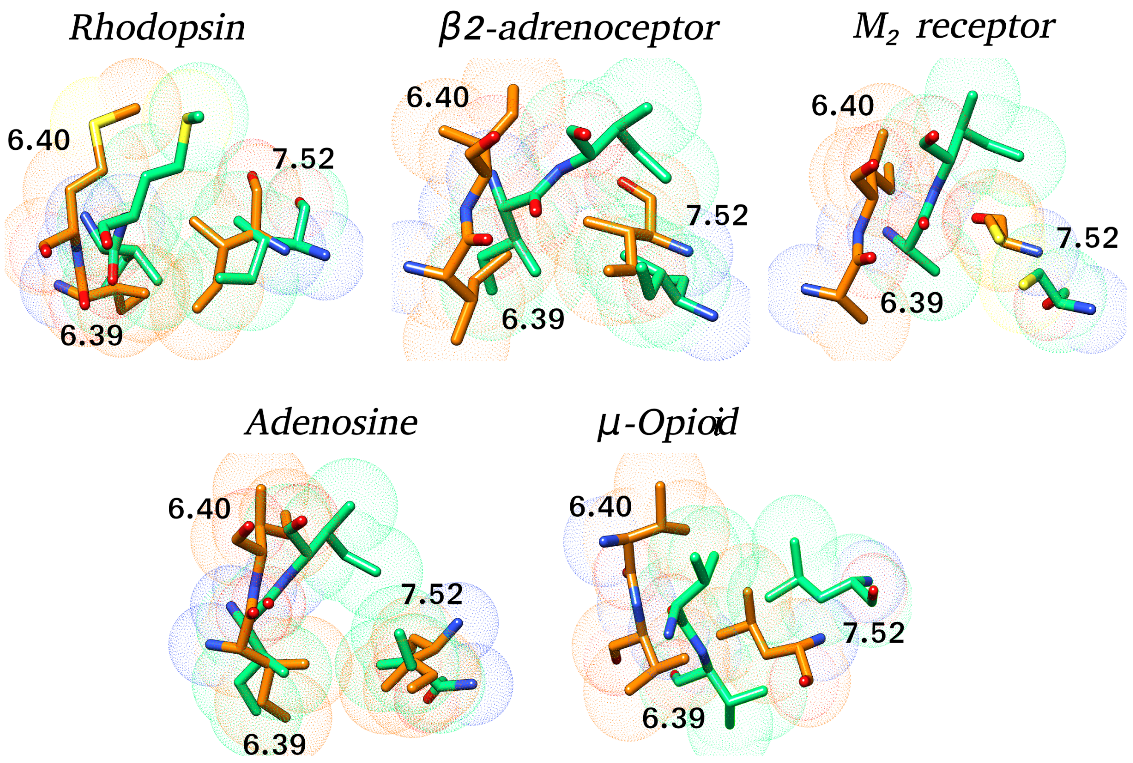

Section 6: Further details of the bioinformatics analysis

Supplementary Table 6. UniProt accession numbers of the hTAS2Rs' and hORs' sequences used in this work.

Human bitter taste receptors				Human odorant receptors	
**Receptor**	**UniProt ID**	**Receptor**	**UniProt ID**	**Receptor**	**UniProt ID**
hTAS2R1	Q9NYW7	hTAS2R31	P59538	hOR2AG1	Q9H205
hTAS2R10	Q9NYW0	hTAS2R38	P59533	hOR2M3	Q8NG83
hTAS2R16	Q9NYV7	hTAS2R43	P59542	hOR1A1	Q9P1Q5
hTAS2R30	P59541	hTAS2R46	P59540	hOR7D4	Q8NG98

Supplementary Table 7. Predicted binding cavity residues for hChem-GPCRs. The hChem-GPCRs studied in this work are indicated on the left and the putative binding residues, as predicted by fpocket (Le Guilloux et al., 2009), on the right.

hChem-GPCR	fpocket predicted residues
hTAS2R1	1 2 4 5 6 9 61 62 65 66 68 69 70 71 72 73 74 75 77 78 79 81 82 85 86 90 139 140 143 144 147 149 150 151 153 155 156 159 160 166 167 168 169 170 172 174 175 178 182 240 243 244 247 248 251 252 254 255 256 257 259 260 261 263 264
hTAS2R4	66 69 70 73 74 79 88 89 92 93 96 100 146 150 156 172 173 174 176 180 181 184 185 188 189 190 192 193 239 240 243 244 246 247 248 250 251 263 264 266 267 270 271 273
hTAS2R10	3 5 6 9 12 13 16 17 20 57 58 61 62 64 65 66 67 68 69 70 71 72 73 74 75 76 80 81 84 85 88 89 92 163 164 165 166 167 174 177 178 236 239 240 243 246 252 254 255 256 257 259 260 261 263 264 266 267 268 271
hTAS2R16	6 10 14 59 63 64 66 67 68 70 71 72 74 75 76 77 78 79 81 82 85 86 88 89 92 93 94 141 142 144 146 147 148 150 153 155 156 157 161 162 164 165 166 167 168 169 170 171 172 174 176 177 180 181 184 185 236 239 240 242 243 246 247 249 250 251 252 253 254 255 256 258 259 260 262 263 265 266 269
hTAS2R30	58 62 66 69 81 84 85 87 88 92 152 154 156 167 168 169 170 171 176 180 183 184 238 241 245 248 249 252 253 254 258 261 262 265 268 269
hTAS2R31	8 11 12 15 16 18 19 51 54 55 58 59 62 63 65 66 67 81 82 85 86 88 89 90 92 93 151 171 172 173 175 180 183 184 187 188 237 238 241 249 250 251 253 254 255 257 258 259 260 261 262 264 265 266 269

hTAS2R38	18 21 22 25 71 72 74 75 76 77 78 79 80 82 83 84 86 87 91 92 95 96 99 100 103 165 166 167 174 178 181 183 184 185 186 187 188 189 190 191 194 197 198 201 256 258 259 260 262 263 266 267 268 269 270 274 276 278 279 280 282 283 286
hTAS2R43	5 6 9 13 58 62 63 66 69 70 71 73 76 78 79 81 82 85 88 89 91 92 142 143 146 147 148 150 151 152 153 154 155 156 158 160 161 166 167 168 169 170 171 172 175 176 179 180 183 241 242 245 248 249 252 253 256 257 258 259 261 262 265 266 268 269
hTAS2R46	12 13 14 15 16 17 19 51 54 55 58 62 63 64 65 66 67 69 70 71 72 73 82 84 85 88 91 92 95 96 99 102 103 158 159 160 161 179 183 184 187 190 191 194 197 226 227 229 230 231 233 234 236 237 238 240 241 242 243 244 245 247 257 258 260 261 262 264 265 267 268 269 270 271 272 273 276
hOR1A1	1 4 5 6 8 9 12 15 19 51 54 55 57 58 59 61 62 63 65 66 67 69 70 71 73 77 80 81 82 84 85 86 89 141 146 148 154 156 157 159 161 168 169 170 171 172 173 174 175 176 178 179 182 183 186 232 235 236 238 239 242 243 246 248 249 251 252 253 255 256 258 259 260 262
hOR2AG1	8 11 12 15 16 18 19 51 54 55 58 59 62 63 65 66 67 81 82 85 86 88 89 90 92 93 151 171 172 173 175 180 183 184 187 188 237 238 241 249 250 251 253 254 255 257 258 259 260 261 262 264 265 266 269
hOR2M3	77 80 81 84 85 88 90 97 100 101 104 105 155 156 159 160 165 171 176 177 178 179 180 181 182 183 189 190 191 192 194 198 199 201 202 203 206 256 259 269 272 273 276 277 280
hOR7D4	24 27 28 31 69 70 73 77 78 81 82 84 85 86 88 101 104 105 107 108 111 112 159 165 175 176 179 186 187 189 190 191 192 193 194 196 198 199 202 203 206 207 251 252 255 256 259 262 263 266 267 268 269 272 273 274 275 276 277 279 280 283

REFERENCES

Angel, T.E., Chance, M.R., and Palczewski, K. (2009). Conserved waters mediate structural and functional activation of family A (rhodopsin-like) G protein-coupled receptors. *Proc Natl Acad Sci USA* 106(21), 8555-8560. doi: 10.1073/pnas.0903545106.

Baker, D., and Sali, A. (2001). Protein structure prediction and structural genomics. *Science* 294(5540), 93-96. doi: 10.1126/science.1065659.

Bakker, R.A., Jongejan, A., Sansuk, K., Hacksell, U., Timmerman, H., Brann, M.R., et al. (2008). Constitutively active mutants of the histamine H1 receptor suggest a conserved hydrophobic asparagine-cage that constrains the activation of class A G protein-coupled receptors. *Mol Pharmacol* 73(1), 94-103. doi: 10.1124/mol.107.038547.

Baranski, T.J., Herzmark, P., Lichtarge, O., Gerber, B.O., Trueheart, J., Meng, E.C., et al. (1999). C5a receptor activation. Genetic identification of critical residues in four transmembrane helices. *J Biol Chem* 274(22), 15757-15765. doi: 10.1074/jbc.274.22.15757

Beuming, T., and Sherman, W. (2012). Current assessment of docking into GPCR crystal structures and homology models: successes, challenges, and guidelines. *J Chem Inf Model* 52(12), 3263-3277. doi: 10.1021/ci300411b.

Biarnes, X., Marchiori, A., Giorgetti, A., Lanzara, C., Gasparini, P., Carloni, P., et al. (2010). Insights into the binding of Phenyltiocarbamide (PTC) agonist to its target human TAS2R38 bitter receptor. *PLoS One* 5(8), e12394. doi: 10.1371/journal.pone.0012394.

Changeux, J.P., and Edelstein, S. (2011). Conformational selection or induced fit? 50 years of debate resolved. *F1000 Biol Rep* 3, 19. doi: 10.3410/B3-19.

Charlier, L., Topin, J., de March, C.A., Lai, P.C., Crasto, C.J., and Golebiowski, J. (2013). Molecular modelling of odorant/olfactory receptor complexes. *Methods Mol Biol* 1003, 53-65. doi: 10.1007/978-1-62703-377-0_4.

Chothia, C., and Lesk, A.M. (1986). The relation between the divergence of sequence and structure in proteins. *EMBO J* 5(4), 823-826.

Colquhoun, D. (1998). Binding, gating, affinity and efficacy: the interpretation of structure-activity relationships for agonists and of the effects of mutating receptors. *Br J Pharmacol* 125(5), 924-947. doi: 10.1038/sj.bjp.0702164

D'Antona, A.M., Ahn, K.H., Wang, L., Mierke, D.F., Lucas-Lenard, J., and Kendall, D.A. (2006). A cannabinoid receptor 1 mutation proximal to the DRY motif results in constitutive activity and reveals intramolecular interactions involved in receptor activation. *Brain Res* 1108(1), 1-11. doi: 10.1016/j.brainres.2006.05.042.

Deupi, X., Edwards, P., Singhal, A., Nickle, B., Oprian, D., Schertler, G., et al. (2012). Stabilized G protein binding site in the structure of constitutively active metarhodopsin-II. *Proc Natl Acad Sci USA* 109(1), 119-124. doi: 10.1073/pnas.1114089108.

Dominguez, C., Boelens, R., and Bonvin, A.M. (2003). HADDOCK: a protein-protein docking approach based on biochemical or biophysical information. *J Am Chem Soc* 125(7), 1731-1737. doi: 10.1021/ja026939x.

Dunham, T.D., and Farrens, D.L. (1999). Conformational changes in rhodopsin. Movement of helix f detected by site-specific chemical labeling and fluorescence spectroscopy. *J Biol Chem* 274(3), 1683-1690. doi: 10.1074/jbc.274.3.1683

Duprez, L., Parma, J., Van Sande, J., Allgeier, A., Leclere, J., Schvartz, C., et al. (1994). Germline mutations in the thyrotropin receptor gene cause non-

autoimmune autosomal dominant hyperthyroidism. *Nat Genet* 7(3), 396-401. doi: 10.1038/ng0794-396.

Eramian, D., Eswar, N., Shen, M.Y., and Sali, A. (2008). How well can the accuracy of comparative protein structure models be predicted? *Protein Sci* 17(11), 1881-1893. doi: 10.1110/ps.036061.108.

Fanelli, F. (2000). Theoretical study on mutation-induced activation of the luteinizing hormone receptor. *J Mol Biol* 296(5), 1333-1351. doi: 10.1006/jmbi.2000.3516.

Fenalti, G., Giguere, P.M., Katritch, V., Huang, X.P., Thompson, A.A., Cherezov, V., et al. (2014). Molecular control of delta-opioid receptor signalling. *Nature* 506(7487), 191-196. doi: 10.1038/nature12944.

Friesner, R.A., Banks, J.L., Murphy, R.B., Halgren, T.A., Klicic, J.J., Mainz, D.T., et al. (2004). Glide: a new approach for rapid, accurate docking and scoring. 1. Method and assessment of docking accuracy. *J Med Chem* 47(7), 1739-1749. doi: 10.1021/jm0306430.

Fujiwara, Y., Osborne, D.A., Walker, M.D., Wang, D.A., Bautista, D.A., Liliom, K., et al. (2007). Identification of the hydrophobic ligand binding pocket of the S1P1 receptor. *J Biol Chem* 282(4), 2374-2385. doi: 10.1074/jbc.M609648200.

Gao, Z.G., Kim, S.K., Gross, A.S., Chen, A., Blaustein, J.B., and Jacobson, K.A. (2003). Identification of essential residues involved in the allosteric modulation of the human A(3) adenosine receptor. *Mol Pharmacol* 63(5), 1021-1031. doi: 10.1124/mol.63.5.1021

Geithe, C., Protze, J., Kreuchwig, F., Krause, G., and Krautwurst, D. (2017). Structural determinants of a conserved enantiomer-selective carvone binding pocket in the human odorant receptor OR1A1. *Cell Mol. Life Sci*, in press. doi: 10.1007/s00018-017-2576-z

Gelis, L., Wolf, S., Hatt, H., Neuhaus, E.M., and Gerwert, K. (2012). Prediction of a ligand-binding niche within a human olfactory receptor by combining site-directed mutagenesis with dynamic homology modeling. *Angew Chem Int Ed Engl* 51(5), 1274-1278. doi: 10.1002/anie.201103980.

Greasley, P.J., Fanelli, F., Rossier, O., Abuin, L., and Cotecchia, S. (2002). Mutagenesis and modelling of the alpha(1b)-adrenergic receptor highlight the role of the helix 3/helix 6 interface in receptor activation. *Mol Pharmacol* 61(5), 1025-1032. DOI: https://doi.org/10.1124/mol.61.5.1025

Han, M., Lou, J., Nakanishi, K., Sakmar, T.P., and Smith, S.O. (1997). Partial agonist activity of 11-cis-retinal in rhodopsin mutants. *J Biol Chem* 272(37), 23081-23085. doi: 10.1074/jbc.272.37.23081

Ho, J., Perez-Aguilar, J.M., Gao, L., Saven, J.G., Matsunami, H., and Eckenhoff, R.G. (2015). Molecular recognition of ketamine by a subset of olfactory G protein-coupled receptors. *Sci Signal* 8(370), ra33. doi: 10.1126/scisignal.2005912.

Holst, B., Nygaard, R., Valentin-Hansen, L., Bach, A., Engelstoft, M.S., Petersen, P.S., et al. (2010). A conserved aromatic lock for the tryptophan rotameric switch in TM-VI of seven-transmembrane receptors. *J Biol Chem* 285(6), 3973-3985. doi: 10.1074/jbc.M109.064725.

Hulme, E.C. (2013). GPCR activation: a mutagenic spotlight on crystal structures. *Trends Pharmacol Sci* 34(1), 67-84. doi: 10.1016/j.tips.2012.11.002.

Katritch, V., Fenalti, G., Abola, E.E., Roth, B.L., Cherezov, V., and Stevens, R.C. (2014). Allosteric sodium in class A GPCR signaling. *Trends Biochem Sci* 39(5), 233-244. doi: 10.1016/j.tibs.2014.03.002.

Katritch, V., Rueda, M., Lam, P.C., Yeager, M., and Abagyan, R. (2010). GPCR 3D homology models for ligand screening: lessons learned from blind predictions of adenosine A2a receptor complex. *Proteins* 78(1), 197-211. doi: 10.1002/prot.22507.

Kenakin, T. (2002). Drug efficacy at G protein-coupled receptors. *Annu Rev Pharmacol Toxicol* 42, 349-379. doi: 10.1146/annurev.pharmtox.42.091401.113012.

Kleinau, G., Jaeschke, H., Mueller, S., Worth, C.L., Paschke, R., and Krause, G. (2008). Molecular and structural effects of inverse agonistic mutations on signaling of the thyrotropin receptor--a basally active GPCR. *Cell Mol Life Sci* 65(22), 3664-3676. doi: 10.1007/s00018-008-8450-2.

Kosugi, S., Hai, N., Okamoto, H., Sugawa, H., and Mori, T. (2000). A novel activating mutation in the thyrotropin receptor gene in an autonomously functioning thyroid nodule developed by a Japanese patient. *Eur J Endocrinol* 143(4), 471-477. doi: 10.1530/eje.0.1430471

Kufareva, I., Rueda, M., Katritch, V., Stevens, R.C., Abagyan, R., and participants, G.D. (2011). Status of GPCR modeling and docking as reflected by community-wide GPCR Dock 2010 assessment. *Structure* 19(8), 1108-1126. doi: 10.1016/j.str.2011.05.012.

Labadi, A., Grassi, E.S., Gellen, B., Kleinau, G., Biebermann, H., Ruzsa, B., et al. (2015). Loss-of-Function Variants in a Hungarian Cohort Reveal Structural Insights on TSH Receptor Maturation and Signaling. *J Clin Endocrinol Metab* 100(7), E1039-1045. doi: 10.1210/jc.2014-4511.

Lancet, D., Sadovsky, E., and Seidemann, E. (1993). Probability model for molecular recognition in biological receptor repertoires: significance to the olfactory system. *Proc Natl Acad Sci USA* 90(8), 3715-3719. doi: 10.1073/pnas.90.8.3715

Laskowski, R.A., and Swindells, M.B. (2011). LigPlot+: multiple ligand-protein interaction diagrams for drug discovery. *J Chem Inf Model* 51(10), 2778-2786. doi: 10.1021/ci200227u.

Latorraca, N.R., Venkatakrishnan, A.J., and Dror, R.O. (2017). GPCR Dynamics: Structures in Motion. *Chem Rev* 117(1), 139-155. doi: 10.1021/acs.chemrev.6b00177.

Le Guilloux, V., Schmidtke, P., and Tuffery, P. (2009). Fpocket: an open source platform for ligand pocket detection. *BMC Bioinformatics* 10, 168. doi: 10.1186/1471-2105-10-168.

Lebon, G., Bennett, K., Jazayeri, A., and Tate, C.G. (2011). Thermostabilisation of an agonist-bound conformation of the human adenosine A(2A) receptor. *J Mol Biol* 409(3), 298-310. doi: 10.1016/j.jmb.2011.03.075.

Liu, R., Nahon, D., le Roy, B., Lenselink, E.B., and AP, I.J. (2015). Scanning mutagenesis in a yeast system delineates the role of the NPxxY(x)(5,6)F motif and helix 8 of the adenosine A(2B) receptor in G protein coupling. *Biochem Pharmacol* 95(4), 290-300. doi: 10.1016/j.bcp.2015.04.005.

Lu, Z.L., and Hulme, E.C. (1999). The functional topography of transmembrane domain 3 of the M1 muscarinic acetylcholine receptor, revealed by scanning mutagenesis. *J Biol Chem* 274(11), 7309-7315. doi: 10.1074/jbc.274.11.7309

Lubrano-Berthelier, C., Dubern, B., Lacorte, J.M., Picard, F., Shapiro, A., Zhang, S., et al. (2006). Melanocortin 4 receptor mutations in a large cohort of severely obese adults: prevalence, functional classification, genotype-phenotype relationship, and lack of association with binge eating. *J Clin Endocrinol Metab* 91(5), 1811-1818. doi: 10.1210/jc.2005-1411.

Madabushi, S., Gross, A.K., Philippi, A., Meng, E.C., Wensel, T.G., and Lichtarge, O. (2004). Evolutionary trace of G protein-coupled receptors reveals clusters of residues that determine global and class-specific functions. *J Biol Chem* 279(9), 8126-8132. doi: 10.1074/jbc.M312671200.

Marchiori, A., Capece, L., Giorgetti, A., Gasparini, P., Behrens, M., Carloni, P., et al. (2013). Coarse-Grained/Molecular Mechanics of the TAS2R38 Bitter Taste Receptor: Experimentally-Validated Detailed Structural Prediction of Agonist Binding. *Plos One* 8(5). doi: 10.1371/journal.pone.0064675.

Martin, S.S., Holleran, B.J., Escher, E., Guillemette, G., and Leduc, R. (2007). Activation of the angiotensin II type 1 receptor leads to movement of the sixth transmembrane domain: analysis by the substituted cysteine accessibility method. *Mol Pharmacol* 72(1), 182-190. doi: 10.1124/mol.106.033670.

Meyerhof, W., Batram, C., Kuhn, C., Brockhoff, A., Chudoba, E., Bufe, B., et al. (2010). The molecular receptive ranges of human TAS2R bitter taste receptors. *Chem Senses* 35(2), 157-170. doi: 10.1093/chemse/bjp092.

Nishihara, E., Fukata, S., Hishinuma, A., Kudo, T., Ohye, H., Ito, M., et al. (2006). Sporadic congenital hyperthyroidism due to a germline mutation in the thyrotropin receptor gene (Leu512Gln) in a Japanese patient. *Endocr J* 53(6), 735-740. DOI: 10.1210/jcem.82.11.4378

Nygaard, R., Valentin-Hansen, L., Mokrosinski, J., Frimurer, T.M., and Schwartz, T.W. (2010). Conserved Water-mediated Hydrogen Bond Network between TM-I, -II, -VI, and -VII in 7TM Receptor Activation. *J Biol Chem* 285(25), 19625-19636. doi: 10.1074/jbc.M110.106021.

Parma, J., Duprez, L., Van Sande, J., Hermans, J., Rocmans, P., Van Vliet, G., et al. (1997). Diversity and prevalence of somatic mutations in the thyrotropin receptor and Gs alpha genes as a cause of toxic thyroid adenomas. *J Clin Endocrinol Metab* 82(8), 2695-2701. doi: 10.1210/jcem.82.8.4144.

Parnot, C., Bardin, S., Miserey-Lenkei, S., Guedin, D., Corvol, P., and Clauser, E. (2000). Systematic identification of mutations that constitutively activate the angiotensin II type 1A receptor by screening a randomly mutated cDNA library with an original pharmacological bioassay. *Proc Natl Acad Sci USA* 97(13), 7615-7620. doi: 10.1073/pnas.110142297.

Piccoli, S., Suku, E., Garonzi, M., and Giorgetti, A. (2013). Genome-wide Membrane Protein Structure Prediction. *Curr Genomics* 14(5), 324-329. doi: 10.2174/13892029113149990009.

Porcellini, A., Ciullo, I., Laviola, L., Amabile, G., Fenzi, G., and Avvedimento, V.E. (1994). Novel mutations of thyrotropin receptor gene in thyroid hyperfunctioning adenomas. Rapid identification by fine needle aspiration biopsy. *J Clin Endocrinol Metab* 79(2), 657-661. doi: 10.1210/jcem.79.2.8045989.

Pydi, S.P., Singh, N., Upadhyaya, J., Bhullar, R.P., and Chelikani, P. (2014). The third intracellular loop plays a critical role in bitter taste receptor activation. *Biochim Biophys Acta* 1838 (1PtB), 231-236. doi: 10.1016/j.bbamem.2013.08.009.

Russo, D., Arturi, F., Suarez, H.G., Schlumberger, M., Du Villard, J.A., Crocetti, U., et al. (1996). Thyrotropin receptor gene alterations in thyroid hyperfunctioning adenomas. *J Clin Endocrinol Metab* 81(4), 1548-1551. doi: 10.1210/jcem.81.4.8636365.

Russo, D., Wong, M.G., Costante, G., Chiefari, E., Treseler, P.A., Arturi, F., et al. (1999). A Val 677 activating mutation of the thyrotropin receptor in a Hurthle cell thyroid carcinoma associated with thyrotoxicosis. *Thyroid* 9(1), 13-17. doi: 10.1089/thy.1999.9.13.

Sandal, M., Behrens, M., Brockhoff, A., Musiani, F., Giorgetti, A., Carloni, P., et al. (2015). Evidence for a Transient Additional Ligand Binding Site in the TAS2R46 Bitter Taste Receptor. *J Chem Theory Comput* 11(9), 4439-4449. doi: 10.1021/acs.jctc.5b00472.

Sansuk, K., Deupi, X., Torrecillas, I.R., Jongejan, A., Nijmeijer, S., Bakker, R.A., et al. (2011). A structural insight into the reorientation of transmembrane domains 3 and 5 during family A G protein-coupled receptor activation. *Mol Pharmacol* 79(2), 262-269. doi: 10.1124/mol.110.066068.

Shapiro, D.A., Kristiansen, K., Weiner, D.M., Kroeze, W.K., and Roth, B.L. (2002). Evidence for a model of agonist-induced activation of 5-hydroxytryptamine 2A serotonin receptors that involves the disruption of a strong ionic interaction

between helices 3 and 6. *J Biol Chem* 277(13), 11441-11449. doi: 10.1074/jbc.M111675200.
Singh, N., Pydi, S.P., Upadhyaya, J., and Chelikani, P. (2011). Structural basis of activation of bitter taste receptor T2R1 and comparison with Class A G-protein-coupled receptors (GPCRs). *J Biol Chem* 286(41), 36032-36041. doi: 10.1074/jbc.M111.246983.
Spalding, T.A., Burstein, E.S., Henderson, S.C., Ducote, K.R., and Brann, M.R. (1998). Identification of a ligand-dependent switch within a muscarinic receptor. *J Biol Chem* 273(34), 21563-21568. doi: 10.1074/jbc.273.34.21563
Spyrakis, F., Bidon-Chanal, A., Barril, X., and Luque, F.J. (2011). Protein flexibility and ligand recognition: challenges for molecular modeling. *Arch Biochem Biophys* 11(2), 192-210. doi: 10.2174/156802611794863571
Spyrakis, F., and Cavasotto, C.N. (2015). Open challenges in structure-based virtual screening: Receptor modeling, target flexibility consideration and active site water molecules description. *Arch Biochem Biophys* 583, 105-119. doi: 10.1016/j.abb.2015.08.002.
Strange, P.G. (2008). Agonist binding, agonist affinity and agonist efficacy at G protein-coupled receptors. *Br J Pharmacol* 153(7), 1353-1363. doi: 10.1038/sj.bjp.0707672.
Strange, P.G. (2010). Use of the GTPgammaS ([35S]GTPgammaS and Eu-GTPgammaS) binding assay for analysis of ligand potency and efficacy at G protein-coupled receptors. *Br J Pharmacol* 161(6), 1238-1249. doi: 10.1111/j.1476-5381.2010.00963.x.
Tao, Y.X. (2008). Constitutive activation of G protein-coupled receptors and diseases: insights into mechanisms of activation and therapeutics. *Pharmacol Ther* 120(2), 129-148. doi: 10.1016/j.pharmthera.2008.07.005.
Tao, Y.X., Abell, A.N., Liu, X., Nakamura, K., and Segaloff, D.L. (2000). Constitutive activation of G protein-coupled receptors as a result of selective substitution of a conserved leucine residue in transmembrane helix III. *Mol Endocrinol* 14(8), 1272-1282. doi: 10.1210/mend.14.8.0503.
Tate, C.G., and Schertler, G.F. (2009). Engineering G protein-coupled receptors to facilitate their structure determination. *Curr Opin Struct Biol* 19(4), 386-395. doi: 10.1016/j.sbi.2009.07.004.
Tonacchera, M., Chiovato, L., Pinchera, A., Agretti, P., Fiore, E., Cetani, F., et al. (1998). Hyperfunctioning thyroid nodules in toxic multinodular goiter share activating thyrotropin receptor mutations with solitary toxic adenoma. *J Clin Endocrinol Metab* 83(2), 492-498. doi: 10.1210/jcem.83.2.4559.
Trott, O., and Olson, A.J. (2010). AutoDock Vina: improving the speed and accuracy of docking with a new scoring function, efficient optimization, and multithreading. *J Comput Chem* 31(2), 455-461. doi: 10.1002/jcc.21334.
Trulzsch, B., Krohn, K., Wonerow, P., Chey, S., Holzapfel, H.P., Ackermann, F., et al. (2001). Detection of thyroid-stimulating hormone receptor and Gsalpha mutations: in 75 toxic thyroid nodules by denaturing gradient gel electrophoresis. *J Mol Med (Berl)* 78(12), 684-691. DOI: 10.1007/s001090000170
Urizar, E., Claeysen, S., Deupi, X., Govaerts, C., Costagliola, S., Vassart, G., et al. (2005). An activation switch in the rhodopsin family of G protein-coupled receptors: the thyrotropin receptor. *J Biol Chem* 280(17), 17135-17141. doi: 10.1074/jbc.M414678200.
Vaisse, C., Clement, K., Durand, E., Hercberg, S., Guy-Grand, B., and Froguel, P. (2000). Melanocortin-4 receptor mutations are a frequent and heterogeneous cause of morbid obesity. *J Clin Invest* 106(2), 253-262. doi: 10.1172/JCI9238.
Valentin-Hansen, L., Holst, B., Frimurer, T.M., and Schwartz, T.W. (2012). PheVI:09 (Phe6.44) as a sliding microswitch in seven-transmembrane (7TM) G protein-

coupled receptor activation. *J Biol Chem* 287(52), 43516-43526. doi: 10.1074/jbc.M112.395137.

Venkatakrishnan, A.J., Deupi, X., Lebon, G., Heydenreich, F.M., Flock, T., Miljus, T., et al. (2016). Diverse activation pathways in class A GPCRs converge near the G-protein-coupling region. *Nature* 536(7617), 484-487. doi: 10.1038/nature19107.

Venkatakrishnan, A.J., Deupi, X., Lebon, G., Tate, C.G., Schertler, G.F., and Babu, M.M. (2013). Molecular signatures of G-protein-coupled receptors. *Nature* 494(7436), 185-194. doi: 10.1038/nature11896.

Williams, C., and Hill, S.J. (2009). GPCR signaling: understanding the pathway to successful drug discovery. *Methods Mol Biol* 552, 39-50. doi: 10.1007/978-1-60327-317-6_3.

Xu, W., Ozdener, F., Li, J.G., Chen, C., de Riel, J.K., Weinstein, H., et al. (1999). Functional role of the spatial proximity of Asp114(2.50) in TMH 2 and Asn332(7.49) in TMH 7 of the mu opioid receptor. *FEBS Lett* 447(2-3), 318-324. DOI: 10.1016/S0014-5793(99)00316-6

V Isberg, C de Graaf, A Bortolato, V Cherezov, V Katritch, F Marshall, S Mordalski, et al. (2015). Generic GPCR Residue Numbers - Aligning Topology Maps While Minding The Gaps", *Trends Pharmacol Sci*, 36(1), 22–31. doi: 10.1016/j.tips.2014.11.001
